# A multiscale model of epigenetic heterogeneity-driven cell fate decision-making

**DOI:** 10.1371/journal.pcbi.1006592

**Published:** 2019-04-30

**Authors:** Núria Folguera-Blasco, Rubén Pérez-Carrasco, Elisabet Cuyàs, Javier A. Menendez, Tomás Alarcón

**Affiliations:** 1 Centre de Recerca Matemàtica, Edifici C, Campus de Bellaterra, 08193 Bellaterra, Barcelona, Spain; 2 Departament de Matemàtiques, Universitat Autònoma de Barcelona, 08193 Bellaterra, Barcelona, Spain; 3 Department of Mathematics, University College London, Gower Street, London WC1E 6BT, UK; 4 ProCURE (Program Against Cancer Therapeutic Resistance), Metabolism and Cancer Group, Catalan Institute of Oncology, Girona, Spain; 5 Girona Biomedical Research Institute (IDIBGI), Girona, Spain; 6 ICREA, Pg. Lluís Companys 23, 08010 Barcelona, Spain; 7 Barcelona Graduate School of Mathematics (BGSMath), Barcelona, Spain; University of California Irvine, UNITED STATES

## Abstract

The inherent capacity of somatic cells to switch their phenotypic status in response to damage stimuli *in vivo* might have a pivotal role in ageing and cancer. However, how the entry-exit mechanisms of phenotype reprogramming are established remains poorly understood. In an attempt to elucidate such mechanisms, we herein introduce a stochastic model of combined epigenetic regulation (ER)-gene regulatory network (GRN) to study the plastic phenotypic behaviours driven by ER heterogeneity. To deal with such complex system, we additionally formulate a multiscale asymptotic method for stochastic model reduction, from which we derive an efficient hybrid simulation scheme. Our analysis of the coupled system reveals a regime of tristability in which pluripotent stem-like and differentiated steady-states coexist with a third indecisive state, with ER driving transitions between these states. Crucially, ER heterogeneity of differentiation genes is for the most part responsible for conferring abnormal robustness to pluripotent stem-like states. We formulate epigenetic heterogeneity-based strategies capable of unlocking and facilitating the transit from differentiation-refractory (stem-like) to differentiation-primed epistates. The application of the hybrid numerical method validates the likelihood of such switching involving solely kinetic changes in epigenetic factors. Our results suggest that epigenetic heterogeneity regulates the mechanisms and kinetics of phenotypic robustness of cell fate reprogramming. The occurrence of tunable switches capable of modifying the nature of cell fate reprogramming might pave the way for new therapeutic strategies to regulate reparative reprogramming in ageing and cancer.

## Author summary

Certain modifications of the structure and functioning of the protein/DNA complex called chromatin can allow adult, fully differentiated, cells to adopt a stem cell-like pluripotent state in a purely epigenetic manner, not involving changes in the underlying DNA sequence. Such reprogramming-like phenomena may constitute an innate reparative route through which human tissues respond to injury and could also serve as a novel regenerative strategy in human pathological situations in which tissue or organ repair is impaired. However, it should be noted that *in vivo* reprogramming would be capable of maintaining tissue homeostasis provided the acquisition of pluripotency features is strictly transient and accompanied by an accurate replenishment of the specific cell types being lost. Crucially, an excessive reprogramming in the absence of controlled re-differentiation would impair the repair or the replacement of damaged cells, thereby promoting pathological alterations of cell fate. A mechanistic understanding of how the degree of chromatin *plasticity* dictates the reparative versus pathological behaviour of *in vivo* reprogramming to *rejuvenate* aged tissues while preventing tumorigenesis is urgently needed, including especially the intrinsic epigenetic heterogeneity of the tissue resident cells being reprogrammed. We here introduce a novel method that mathematically captures how epigenetic heterogeneity is actually the driving force that governs the routes and kinetics to entry into and exit from a pathological stem-like state. Moreover, our approach computationally validates the likelihood of unlocking chronic, unrestrained plastic states and drive their differentiation down the correct path by solely manipulating the intensity and direction of few epigenetic control switches. Our approach could inspire new therapeutic approaches based on *in vivo* cell reprogramming for efficient tissue regeneration and rejuvenation and cancer treatment.

## Introduction

The ability of the ageing process to interfere with the robustness of the epigenetic regulation (ER) of differentiated phenotypes might suffice to promote tissue dysfunction and malignization [1].

**Fig 1:**
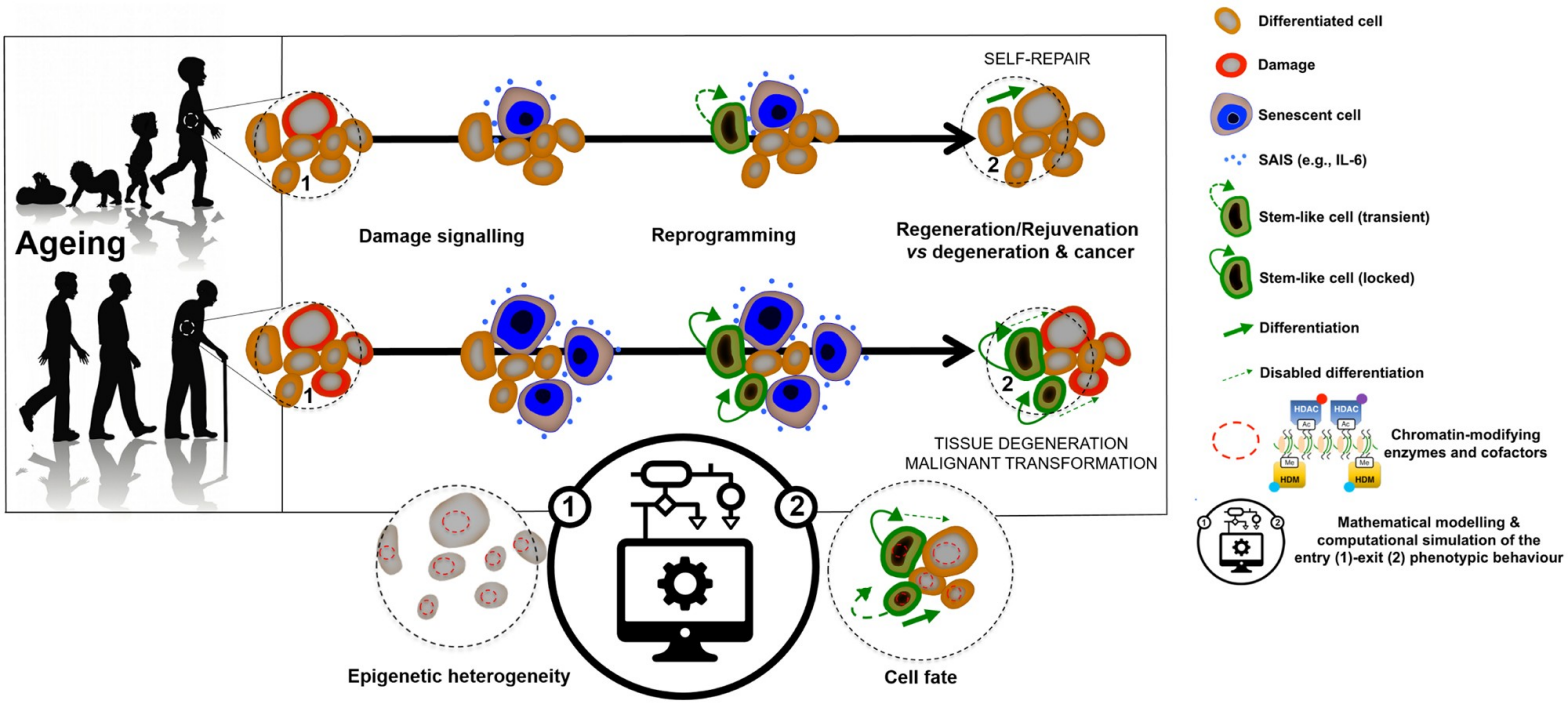
Physiological and pathological cell fate reprogramming: A mathematical approach. Reprogramming-like phenomena in response to damage signalling may constitute a reparative route through which human tissues respond to injury, stress, and disease via induction of a transient acquisition of epigenetic plasticity and phenotypic malleability. However, tissue regeneration/rejuvenation should involve not only the transient epigenetic reprogramming of differentiated cells, but also the committed re-acquisition of the original or alternative committed cell fate. Chronic or unrestrained epigenetic plasticity would drive ageing/cancer phenotypes by impairing the repair or the replacement of damaged cells; such uncontrolled phenomena of *in vivo* reprogramming might also generate cancer-like cellular states. Accordingly, we now know that chronic senescence-associated inflammatory signalling (SAIS) might lock cells in highly plastic epigenetic states disabled for reparative differentiation and prone to malignant transformation. We herein introduce a first-in-class stochastic, multiscale reduction method of combined epigenetic regulation (ER)-gene regulatory network (GRN) to mathematically model and computationally simulate how ER heterogeneity regulates the entry-exit mechanisms and kinetics of physiological and pathological cell fate reprogramming.

Fully committed somatic cells can spontaneously reprogram to pluripotent stem-like cells during the normal response to injury or damage *in vivo* [2]. Such cellular processes involving dedifferentiation and cell-fate switching might constitute a fundamental element of a tissue’s capacity to self-repair and rejuvenate [3, 4]. However, such physiological/reparative cell reprogramming might have pathological consequences if the acquisition of epigenetic and phenotypic plasticity is not transient. In response to chronically permissive tissue environments for *in vivo* reprogramming, the occurrence of unrestrained epigenetic plasticity might permanently lock cells into self-renewing pluripotent cell states disabled for reparative differentiation and prone to tumorigenesis (see Fig 1) [1, 5–8].

Central to such so-called stem-lock model for ageing and cancer [6, 9, 10] is the sufficient capacity of ER to drive cell fate in the absence of *bona fide*, initiating events. ER refers to a series of modifications of the cell’s DNA without modifying its genetic sequence. Such modifications can disrupt or allow expression of particular genes. By switching on or off different parts of the genome, ER is in fact responsible for the variety of phenotypes in complex multicellular organisms (where all somatic cells are genetically identical). Recent advances in experimental determination of ER mechanisms have triggered an ever-growing interest in developing mathematical models regarding both ER of gene expression [11–16] and epigenetic memory [12–14, 17–20]. Identification of the molecular culprits underlying the ER capacity to drive the transition between normal and highly restrictive/permissive chromatin states is expected to have major impact in the understanding and therapeutic management of ageing-related diseases including cancer [7, 21, 22]. Unfortunately, robust and standardised approaches capable of capturing such fundamental stochastic aspects of ageing biology are mostly lacking.

Here, we present a mathematical and computational systems biology approach capable of deconstructing, modelling and simulating the predictive power that ER may have on the susceptibility of cells to loss (and re-gain) their normal identity. By adding ER to the picture, our current work significantly extends previous approaches where phenotypes are associated with the attractors of complex gene regulatory systems and their robustness, with the resilience of such attractors in the presence of intrinsic noise, environmental fluctuations, and other disturbances [23–31]. Specifically, we develop a stochastic model of a coupled ER-gene regulatory network (GRN) system aimed at analysing the impact of ER heterogeneity on the causal relationship between epigenetic plasticity and cell-fate reprogramming and determination. Moreover, by introducing a stochastic model reduction analysis based on multiple scale asymptotics of the combined ER-GRN system [32–39], we provide our model with the capacity of evaluating a variety of phenotypic behaviours due solely to ER systems heterogeneity [40].

This work is organised as follows. In section *Materials and methods*, we present a summary of the formulation of our ER-GRN model and its analysis. First, we present the general description of an ER-GRN model, and later, we focus on the ER component of the model. We detail how the transitions between stable states within the ER system are computed and how the ensemble of ER systems is generated. In order to analyse the consequences of the existing heterogeneity within the ER systems generated, we develope a multiscale asymptotic theory to study ER-GRN systems (for which additional details are given in the S1 Text). Such theory allows us to reduce the complexity of the model to a hybrid system, for which a numerical simulation method was implemented (see S1 Appendix). The *Results* section starts by presenting the findings regarding the effects of ER in the GRN. In particular, we initially evaluate the epigenetic parameters regulating the entry into robust epigenetic states throughout the entire ER-GRN system. Then, we focus on the role that the ER heterogeneity may have in giving rise to different system behaviours, namely, differentiation-primed and differentiation-resilient (pluripotency-locked) states. Once these two different behaviours are identified, we perform an analysis to identify the mechanisms regulating the phenotypic robustness of the pluripotency-locked and differentiation-primed states. We then formulate epigenetic heterogeneity-based strategies capable of directing the exit and transit from stem-locked to differentiation-primed epistates. We finally apply the hybrid numerical method derived from our theoretical analysis to determine the efficiency of the epigenetic strategies formulated to unlock a persistent state of pathological pluripotency. Finally, in the *Discussion* section, we summarise our findings and present our conclusions.

## Materials and methods

### Model formulation and analysis

**Fig 2:**
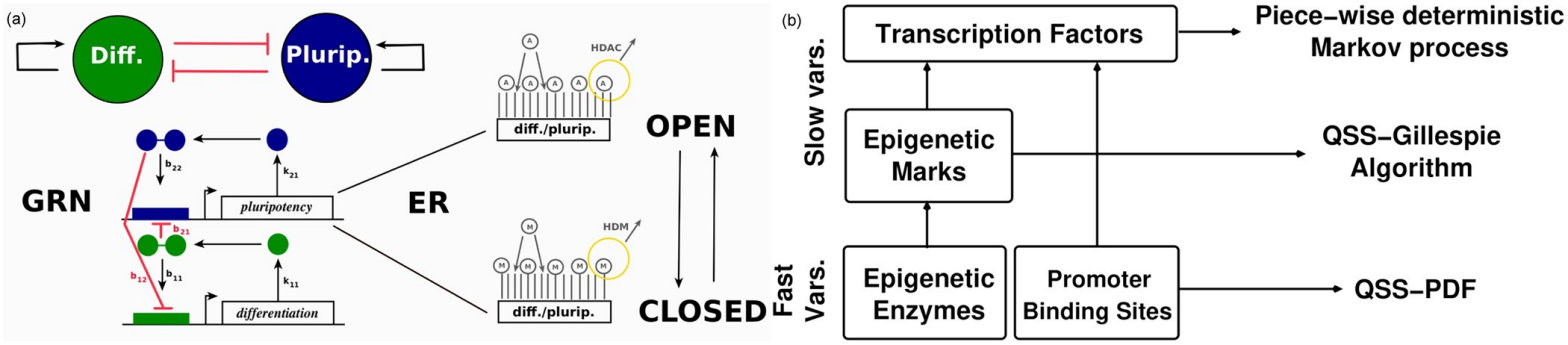
Schematic reprentation of the ER-GRN model and its multiscale reduction. (a): Gene regulatory network (GRN) of two self-activating, mutually-inhibitory genes with epigenetic regulation (ER). In the GRN model, the gene product (single circle, denoted by *X*_*i*_ in S2 Table) is its own transcription factor which, upon dimerisation (two joint circles), binds the promoter region of the gene thus triggering gene transcription. The transition rates corresponding to this GRN are given in S2 Table. For simplicity, we use an effective model in which the formation of the dimer and binding to the promoter region is taken into account in a single reaction, and the resulting number of promoter sites bound by two transcription factors is denoted by *X*_*ij*_ (see S2 Table). Furthermore, depending on whether the epigenetic state is open (i.e. predominantly acetylated (A)) or closed (i.e. predominantly methylated (M)) the promoter region of the gene is accessible or inaccessible to the transcription factor, respectively. (b): Schematic representation of the time separation structure of the multiscale method developed to simulate the ER-GRN system. See text and S1 Text for more details.

In this paper, we aim to study an ER-GRN model which can describe cell differentiation and cell reprogramming. One of the simplest GRNs which allows to study such situation consists of two genes, one promoting differentiation, and the other promoting pluripotency (see Fig 2(a)). Nevertheless, in this section, we formulate and analyse our model considering a generic case with *N*_*G*_ genes. By doing so, our theoretical analysis can be further applied to any ER-GRN model, which implies a wide applicability of the derived formulation. However, when possible, we try to relate the theory developed to our particular ER-GRN so as to keep track of our case study.

### General description of the stochastic model of an epigenetically-regulated gene regulatory network

Consider a gene regulatory network composed of *N*_*G*_ self-activating genes which can repress each other. In particular, we consider that the gene product of each of these genes forms homodimers, which act as a transcription factor (TF) for its own gene by binding to its own promoter. Furthermore, each gene within the network has a number of inhibitors, which operate via competitive inhibition: the homodimers of protein *j* bind to the promoter of gene *i*, and by doing so they impede access of the TF to the promoter of gene *i*. In Fig 2(a), an illustrative scheme of the simplified case of two mutually inhibiting genes, one promoting pluripotency (blue) and one promoting differentiation (green), is shown. The regulation topology of the network can be represented by the binding rates of homodimers of protein *j* to the promoter of gene *i*, *b*_*ij*_ > 0 (see Fig 2(a)). Moreover, the expression of gene *i* is induced at a constant basal production rate, ${\hat{R}}_{i}$, independent of the regulatory mechanism described above. Proteins (TF monomers) of type *i* are synthesised at a rate proportional to the number of bound promoter sites with rate constant *k*_*i*1_ and degraded with degradation rate *k*_*i*2_ (see Fig 2(a) and S2 Table).

In addition to TF regulation, we further consider that each gene is under ER. ER controls gene transcription by modulating access of TFs to the promoter regions of the genes. In other words, in our model, ER is associated with an upstream drive that regulates gene expression [41]. Such epigenetic control is often related to alternative covalent modifications of histones. To address the high complexity of ER, we focus on a simpler stochastic model of ER, based on that formulated in [11, 16] and [17]. Our model belongs to a wider class of models which consider that single unmodified (U) chromatin loci can be modified so as to acquire positive (A) or negative (M) marks. Of such modifications, we consider methylation (associated with negative marks) and acetylation (associated with positive marks) [17]. An illustrative example on how epigenetic modifications alter the accessibility of TFs to the promoter regions of the genes is shown in Fig 2(a). Depending on whether the promoter region of a gene is mainly acetylated (A) or methylated (M), that promoter region is accessible (*open*) or inaccessible (*closed*) to the TFs, respectively. Both modifications are mediated by epigenetic enzymes: histone methylases (HMs) and demethylases (HDMs), and histone acetylases (HACs) and deacetylases (HDACs), which add or remove methylation and acetylation marks, respectively. Following [16], we explicitly account for HDM and HDAC activity only (see Fig 2(a)). In our model, a positive feedback mechanism is introduced whereby M marks help to both add more M marks and remove A marks from neighbouring loci [17]. The positive marks are assumed to be under the effects of a similar positive reinforcement mechanism [14, 17]. In this framework, each ER state is defined by the vector (*Y*_*i*1_, …, *Y*_*i*7_), describing the abundance of epigenetic marks and epigenetic enzymes at a given time. A full description of the details of the ER model are given in Section *Stochastic model of epigenetic regulation* of the S1 Text (see also [16]) and S3 Table, where the transition rates for the ER model are provided.

Under suitable conditions, determined by the activity and abundance of histone-modifying enzymes and co-factors, the positive reinforcement mechanism produces robust bistable behaviour. In this bistable regime, the two possible ER stable states are an *open* epigenetic state and a *silenced* epigenetic state. In the *open* epigenetic state, the levels of positive (negative) marks are elevated (downregulated). In this case, the promoter of the gene is accessible to TFs and transcription can occur. By contrast, in the absence (abundance) of positive (negative) marks, the gene is considered to be *silenced*, as TFs cannot reach the promoter.

An essential part of the stochastic dynamics of the ER system is the noise-induced transitions between the open and silenced states. Escape from steady states is a well-established phenomenon (see e.g. [42]) and thoroughly analysed within the theory of rate processes [43] and large deviation theory [28, 44, 45]. As we will illustrate below, these noise-induced dynamics are essential to classify the epiphenotypes of somatic cells [16] and stem cells and to unravel the mechanisms of reprogramming and locking.

### Transitions between ER states: Minimum action path approach

Noise-induced transitions are essential to understand ER dynamics and their effect on cell-fate determination [46]. Throughout the bistable regime, sufficiently large fluctuations in the stochastic ER system will induce switching between the open and silenced states. The rate at which such transitions occur can be described using reaction-rate theory [43] and large deviation theory [44], which show that the waiting time between transitions is exponentially distributed. The average switching time, *τ*_*s*_, increases exponentially with system size, which in this case is given by the scale of ER substrates, *Y* [44, 45, 47, 48]:

$$\begin{array}{c} \tau_{s}=Ce^{YS}, \end{array}$$ (1)

where *C* is a constant and S is the minimum action of the stochastic switch. Eq (1) is derived from considering the probability distribution of the so-called *fluctuation paths*, *φ*(*τ*), in the ER space (*Y*_*i*1_, …, *Y*_*i*7_), which connect the mean-field steady states in a time *τ*. According to large deviation theory [44, 45], we have $P(\varphi (\tau ))∼e^{-YA_{FW}\left(\varphi \left(\tau \right)\right)}$, which implies that the probability of observing paths different from the optimal, i.e. the path φ_*_ that minimises the Freidlin-Wentzel (FW) action functional $A_{FW}$, is exponentially supressed as system size, *Y*, increases. This means that, for a large enough system size, the behaviour of the system regarding large fluctuations is characterised by the optimal path:

$$\begin{array}{c} S\equiv A_{FW}(\varphi_{*})=\min_{\tau ,\varphi (\tau )}A_{FW}(\varphi (\tau )). \end{array}$$ (2)

An explicit form of the functional $A_{FW}\left(\varphi \left(\tau \right)\right)$ (see S1 Text) can be given if the dynamics is described by the corresponding chemical Langevin equation [49]. In this case, the optimal value of the action, S, can be found by numerical minimisation, which provides both the optimal or minimum action path (MAP), φ_*_, and the rate at which the ER system switches state driven by intrinsic noise [28]. Details regarding implementation of the action-optimisation algorithm are given in Section *Summary of the minimum action path theory and numerical method* of the S1 Text. A complete description of *τ*_*s*_ requires to estimate the pre-factor *C* (see Eq (1)), which is not provided by the FW theory, but can be easily estimated using stochastic simulation.

### ER-system ensemble generation and parameter sensitivity analysis

In order to compute the switching time between the open and closed states (and vice versa) of the ER system, we should consider a particular ER system for each gene within the network. In order to mimic the existing ER heterogeneity within a set of cells from a particular tissue, we generated an ensemble of ER systems for each gene, which can be used to identify properties at population level. Such an ensemble is generated using approximate Bayesian computation (ABC) [50, 51], whereby we generate an ensemble of parameter sets {*c*_*ij*_|*i* = 1, …, *N*_*G*_; *j* = 1, …, 16}, with *c*_*ij*_ the kinetic parameter of the *j*-th reaction of the ER model for the gene *i* (see S3 Table), compatible with simulated data for the epigenetic regulation systems. The generated kinetic rate constants are dimensionless, i.e. they are relative to a given rate scale [16]. Such a feature implies that there is an undetermined time scale in our system. This additional degree of freedom can be used to fit our model of epigenetic (de-)activation to particular data. Since the global time scales associated with different ER systems may differ among them, our model has the capability of reproducing different systems characterised by different time scales as previously shown by Bintu et al. [12].

Our approach follows closely that of [16], to which readers are referred to for a detailed presentation of the implementation. To summarise, we start by generating synthetic (simulated) data (denoted as “raw data” in S1 Fig) for the ER system of a genetic network epigenetically poised for differentiation, i.e. open differentiation-promoting genes and silenced pluripotency-promoting genes (see the example shown in S1 Fig). These raw data will play the role of the experimental data, *x*_0_, to which we wish to fit our model. The raw data set consists of 10 realisations and 25 time points per realisation for each of the *N*_*G*_ epigene regulatory systems. For each time point, *t*_*i*_, we consider two summary statistics: the mean over realisations, $\bar{x}\left(t_{i}\right)$, and the associated standard deviation, *σ*(*t*_*i*_). We then run the ABC rejection sampler method until we reach an ensemble of 10000 parameter sets which fit the raw data, *x*_0_, within prescribed tolerances for the mean and standard deviation. We generate a 10000 parameter set ensemble of the ER system for the pluripotency-promoting gene and another 10000 parameter set ensemble for the gene promoting differentiation. In order to illustrate the effectiveness of the ABC method, S1(a) & S1(b) Fig show results comparing the reference (raw simulated) data to a subensemble average consisting of the 100 sets that best fit the data, for the differentiation- and pluripotency-promoting genes, respectively.

The above procedure provides us with an ensemble of parameter sets {*c*_*ij*_} that are compatible with our raw data, i.e. such that they fit the data within the prescribed tolerances. The heterogeneity within this ensemble is compatible with existing biological variability in the activity of the different enzymes that carry out the epigenetic-regulatory modifications (HDMs, HDACs, as well as, histone methylases (HMs) and histone acetylases (HACs)), so that variation in {*c*_*ij*_} can be traced back to heterogeneity in the availability of cofactors, many of them of metabolic origin such as NAD+, which are necessary for these enzymes to perform their function [16].

Our ensemble method also allows parameter sensitivity analysis regarding robustness of different ER behaviours, as described in detail in [16]. Once we have generated the ensemble, we identify subsets exhibiting certain properties of potential biological interest (e.g. mono- *vs* bistability) and perform a comparison between the parameter sets belonging to each of the subensembles, as well as a comparison of each subensemble with the whole ensemble. Such approach allows an identification of the essential parameters (via comparison of their empirical cumulative distribution functions (CDFs)) required by the system to exhibit the dynamic behaviour associated with a particular subensemble. A parameter is deemed as significant for a given behaviour when statistically significant differences can be detected between the subensemble-specific CDF and the CDF of other subensembles (or the whole population). The CDFs’ shapes provide also useful information as to how the significant parameter should be changed for the ER system to switch a given behaviour. More details are given later in the *Results* Section.

### Coupling ER-GRN models: Multiscale analysis and model reduction

One should acknowledge that the system that results from coupling the ER and GRN models becomes rather cumbersome and computationally intractable as the GRN grows. We therefore took advantage of the intrinsic separation of time scales to analyse the behaviour of the resulting stochastic model [32–39]. Such approach allowed us to achieve meaningful model reduction via stochastic quasi-steady state approximations (QSSA) involving asymptotic analysis of the stochastic ER-GRN system [34–36, 38] (see Fig 2(b)). We assumed that the characteristic scale for the number of TF monomers (*S*), the number of promoter binding sites (*E*), the number of ER modification sites (*Y*), and the number of ER enzymes (*Z*), are such that *S* ≫ *E*, *Y* ≫ *Z* and O(E)=O(Y) (see S1 Table for the definition of these variables). It is noteworthy that the assumption *Y* ≫ *Z* is exactly the Briggs-Haldane hypothesis for enzyme kinetics [52] because the ER modification sites are the substrates for the ER enzymes (see Section *Stochastic model of epigenetic regulation* in the S1 Text). The multiscale analysis and its technical details are provided in Sections *Multiscale analysis of the GRN system: WKB approximation and multiscale optimal path theory* and *Stochastic model reduction method* of the S1 Text. Furthermore, the corresponding numerical method derived from this stochastic model reduction is described in S1 Appendix.

Under appropriate assumptions regarding the characteristic scales of the different molecular species, our model exhibits a hierarchy of time scales, thereby allowing a model simplification and its computational simulation. Since *S* ≫ *E* and *Y* ≫ *Z*, the number of bound-to-promoter TFs and ER enzyme-substrate complexes are fast variables that can be sampled from their quasi-equilibrium distribution with respect (or conditioned to) their associated slow variables. TFs and ER modification sites (i.e., ER substrates) are slow variables whose dynamics, which dominate the long-time behaviour of the system, are given by their associated stochastic dynamics with the fast variables sampled from their quasi-steady state approximation (QSSA) probability density functions (PDFs).

The assumption that *S* ≫ *Y* allows for additional simplification of the model, as it enables to take the limit of *S* ≫ 1 in the stochastic equations for the TFs monomers, which leads to a *piece-wise deterministic* Markov description where the dynamics of the number of TFs monomers is given by an ordinary differential equation (ODE) perturbed at discrete times by a noise source [36]. See Fig 2(b) for a schematic representation of the different techniques applied in this multiscale method leading to the numerical method applied in the *Results* section. After applying the asymptotic model reduction presented in detail in Section *Stochastic model reduction method* of the S1 Text, the resulting model equations of the full ER-GRN system reduce to:

$$\begin{array}{ccc} & & \frac{dx_{i}}{d\tau }=R_{i}+\omega_{i1}x_{ii}-\omega_{i2}x_{i}-2\sum_{j=1}^{N_{G}}(\beta_{ij}\eta_{i}(\frac{e_{j}}{E}-\sum_{k=1}^{N_{G}}x_{jk})x_{i}^{2}-\delta_{ij}x_{ij}),\;i=1,\ldots ,N_{G} \end{array}$$ (3)

$$\begin{array}{ccc} & & y_{ij}=y_{ij}\left(0\right)+\sum_{k=1}^{R_{E}}r_{E_{ijk}}\frac{1}{E}P(E\frac{1}{\epsilon_{2}}\int_{0}^{\tau }v_{ik}\left(y_{i}\left(\sigma \right)\right)d\sigma ),\;j=1,2,3, \end{array}$$ (4)

with *η*_*i*_ coupling the system, as it is defined as *η*_*i*_ = *H*(*y*_*i*3_ − *y*_0_), i.e. gene *i* is epigenetically open if the corresponding level of acetylation *y*_*i*3_ exceeds the threshold *y*_0_. The remaining variables and parameters are in S1–S3 Tables.

The resulting dynamics consists of a coupled hybrid system where the dynamics of the TF monomers, *x*_*i*_(*τ*) (Eq (3)) is described in terms of a piece-wise deterministic Markov process [53, 54], i.e., by a system of ODEs perturbed at discrete times by two random processes, one corresponding to stochastic ER (Eq (4)) and the other to TF dimers binding to the promoter regions, *x*_*jk*_. The latter are sampled from their QSSA PDFs (Eq. (S.51) in the S1 Text). The stochastic dynamics of the slow ER variables, Eq (4), is in turn coupled to the random variation of the associated fast variables (ER enzymes, HDM and HDAC, and complexes). The number of complexes, *Y*_*i*5_ and *Y*_*i*7_, are also sampled from their QSSA PDFs (Eqs. (S.53)-(S.54) in the S1 Text). We refer to this method as a hybrid method, since it involves coupling both types of mathematical descriptions and its numerical implementation, namely, the coupling between ODE solvers and stochastic simulation methods. The corresponding numerical method used to simulate such system is described in detail in S1 Appendix.

The computational time needed by the Gillespie algorithm (used to perform the stochastic simulations) increases linearly with the number of reactions in the system. Our model reduction method increases computational efficiency by reducing the number of (stochastic) reactions involved in the dynamics of the system (as illustrated in Fig. D in the S1 Text). Whilst in the example been considered (i.e. a two-gene regulatory system) this reduction is significant, such increase in efficiency might not be of enough significance when using larger GRNs. In such cases, the model reduction method should be supplemented with a Next Reaction Method implementation of the stochastic dynamics of the fast variables [55] or, alternatively, by approximating such stochastic dynamics using either the *τ*-leaping method [49] or the Chemical Langevin Equation (CLE) method [56]. In the latter case, a careful error analysis will be required when coupling the ODE solver for the slow variables, Eq (4), with the *τ*-leaping or CLE simulations.

## Results

In order to focus our discussion, we study a gene regulatory circuit with two genes, one that promotes differentiation and another one that induces pluripotency. These two genes are further assumed to interact through mutual competitive inhibition and to be under the effects of epigenetic regulation (see Fig 2(a)).

We proceed to analyse how ER sculpts the epigenetic landscape over the substrate of the phase space given by the GRN model. The latter provides the system with a variety of cell fates, corresponding to the stable steady states of the dynamical system underpinning the model of gene regulatory network [58]. The transitions between such cellular states, both deterministic and stochastic, depend upon the ability of the cell regulatory systems to elevate or lower the barriers between them. Epigenetic regulation is one of such mechanisms able to alter these barriers. Here, we show that the intrinsic ER heterogeneity within the ensemble generated—understood to be originated by variations in the availability of the co-factors necessary for histone modifying enzymes (HMEs) to carry out their chromatin-modifying functions—suffices to produce a variety of behaviours, including differentiation-primed and stem-locked states.

### The GRN model exhibits a complex phase space, including an undecided regulatory state

We start our analysis by studying the phase space of the dynamical system underlying our model of gene regulation, schematically illustrated in Fig 2(a). Using the methodology described in detail in Section *Multiscale analysis of the GRN system: WKB approximation and multiscale optimal path theory* of the S1 Text, we have derived the (quasi-steady state approximation) equations for the optimal path theory of the stochastic model of the mutually inhibitory two-gene system [11]. Such equations describe the most likely relaxation trajectories towards their steady states [47, 48], under conditions of time scale separation:

$$\begin{array}{ccc} & & \frac{dq_{1}}{d\tau }=R_{1}+p_{\infty_{1}}p\frac{\omega_{11}\frac{\beta_{11}}{\delta_{11}}q_{1}^{2}}{1+\frac{\beta_{11}}{\delta_{11}}q_{1}^{2}+\frac{\beta_{12}}{\delta_{12}}q_{2}^{2}}-\omega_{12}q_{1} \end{array}$$ (5)

$$\begin{array}{ccc} & & \frac{dq_{2}}{d\tau }=R_{2}+p_{\infty_{2}}p\frac{\omega_{21}\frac{\beta_{22}}{\delta_{22}}q_{2}^{2}}{1+\frac{\beta_{21}}{\delta_{21}}q_{1}^{2}+\frac{\beta_{22}}{\delta_{22}}q_{2}^{2}}-\omega_{22}q_{2} \end{array}$$ (6)

where *q*_1_ and *q*_2_ are the variables (generalised coordinates) associated with the number of molecules of proteins, *X*_1_ and *X*_2_, related to differentiation and pluripotent behaviours, respectively. The re-scaled variables, *q*_*i*_ and *q*_*ij*_, and the re-scaled parameters, *ω*_*ij*_, *β*_*ij*_, and *δ*_*ij*_, are defined in S2 Table.

**Fig 3:**
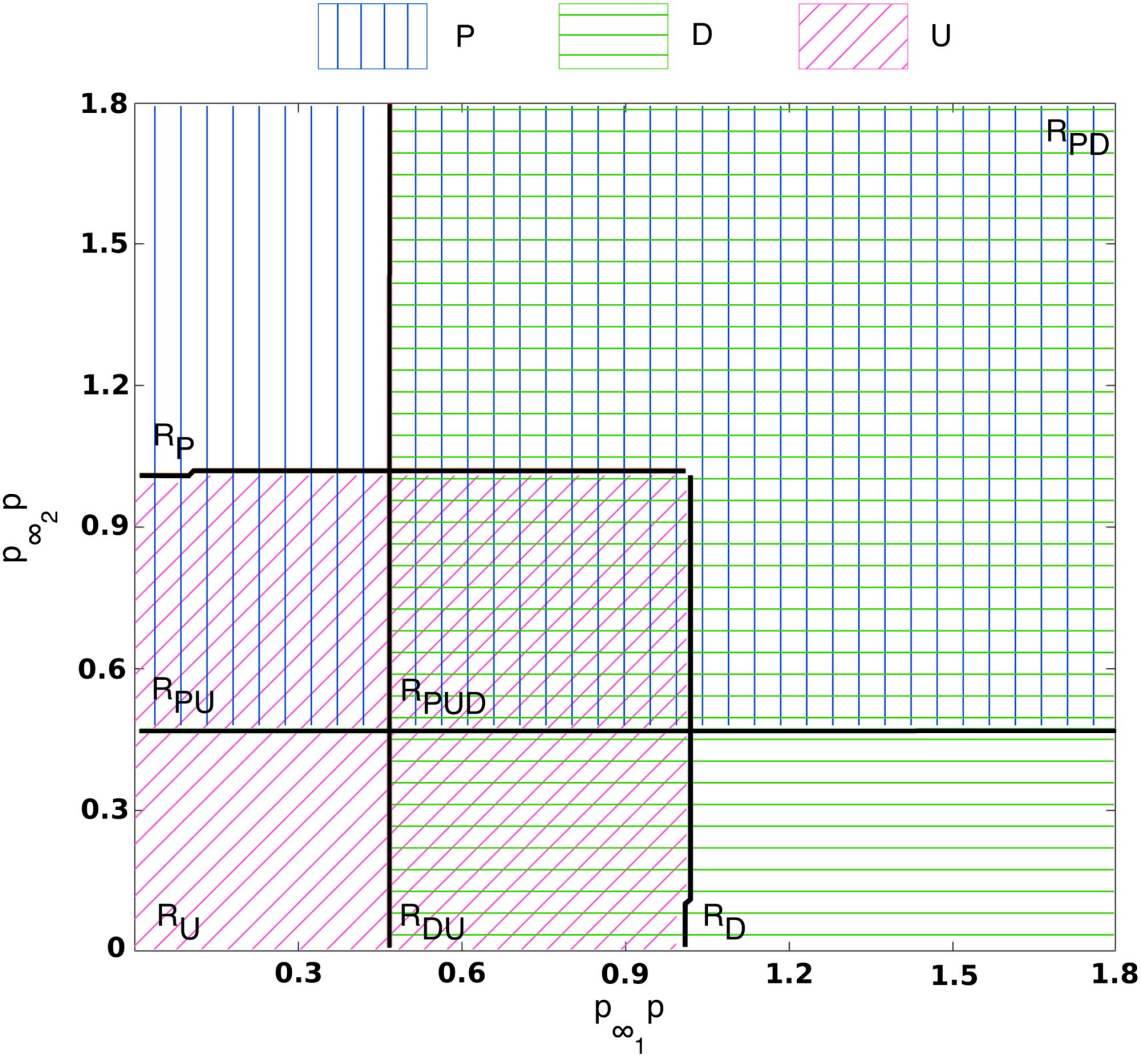
Phase diagram of the two-gene system, Eqs (5) and (6). Vertical blue (horizontal green) hatching denotes regions where the pluripotency (differentiated) state is stable. Diagonal pink hatching denotes regions where the undecided state is stable. Regions of the phase diagram where different hatchings overlap correspond to regions of bistability or tristability. In the labels in the plot, *P* stands for pluripotency, *D* stands for differentiation and *U* for undecided. This phase diagram was obtained using the methodology formulated in [57]. Parameter values: *ω*_11_ = *ω*_21_ = 4.0. Other parameter values as per S7 Table.

The multiscale analysis carried out in Section *Multiscale analysis of the GRN system: WKB approximation and multiscale optimal path theory* of the S1 Text shows that the parameters *p*, $p_{\infty_{1}}$ and $p_{\infty_{2}}$ are such that $p_{\infty_{1}}p=\frac{e_{1}}{E}$ and $p_{\infty_{2}}p=\frac{e_{2}}{E}$, where *e*_1_ and *e*_2_ are the number of binding sites in the promoter region of the differentiation and pluripotency gene, respectively, which are exposed to and available for binding by TFs. This implies that $p_{\infty_{1}}p$ and $p_{\infty_{2}}p$ can be directly related to ER. Since *E* is a constant denoting the average number of binding sites at the gene promoter region, a change in the parameter value of $p_{\infty_{i}}$ is due to a different value of the available number of binding sites, *e*_*i*_. Hence, $p_{\infty_{i}}p\to 0$ (i.e., few binding sites are available), *i* = 1, 2, corresponds to an epigenetically silenced gene, whereas $p_{\infty_{i}}p\geq O\left(1\right)$ (i.e., a large number of binding sites is available) associates with an epigenetically open gene. In this section, we study the phase space of the system when both $p_{\infty_{1}}p$ and $p_{\infty_{2}}p$ are varied. This allows us to understand how the behaviour of the GRN changes when its components are subject to ER. Our results are shown in Fig 3.

The system described by Eqs (5) and (6) exhibits three types of biologically relevant stable steady states, namely, the *pluripotency* steady state (PSS), the *differentitation* steady state (DSS), and the *undecided* steady state (USS). The PSS (DSS) corresponds to a steady state with high (low) levels of expression of the pluripotency gene and low (high) levels of expression of the differentiation gene, *q*_1_ ≪ 1 and $q_{2}=O\left(1\right)$ ($q_{1}=O\left(1\right)$ and *q*_2_ ≪ 1), and the USS is associated with a state such that both genes are expressed at low levels, i.e. *q*_1_ ≪ 1 and *q*_2_ ≪ 1. The existence of the latter state, the so-called “undecided”, is of particular interest because it closely relates to experimental results indicating that irreversible commitment to leave pluripotency and cell fate specification do not occur simultaneously [30, 59]. Therefore, such undecided state, which is characterised by low expression levels of pluripotency and differentiation genes, can be understood as a state where the cell has committed to leave pluripotency, indicated by the low expression levels of pluripotency related genes, but has not been committed yet to a particular differentiation fate, since differentiation-related genes are not up-regulated.

Different combinations of these states can coexist depending on the parameter values $p_{\infty_{i}}p$ (see Fig 3), revealing a complex phase space with seven different regions: monostability regions ($R_{U}$,$R_{P}$,$R_{D}$), bistability regions ($R_{PU}$,$R_{DU}$,$R_{PD}$), and a tristability region ($R_{PUD}$). The lines shown in Fig 3 correspond to the stability boundary of the different regimes. At such boundaries, saddle-node bifurcations occur, as illustrated in the example shown in Fig B (Section *Benchmark: stochastic model of a single self-activating gene* of the S1 Text). S3 Fig shows examples of trajectories illustrating the dynamics described by Eqs (5) and (6) for different values of the pair $\left(p_{\infty_{1}}p,p_{\infty_{2}}p\right)$ corresponding to the different regions shown in Fig 3. In particular, we show how the long term behaviour of different initial conditions differ as $\left(p_{\infty_{1}}p,p_{\infty_{2}}p\right)$ varies, so that different cell fates (co)exist associated with different levels of TF accessibility.

The phase space shown in Fig 3 illustrates the enormous relevance of ER, represented by the values of $p_{\infty_{i}}p$, which determine the pluripotent, differentiated or undecided cell fate. It is noteworthy that transitions between different cell fates can be achieved by simply altering the ER status, to which the GRN responds by jumping between different regions of the phase diagram (Fig 3). For example, by epigenetically silencing the differentiation gene (i.e., by decreasing $p_{\infty_{1}}p$), the GRN system could move from a differentiated state ($R_{D}$ region) to an undecided one ($R_{U}$ region). If this epigenetically silencing is accompanied by an increase in the number of binding sites available at the promoter region of the pluripotency gene (i.e., increase in $p_{\infty_{2}}p$), the GRN system can enter the $R_{P}$ region and reach a pluripotent cell fate. Indeed, there exist several possible transitions between cell fates by solely altering ER, thus highlighting its key role in switching fate. Its consequences for cell differentiation, cell reprogramming and cell locking is the focus of our study in the following sections.

### Co-factor heterogeneity gives rise to both pluripotency-locked and differentiation-primed states

In the previous section, we have analysed the landscape (phase space) provided by the dynamical system describing the GRN. We now proceed to study the effect of ER on the robustness of the different phases shown in Fig 3 (see also S3 Fig). ER is essential to the robustness of such phases and, consequently, to the stability of the associated cell fates. Stochastic transitions in bistable ER systems can induce (or facilitate) transitions between the GRN phases, which are associated with differentiation and reprogramming of cell fates. This phenomenon, so-called *epigenetic plasticity*, has been recently proposed as a major driver for disrupting cell-fate regulatory mechanisms in cancer and ageing [46]. We further focus on the role of heterogeneity within the ensemble of ER systems described in Section *Materials and methods* (see also [16]).

**Fig 4:**
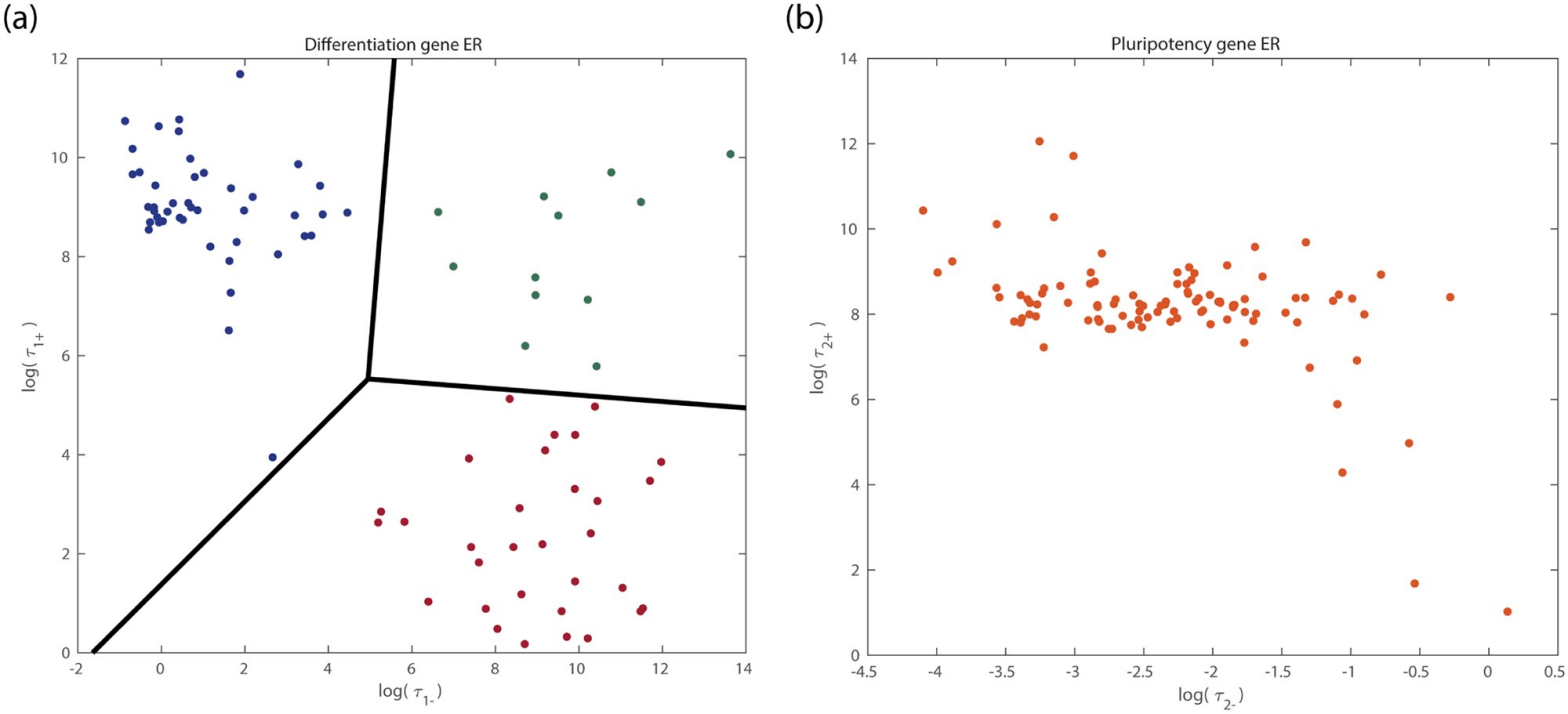
Scatter plots showing heterogeneity in the behaviour of bistable (a)differentiation ER systems (DERSs) and (b)pluripotency ER systems (PERSs). The vertical axis corresponds to the average opening time and the horizontal axis, to the average closing time. Each dot in plot (a) represents a DERS within the ensemble (see Section *ER-system ensemble generation and parameter sensitivity analysis*). We analyse a total of 90 DERS parameter sets and 100 PERSs. The red cluster includes 31 sets, the green cluster contains 13 sets, and the blue cluster has 46 sets. Different colours and black lines show the three clusters resulting from a *k*-means analysis discussed in Sections *Co-factor heterogeneity gives rise to both pluripotency-locked and differentiation-primed states* and *Analysis of ensemble heterogeneity*. Dots in plot (b) represent PERSs within the ensemble defined in Section *ER-system ensemble generation and parameter sensitivity analysis*.

In order to characterise robustness of the different ER systems within the ensemble, we have focused on the analysis of the average transition times between the *open* and *closed* ER states. We perform this analysis for each differentiation ER system (DERS) and each pluripotency ER system (PERS). We define an ER system to be *open* (*closed*) when the acetylation levels are over (under) 90% (10%). We denote by $\tau_{1_{+}}$ ($\tau_{1_{-}}$) the average transition time for a DERS to switch from closed to open (open to closed). Similarly, the quantities $\tau_{2_{+}}$ and $\tau_{2_{-}}$ are analogously defined for the PERSs. The results are shown in Fig 4(a) and 4(b), where we present scatter plots of the average transition times within the ensemble of DERSs (Fig 4(a)) and PERSs (Fig 4(b)). Each point corresponds to an ER system (i.e. a given parameter set) within our ensemble. By inspection, we observe that the heterogeneity exhibited by the differentiation ER systems, showing large degrees of heterogeneity in both $\tau_{1_{+}}$ and $\tau_{1_{-}}$ (Fig 4(a)), is greater than the one corresponding to the pluripotency ER systems (Fig 4(b)). In particular, the dispersion in $\tau_{2_{+}}$ is much smaller than in $\tau_{1_{+}}$, suggesting that DERSs have more variability in opening times than PERSs.

Heterogeneity in the differentiation ER systems exhibits an interesting pattern: DERSs organise themselves in three clusters obtained through *k*-means clustering, shown as blue, green and red dots in Fig 4(a). DERSs within the *blue* cluster are charaterised by long closed-to-open waiting times and short open-to-closed waiting times. DERSs belonging to the *red* cluster are the specular image of those within the blue cluster, i.e. they have short closed-to-open waiting times and long open-to-closed waiting times. Finally, DERSs in the *green* cluster are characterised by large values of both $\tau_{1_{+}}$ and $\tau_{1_{-}}$.

Insight into the stochastic dynamics, particularly regarding transitions between open and silenced ER states due to intrinsic noise, can be gained by analysing the corresponding optimal escape paths, φ_*_, which by means of the MAP theory provide the transition times (see Eq (1)). To illustrate this, four examples of such paths, which were computed according to the MAP theory (see Section *Transitions between ER states: minimum action path approach*), for two DERSs and two PERSs of the corresponding generated ensembles are shown in S4 Fig. A comparison between the value of the minimum action, S (see Eq (2)), associated with each of these systems, shows a tendency for DERSs to exhibit a larger degree of variability (see Table 1). Whilst the action value corresponding to the closed-to-open transition exhibits about a two-fold variability between PERSs, there is an over 8-fold increase when comparing the action values for this transition for DERSs. Similarly, when comparing the action S for the open-to-closed optimal paths, we observe that the variability associated with the DERSs is also larger than the one in PERSs. This property partly explains the difference between Fig 4(a) and 4(b) regarding DERSs and PERSs heterogeneity, respectively. A similar argument can be put forward to help us explain the heterogeneity within the DERS ensemble (Fig 4(a)). Blue cluster DERSs (illustrated by DERS2 in Table 1) exhibit optimal closed-to-open paths with larger value of the optimal action than that found in their red cluster (depicted by DERS1 in Table 1) counterparts (see S4(a) Fig). This property has the consequence that the closed-to-open waiting time, $\tau_{1_{+}}$, is longer for blue cluster DERSs.

**Table 1:** Minimum action values, S, corresponding to the optimal escape paths shown in S4 Fig (for details, see Section *Transitions between ER states: Minimum action path approach* and Section *Co-factor heterogeneity gives rise to both pluripotency-locked and differentiation-primed states* for details). Parameter values are given in S5 Table.

ER system	Open to closed	Closed to open
DERS1	0.05387	0.007012
DERS2	0.09947	0.05813
PERS1	0.01502	0.1836
PERS2	0.02043	0.07645

To quantify the effects of bistable ER on the landscape related to the gene regulatory system (see Fig 3), we proceed to estimate the probability, Q, that the combined activity of each pair of DERS and PERS within our ensemble produces a global epigenetic regulatory state compatible with differentiation. DERS-PERS pairs with high values of Q are associated with *differentiation-primed* states. By contrast, those DERS-PERS combinations with low Q are identified with *pluripotency-locked* states.

Since escape times from a stable attractor in a stochastic multistable system are exponentially distributed [44, 45], the PDFs for the escape times for both DERSs and PERSs are fully determined by the corresponding values of *τ*_1±_ and *τ*_2±_. We also assume that, for a given ER-GRN system, the DERS and the PERS evolve independently of each other.

We consider the PDF of the waiting time associated with a scenario of full remodelling of the epigenetic landscape, *τ*_*P*_. Such a scenario assumes that the system is initially in a pluripotency-locked ER state where the DERS is closed and the PERS is open. We denote such epigenetic state by *D*_−_ *P*_+_. For the system to make its transit into the differentiation-primed state *D*_+_ *P*_−_, corresponding to open DERS and closed PERS, there are two possible reprogramming routes: *D*_−_ *P*_+_ → *D*_−_ *P*_−_ → *D*_+_ *P*_−_ (route 1) and *D*_−_ *P*_+_ → *D*_+_ *P*_+_ → *D*_+_ *P*_−_ (route 2). Simultaneous switch of both ER systems is considered highly unlikely and therefore ignored. The PDF of the waiting time of the transition *D*_−_ *P*_+_ → *D*_+_ *P*_−_, denoted by *P*_+−,−+_(*τ*_*P*_), is given by:

$$\begin{array}{c} P_{+-,-+}\left(\tau_{P}\right)=Z^{-1}(P_{1}\left(\tau_{P}\right)+P_{2}\left(\tau_{P}\right)), \end{array}$$ (7)

where

$$\begin{array}{c} P_{1}\left(\tau \right)=\tau_{1-}^{-1}\tau_{2+}^{-1}e^{-\tau /\tau_{1-}}(\frac{e^{-\tau /\tau_{2+}}-e^{-\tau /\tau_{2-}}}{\tau_{2-}^{-1}-\tau_{2+}^{-1}}) \end{array}$$

$$\begin{array}{c} P_{2}\left(\tau \right)=\tau_{1-}^{-1}\tau_{2+}^{-1}e^{-\tau /\tau_{2+}}(\frac{e^{-\tau /\tau_{1+}}-e^{-\tau /\tau_{1-}}}{\tau_{1-}^{-1}-\tau_{1+}^{-1}}), \end{array}$$

and

$$\begin{array}{c} Z^{-1}=\frac{\left(\tau_{1-}+\tau_{2+}\right)(\left(\tau_{1-}^{-1}+\tau_{2-}^{-1}\right)\left(\tau_{1+}^{-1}+\tau_{2+}^{-1}\right))}{\tau_{2+}^{-1}+\tau_{1+}^{-1}+\tau_{2-}^{-1}+\tau_{1-}^{-1}}. \end{array}$$

*P*_1_(*τ*_*p*_) and *P*_2_(*τ*_*p*_) are the probabilities related to each of the landscape reprogramming routes. The probability that the ER landscape has undergone reprogramming from pluripotency-locked to differentiation-primed state within the time interval (0, *τ*_*P*_], Q, is thus given by:

$$\begin{array}{c} Q\equiv \int_{0}^{\tau_{P}}P_{+-,-+}\left(\tau \right)d\tau , \end{array}$$ (8)

where in our case, *τ*_*P*_ has been taken as the mean ensemble time for the differentiation ER systems (DERSs) to switch from the closed to the open state, *τ*_1+_. Furthermore, *τ*_1+_ exhibits a greater range of variability than the time for the pluripotency ER systems to switch from its open to its closed state, which is also a necessary condition for the epigenetic remodelling to take place.

We investigate the DERSs belonging to the different clusters of Fig 4(a) regarding their likelihood to produce pluripotency-locked epigenetic landscapes (results shown in S2 Fig). The analysis shows that when a DERS within the red cluster is paired with any PERS (S2(c) Fig), the resulting system corresponds to a differentiation-primed epigenetic landscape (Q=1). By contrast, when a PERS is paired with DERSs from the blue cluster (S2(a) Fig) and the green cluster (S2(b) Fig), both differentiation-primed (large Q) and pluripotency-locked (small Q) epigenetic landscapes are obtained. As discussed in the next section, the latter are more likely within the blue cluster.

### Analysis of ensemble heterogeneity

We now proceed to analyse the patterns observed in our ensemble of ER systems regarding both the differences between the three clusters observed in the ensemble of DERSs (Fig 4(a)) and the distinctive features that characterise pluripotency-locked DERS-PERS pairs. We followed the methodology put forward in [16], whereby statistics (in our case, cumulative distribution functions (CDFs)) of a subensemble of systems exhibiting a particular behaviour are analysed. We focused on the study of the CDFs of the kinetic parameters of the ER reactions, *c*_*ij*_ (see S3 Table), belonging to the DERSs/PERSs associated with the relevant behaviour aiming to characterise. In our case, such subensembles are either the different clusters, or they are determined by whether they exhibit low or high value of Q. By comparing such CDFs to either the general population (i.e. whole ensemble) or to different subensembles, we can detect statistically significant biases, which allows us to identify key parameters (and their biases) associated with the behaviour displayed by the subensemble under consideration.

#### Significant differences within the ensemble of DERSs

**Fig 5:**
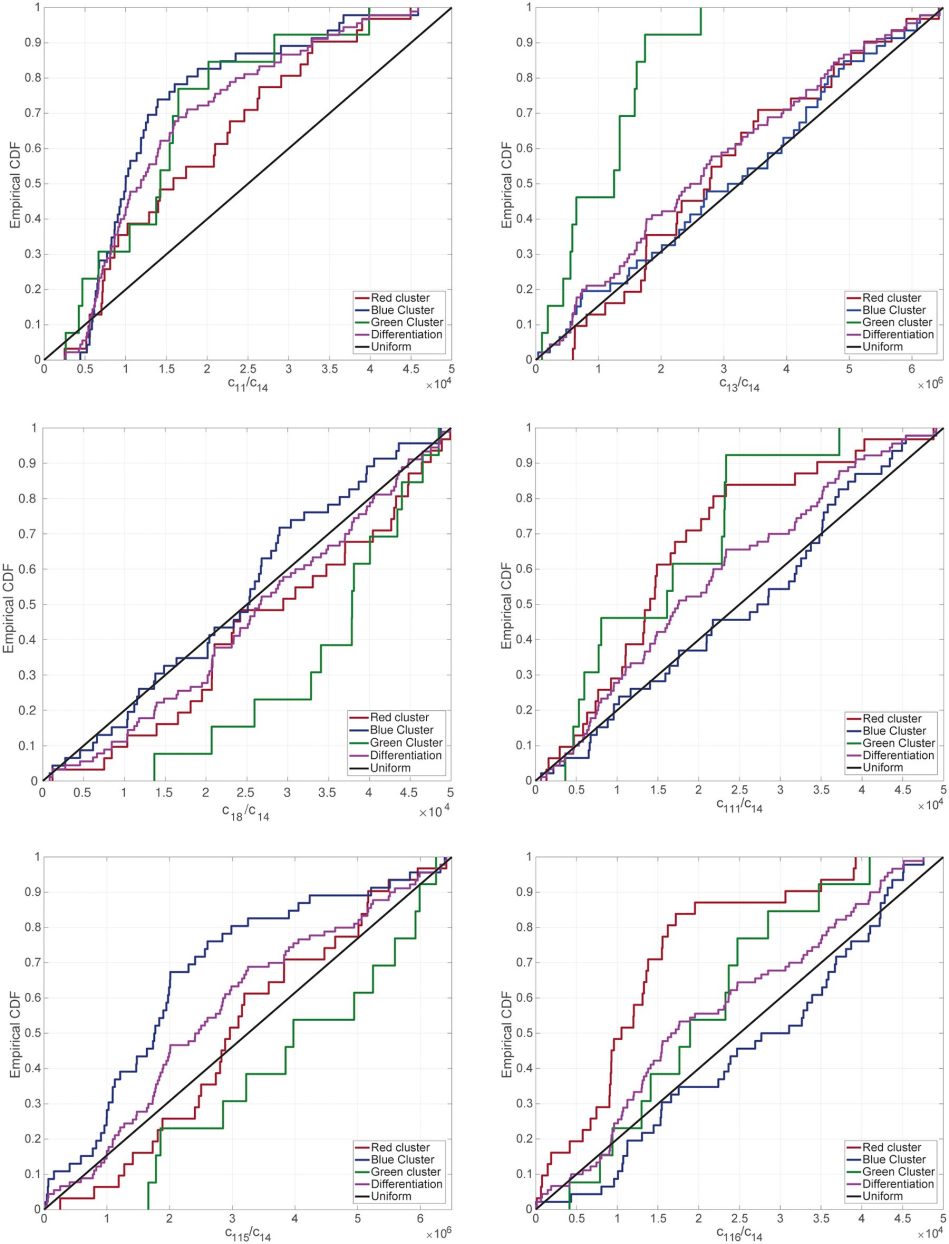
Empirical CDFs for the whole ensemble of DERS parameter sets (magenta lines) for those *c*_1*j*_ significantly different between clusters. This ensemble has been generated according to the methodology explained in Section *ER-system ensemble generation and parameter sensitivity analysis* (see also [16]). We also show the partial empirical CDFs corresponding to each of the clusters from Fig 4(a) (red, green, and blue lines). For reference, we also show the CDF for a uniform distribution (black line).

We start this analysis by studying the patterns emerging in the ensemble of DERSs, Fig 4(a). As discussed in the previous section, DERSs organise themselves in three clusters, which differ regarding their capability to trigger differentiation-primed epigenetic landscapes (see Fig 4(a) and S2 Fig). Our results are shown in Fig 5, where we depict the empirical CDFs for the relevant kinetic parameters of the ER reactions for the differentiation gene, $c_{1_{j}}$ (see S3 Table), i.e. those $c_{1_{j}}$ exhibiting statistically significant differences when comparing the CDFs of the clusters (red, green, blue) among them (see Fig 5). Each of these two-sample comparison is carried out by means of the Kolmogorov-Smirnov (KS) test. Statistically significant differences were found in the cases we comment below. The *p*-values are reported in Section *Analysis of ensemble heterogeneity: significant differences* of the S1 Text. The remaining CDFs for the ER reactions of the differentiation gene are given in S5 Fig.

*Red cluster versus blue cluster*. As discussed in the previous section, the differences between DERSs within the blue and red clusters are essential to ascertain the main features that distinguish differentiation-primed and pluripotency-locked systems. The bias detected within the red (blue) cluster in the corresponding CDFs (see Fig 5) is towards bigger (smaller) values for $c_{1_{1}}$ (unrecruited demethylation) and $c_{1_{15}}$ (unrecruited acetylation) and towards smaller (larger) values for $c_{1_{11}}$ (unrecruited deacetylation) and $c_{1_{16}}$ (recruited acetylation). The behaviour of $c_{1_{1}}$, $c_{1_{11}}$, and $c_{1_{15}}$ is straightforward to interpret. The trends observed in the data are consistent with the DERSs within red cluster being more prone to differentiation-primed ER landscapes, as they promote removal of negative (methylation) marks and addition of positive (acetylation) marks.

*Red cluster versus green cluster*. In this case, the bias detected within the red (green) cluster in the corresponding CDFs (see Fig 5) is to larger (smaller) values for $c_{1_{3}}$ (unrecruited demethylation) and to smaller (bigger) values for $c_{1_{16}}$ (recruited acetylation). The tendency in the data corresponding to $c_{1_{3}}$ is compatible with the features of the red cluster DERSs, as it involves an increase in the removal of negative marks.

*Blue cluster versus green cluster*. Fig 5 shows that DERSs within the green cluster have smaller values of $c_{1_{3}}$ (unrecruited demethylation) and larger values of $c_{1_{8}}$ (recruited methylation) than their blue cluster counterparts. Both of such effects stimulate addition of negative marks. However, DERSs in the green cluster also exhibit lower $c_{1_{11}}$ (unrecruited deacetylation) and bigger $c_{1_{15}}$ (unrecruited acetylation), which both encourage addition of positive marks. This can explain why the green cluster DERSs exhibit both long *τ*_1−_ and *τ*_1+_ (see Fig 4(a)).

#### Significant differences between differentiation-primed and pluripotency-locked ER landscapes

The quantity Q allows us to classify each pair DERS-PERS drawn from our ensemble regarding their degree of resilience to switch into a state prone to differentiation. If Q is larger than a threshold value T, the corresponding DERS-PERS pair is categorised as differentiation-primed. By contrast, when Q<T, the DERS-PERS pair is classified as pluripotency-locked.

We first proceed to compare within the whole population (without discriminating among clusters) those DERSs such that Q≥T (differentiation-primed ER landscapes) against those with Q<T (pluripotency-locked ER landscapes). We take T=0.7. The results are shown in S6 Fig. The CDFs of the parameters $c_{1_{1}}$ (unrecruited demethylation), $c_{1_{14}}$ (recruited deacetylation), and $c_{1_{15}}$ (unrecruited acetylation) are biased towards higher values for the subensemble associated with differentiation-primed ER landscapes (Q≥T). The requirement for Q to be Q≥T biases the CDF of $c_{1_{16}}$ (recruited acetylation) towards lower values than in the general population. The interpretation of the results regarding $c_{1_{1}}$ and $c_{1_{15}}$ is clear, since they encourage the removal of negative marks and the addition of positive marks, respectively, and thus promote expression of the differentiation gene. The CDFs of $c_{1_{14}}$ and $c_{1_{16}}$ corresponding to differentiation-primed ER landscapes are virtually identical to the CDFs associated with the general population (see S6 Fig). These features are therefore inherent in bistable behaviour (see Section *General description of the stochastic model of an epigenetically-regulated gene regulatory network*), rather than being specific to differentiation-primed DERSs.

**Fig 6:**
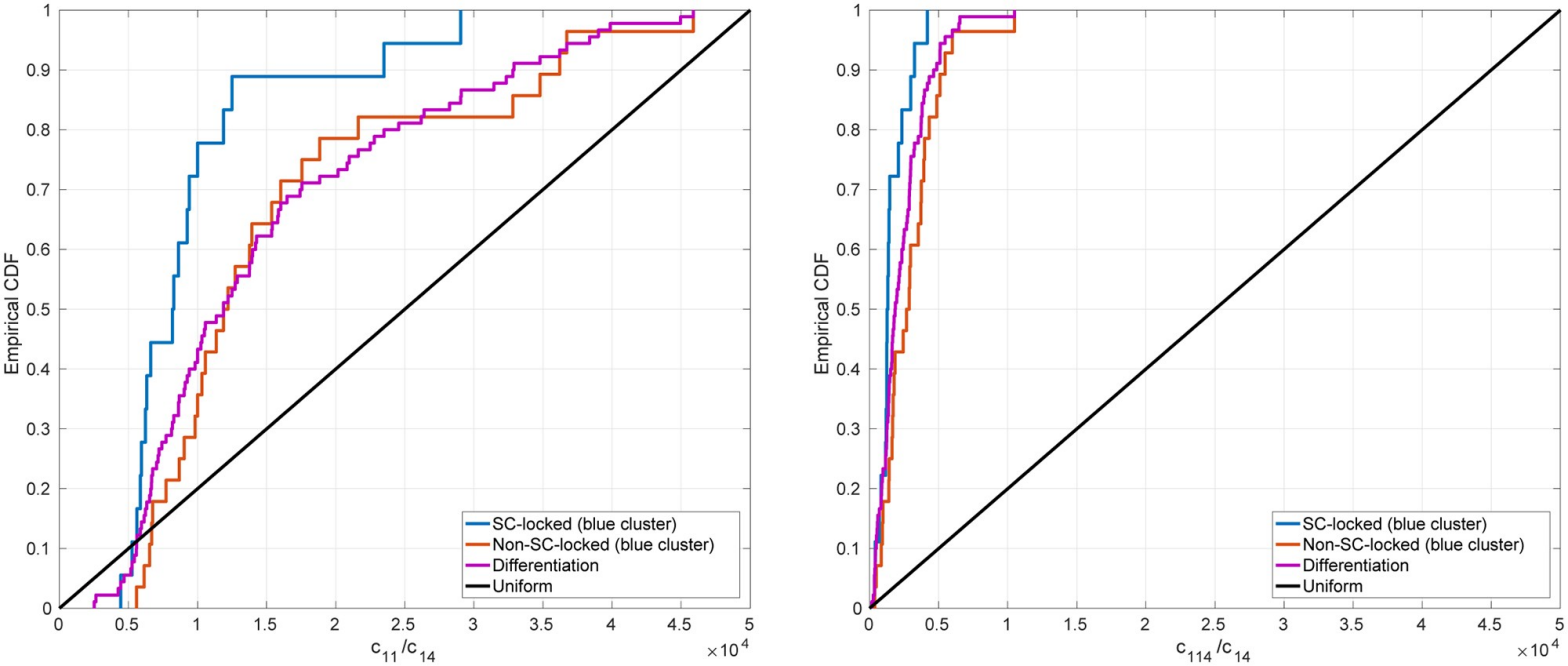
Empirical CDFs for the DERS parameter sets within the blue cluster for those *c*_*ij*_ significantly different. This ensemble has been generated according to the methodology explained in Section *ER-system ensemble generation and parameter sensitivity analysis* (see also [16]). The DERSs within the blue cluster have been divided into two subsets: those such that Q<T (SC-locked, blue lines) and those such that Q≥T (non-SC-locked, orange lines), with *T* = 0.7. For comparison, we plot the CDFs of the whole DERS ensemble (magenta lines), and, for guidance the CDF corresponding to a uniformly distributed random variable (black lines).

If we now restrict our analysis to those DERSs within the blue cluster (see Fig 6), we observe that the parameters whose CDFs differ significantly when splitted into differentiation-primed and pluripotency-locked are $c_{1_{1}}$ (unrecruited demethylation) and $c_{1_{14}}$ (recruited deacetylation). As in the analysis in the whole ensemble, only the result regarding $c_{1_{1}}$ is relevant for the analysis of the features yielding differentiation-primed ER landscapes. The remaining CDFs for those *c*_*ij*_ not exhibiting significant differences when comparing those DERSs within the blue cluster giving rise to differentiation-primed behaviour to those behaving like pluripotency-locked, are given in S7 Fig.

Regarding the PERSs, the results are less compelling. The results are shown in S8 Fig. Our analysis shows that significative differences can be found between the empirical distributions of three parameter values: $c_{1_{3}}$ (unrecruited demethylation), $c_{1_{8}}$ (recruited methylation), and $c_{1_{15}}$ (unrecruited acetylation). PERSs such that Q≥T exhibit larger values of all three parameters.

### Ensemble-based strategies for unlocking resilient pluripotency

**Fig 7:**
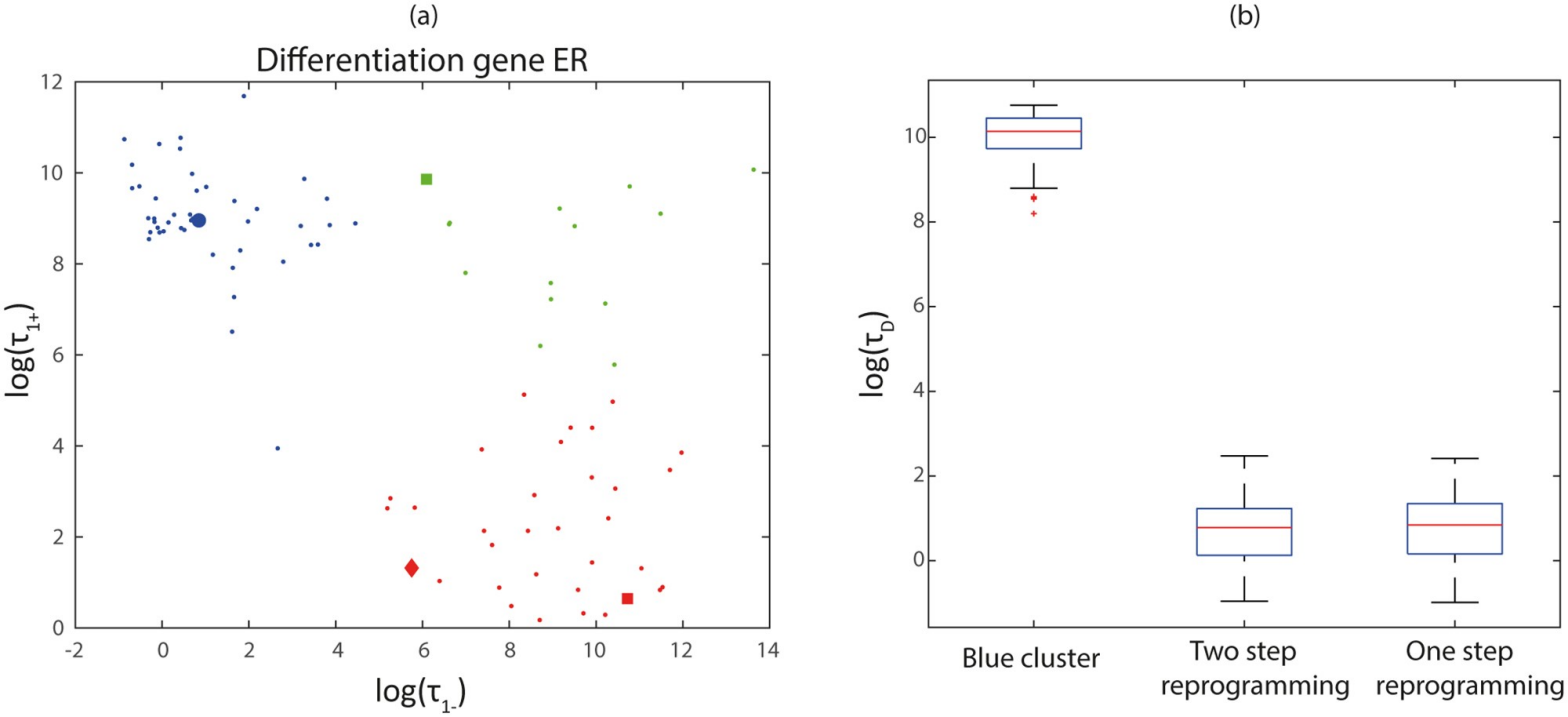
Effect of the different reprogramming strategies of blue cluster DERSs, as evaluated in terms of the statistics of the differentiation time (*τ*_*D*_). (a) Two step reprogramming is illustrated by the green square (first step), which finally becomes the red square (second step). One step reprogramming is depicted as the red diamond (see Section *Ensemble-based strategies for unlocking resilient pluripotency* for details). (b) Comparison of *τ*_*D*_ for the original DERS and the ones resulting from the reprogramming strategies. We consider a base-line scenario where the number of HMEs is exactly equal to average, i.e. *e*_*HDM*_ = *e*_*HDAC*_ = *Z*. We then compare the simulation results obtained for different scenarios regarding the different strategies to the base-line scenario. Parameter values: *Z* = 5 and *Y* = 15. Other parameter values given in S6 Table.

Our analysis has illustrated which are the key *c*_*ij*_ giving rise to either pluripotency-locked or differentiation-primed landscapes, immediately suggesting a number of strategies to unlock resilient pluripotency states with hindered differentiation. One of our main conclusions is that states of resilient pluripotency are mostly vinculated to DERS-PERS combinations such that the DERS belongs to either the blue or the green cluster, since they are the ones exhibiting pluripotency-locking (low Q). In view of this, a possible strategy in order to encourage differentiation-primed ER landscape consists in changing a selected combination of parameter values according to a rationale provided by the analysis carried out in the previous sections. Our results are shown in Fig 7.

One possible strategy consists in first transforming a blue cluster DERS into a green cluster one, and then completing the DERS *reprogramming* by transforming the resulting set into a red cluster DERS, where all DERSs exhibit differentiation-priming. A candidate strategy involves first changing a parameter whose CDF is significantly different when the blue cluster is compared with the green cluster. The second step is then to change a parameter that exhibits significant difference between the green and red cluster. Taking the results of the previous section into consideration, we consider the reduction of $c_{1_{11}}$ (unrecruited deacetylation) and the increase of $c_{1_{3}}$ (unrecruited demethylation). The result of this reprogramming strategy is shown in Fig 7(a), where we show that a blue cluster DERS is first transformed into a green cluster one (green square in Fig 7(a)), and then, finally, into a red cluster DERS (red square in Fig 7(a)). The initial blue cluster DERS has been chosen as the set with the largest value of $c_{1_{11}}$, which has been shown to be a significant difference when comparing the blue cluster to the red one, and the blue cluster to the green one, leading to the idea that this property is linked to the blue cluster (idea which is reinforced because $c_{1_{11}}$ is not significantly different when comparing the red and the green cluster).

The efficiency of such a strategy to unlock resilient pluripotency is shown in Fig 7(b) where we present statistics of the differentiation time, *τ*_*D*_, for the original blue cluster DERS and for the corresponding reprogrammed one (two step reprogramming, *red cluster-like*). These simulations have been done for the full ER-GRN, using the hybrid multiscale simulation algorithm described in S1 Appendix (and fully developed in Sections *Multiscale analysis of the GRN system: WKB approximation and multiscale optimal path theory* and *Stochastic model reduction method* of the S1 Text). The resulting differentiation times for the ER-GRN with reprogrammed ER landscape are orders of magnitude smaller than those with original ER-GRN within the blue cluster DERS, therefore confirming the success of our strategy.

An alternative strategy, that involves changing the value of one parameter only, consists in increasing the value of $c_{1_{3}}$ (unrecruited demethylation). Such a strategy is not obvious, since $c_{1_{3}}$ is not one of the parameters whose empirical CDF has significant differences when DERS in the red cluster are directly compared with those in the blue cluster. However, since the CDF of $c_{1_{3}}$ is significantly different when both the blue cluster and the red cluster are compared to the green cluster, it is conceivable that increasing $c_{1_{3}}$ without further intervention could reprogram blue cluster DERSs. The result of this reprogramming strategy is shown in Fig 7(a) (red diamond). Simulation results shown in Fig 7(b) (two step reprogramming) confirm the viability of this approach. In fact, based on the statistics of the differentiation time, both strategies are virtually indistinguishable.

### Loss of HDAC activity hinders differentiation in our ER-GRN model

Besides variability associated with cofactor heterogeneity, represented in the ensemble of *c*_*ij*_ values, our model allows us to address the issue of variability regarding HME activity. HMEs are needed for the acetylation and methylation epigenetic modifications to take place, and their activities are known to be affected by physiological and pathological processes, including ageing and cancer. Here, we analyse the impact of HDM and HDAC loss of activity on the dynamics of differentiation. In particular, we simulate differentiation in our ER-GRN model to obtain statistics of the differentiation time to assess the effect of loss of HME activity. The simulations shown in this section have all been carried out using the hybrid multiscale simulation algorithm described in the S1 Appendix.

In order to clarify the effect of loss of HME activity on the ER model, we first consider the phase diagram of its mean-field limit in different situations (see [16] for details). This phase diagram depicts the closed, bistable and open region for a given ER system, using HMEs activity as parameters (HDM in the *x*-axis, HDAC in the *y*-axis). The results are shown in S9 Fig. The surface occupied by the bistable region (shaded blue region in S9 Fig) is much larger in blue cluster than in red cluster DERSs, because of the displacement of the boundary separating the closed and bistable behaviour. By comparison, the bistability region of the PERSs is narrower than that of the DERSs (see S9(b) and S9(d) Fig). In particular the boundary that separates the bistable phase from the closed phase (area at the left of the blue shaded region) is displaced towards smaller HDM activity (i.e., to the left) in the DERSs phase diagrams.

This property suggests that a possible strategy to promote a differentiation-primed landscape (*D*_−_ *P*_+_ → *D*_+_ *P*_−_) would be to decrease HDM activity, as this would drive the PERSs into its closed phase whilst allowing the DERSs to remain within its bistability region. In order to assess this, we consider a base-line scenario where the number of HMEs is exactly equal to average, i.e. *e*_*HDM*_ = *e*_*HDAC*_ = *Z*. We then compare different scenarios regarding the abundance of HDM and HDAC to the base-line scenario.

**Fig 8:**
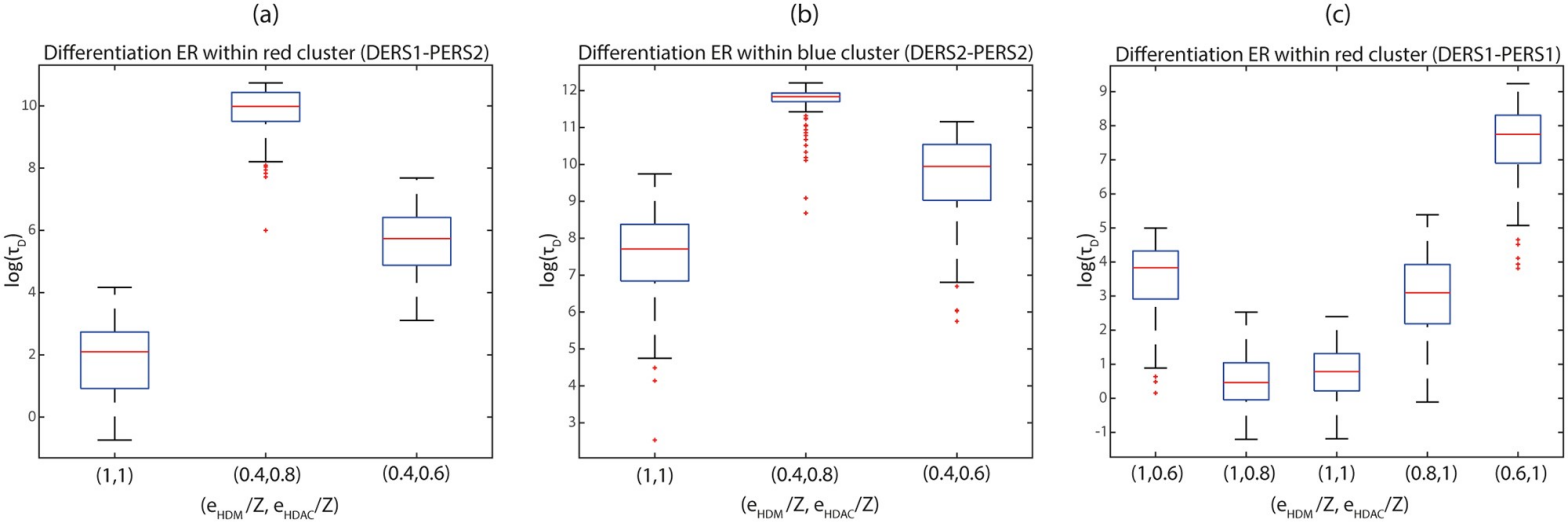
Plots showing the effect of the variation of HDM and HDAC on the statistics of the differentiation time (*τ*_*D*_). We consider a base-line scenario where the number of HMEs is exactly equal to average, i.e. *e*_*HDM*_ = *e*_*HDAC*_ = *Z*. We then compare the simulation results obtained for different scenarios regarding the abundance of HDM and HDAC to the base-line scenario, i.e. by changing the values of (*e*_*HDM*_, *e*_*HDAC*_). Parameter values: *Z* = 5 and *Y* = 15. Other parameter values given in S5 Table.

Contrary to what could be expected, simulation results show that the strategy of reducing HDM activity alone beyond the PERSs closing boundary further hinders differentiation. As can be seen in Fig 8(c), a decrease in HDM activity actually leads to longer differentiation time (see also [11]). Similarly, Fig 8(a) and 8(b), which show statistics of the differentiation time, reveal that a decrease in both HDM and HDAC activity also leads to an increment in differentiation times, that is, this strategy fails to decrease the differentiation time below the base-line scenario. In both cases, such hindrance of differentiation is the product of the increase in the opening times (*τ*_1+_) of the DERSs. This effect occurs because, as HDM and HDAC activity is reduced, the DERSs are driven towards their closed-bistability boundary. Close to such a region, the DERSs closed state becomes more stable and thus the corresponding *τ*_1+_ increases. By contrast, further reduction of HDAC activity moves the DERSs system closer to their bistable-open boundary, resulting in a reduction of the differentiation time. However, since the differentiation times remain above those corresponding to the base line HDM and HDAC activity scenario, we conclude that loss of both HDM and HDAC activity contributes towards hindering differentiation. Therefore, these results suggest that downregulation of HDM and HDAC activities, which has been observed in cancer and ageing, respectively, locks the ER landscapes in states more resilient to differentiation, as the differentiation time increases. This, in turn, is consistent with the theory that postulates that ageing and cancer may affect the ER control of cell fate, by locking cells into states disabled to differentiate and consequently, prone to malignant transformation.

## Discussion

Epigenomic remodelling in response to cellular reprogramming can be viewed as a paradigmatic strategy capable of erasing the hallmarks of ageing at the molecular and cellular level [1, 3, 4, 8]. However, undesirable *trade-off* constraining phenotypes such as impairment of tissue repair/wound-healing, tissue dysfunction due to loss of cell identity, and tumorigenesis could also occur if such epigenetic remodelling is not accompanied by an adequate self-repair of injury or disease [7]. In this regard, our study provides mathematical and computational answers to one of the ageing research field’s biggest challenges, namely, the understanding of how epigenetic heterogeneity could operate as the fundamental driver of the beneficial versus deleterious effects of cellular reprogramming.

We are rapidly amassing evidence that, beyond *bona fide* genetic alterations, non-genetic stimuli such as inflammation, hypoxia, cell stress, and developmental and metabolic cues, can promote overly restrictive epigenetic states—capable of preventing the induction of tumour suppression programmes or blocking normal differentiation—or overly plastic epigenetic states—capable of stochastically activating oncogenic programmes and non-physiological cell fate transitions including those leading to the acquisition of stem cell-like states [16, 46]. Indeed, resilience and plasticity begin to be considered as the fundamental epigenetic dimensions that ultimately dictate the capacity of cells, tissues, and organs to undergo successful repair, degeneration, or malignization via phenotypic variation. In our model, the robustness or resilience of the cell phenotype attractors throughout the epigenetic landscape was determined by ER; then, a framework for the generation of the ensemble of ER systems allowed an ulterior analysis, whereas a multiscale asymptotic analysis-based method for model reduction enabled us to formulate an efficient numerical scheme to study the behaviour of the stochastic ER-GRN system. Our approximation, which is closely related to the notion of neutral networks formulated to analyse systems with genotype-phenotype maps [60–62], is applicable to broader scenarios because, by reducing a rather complex stochastic system into a hybrid, piece-wise deterministic Markov one, it is capable of providing an efficient and scalable, hybrid numerical method able to simulate more complex ER-GRN systems.

In order to determine the key mechanisms underlying epigenetic plasticity and its connections with aberrant stem cell-like locked states, we have considered a gene network model of two mutually-inhibiting genes regulating the phenotypic switch between differentiated and pluripotent states. Each gene within this regulatory system was acted upon by ER to restrict/enable its expression capability. Although it might be argued that such a system is too simplistic to describe realistic frameworks, one should acknowledge that mutual inhibition between two key transcription factors has been shown to control binary cell fate decisions in a number of biologically relevant situations [30, 63]. In addition, this system serves as a general tool to understand generic features of the role of multistability in more complex cell fate decision systems. Specific examples include lateral stabilization during early patterning in the pancreas (Ngn3–Ptf1a) [64], promotion of differentiation to trophectoderm in mammalian angiogenesis (Cdx2–Oct3/4) [65], cellular reprogramming (Oct4–Sox2) [11], and haematopoiesis (GATA1–PU.1) [66]. Furthermore, our approach might serve as a general tool that can be applied in a straightforward manner to adequately evaluate the epigenetic-regulatory features involved in the multistability of larger, more complex cell fate decision systems. In this regard, it should be noted that the robustness of the open/closed epigenetic states was assessed in terms of the average transition times and that the structure of DERSs/PERSs clusters was independent of the GRN dimensionality. Therefore, robustness analyses of more complex ER systems would be carried out by merely sampling DERSs/PERSs parameters from the generated ensemble. Nonetheless, even in our relatively simple case of a gene regulatory circuit involving solely two genes, the behaviour of the mean-field limit of the GRN exhibited a complex space with a tristability regime, which included not only the expected stem-locked and differentiated steady-sates but also the so-called indecision state. From a developmental perspective, the latter state could serve the purpose of priming cells for differentiation. Perhaps more importantly, the transitions between the different phases could be triggered by changes related to ER (i.e., cofactors of chromatin-modifying enzymes), which thereby operate as *bona fide* molecular bridges that directly connect epigenetic and phenotypic plasticity via translation of changes in ER states into variations of GRN states.

**Fig 9:**
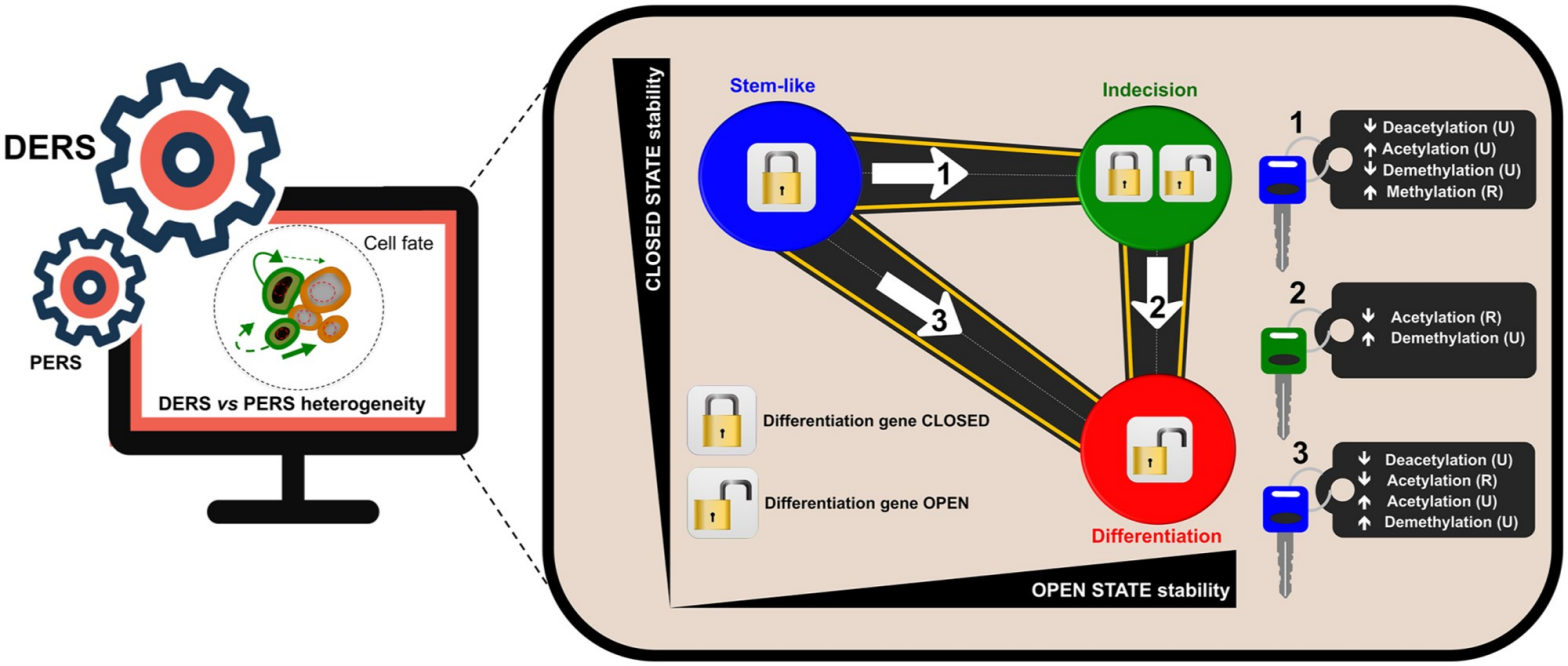
Strategies to unlock pluripotent stem-like states in ageing and cancer. Epigenetic regulation heterogeneity of differentiation genes (DERSs), but not that of pluripotency genes (PERSs), was predominantly in charge of the entry and exit decisions of the pluripotent stem-like states (blue). The application of the hybrid numerical method validated the likelihood of epigenetic heterogeneity-based strategies capable of unlocking and directing the transit from differentiation-refractory to differentiation-primed (red) epistates via kinetics changes in epigenetic factors. (Note: The epigenetic parameters regulating the entry into robust epi-states throughout the entire ER-GRN system revealed a regime of tristability in which pluripotent stem-like (blue) and differentiated (red) steady-states coexisted with a third indecisive (green) state). (R: Recruited; U: Unrecruited).

Earlier studies provided evidence that the *de novo* reprogramming potential is higher within select subpopulations of cells and that such pre-existing epigenetic heterogeneity can be tuned to make cells more responsive to reprogramming stimuli [16, 40]. Along this line, intra- and inter-individual variability driven by the local interpretation of metabolic, epigenetic, and inflammatory regulators might not only reflect the occurrence of different ageing trajectories in different tissue cell subpopulations but might determine also the *de novo* responsiveness to therapeutic strategies aimed to remodel the organismal self-repair capacity for resistance to damage, stress, and disease [16, 67, 68]. In this regard, by uncovering the regulatory details of the phenotypic robustness of stem-like epi-states, we have been able to mathematically capture how epigenetic heterogeneity governs the routes and kinetics to entry and exit from unrestrained epigenetic plastic states. Thus, a sub-ensemble of ER systems with higher reprogramming potential was found to pre-exist within the ensemble of ER systems compatible with a terminally differentiated cell state; moreover, such a sub-ensemble could be harnessed to fine-tune the cellular response to reprogramming-to-stemness stimuli by solely targeting chromatin-modifying enzymes such as HDMs and HDACs, thus confirming and extending earlier experimental approaches [69]. It is reasonable to propose that epigenetic heterogeneity is a/the central regulator through which epigenetic plasticity allows cells to stochastically activate alternative regulatory programs and undergo distinct cell fate transitions. As such, epigenetic heterogeneity is largely responsible for the mechanistic dynamics determining the phenotypic robustness of cell fate reprogramming (see Fig 9).

A disruption of the homeostatic resilience of chromatin, causing it to become aberrantly restricted or permissive, has the potential to give rise to each classic cancer hallmark [46]. Intriguingly, a similar disruption of the entry-exit paths and kinetics of the endogenous injury-repair mechanisms appears to be also the convergent *trade-off* of a variety of strategies (e.g., metabolic manipulation, ablation of senescent cells, and cellular reprogramming) beginning to be recognised as valuable interventions directed against the ageing hallmarks [67]. Our current approach reconciles such apparently counterintuitive scenario by illuminating the occurrence of tunable switches in terms of epigenetic cofactor levels, which are capable of modifying the nature and direction of cell fate reprogramming. On the one hand, our mathematical deconstruction of epigenetic plasticity reveals that epigenetic heterogeneity may underlie the predisposition of cell populations to pathological reprogramming processes that cause a permanent, locked stem-like state disabled for reparative differentiation and prone to malignant transformation. On the other hand, we have computationally validated the likelihood of unlocking aberrant stem-like states disabled for reparative differentiation and drive them to a correct repair function by manipulating solely the intensity and direction of such epigenetic control switches. Therefore, we now propose that an ideal ageing-/cancer-targeted therapeutic approach must be able to correct chronic epigenetic plasticity of damaged/diseased tissues, but additionally, to “unlock” stem cell-like states to drive tissue regeneration, thereby preventing the occurrence of the abovementioned constraining phenotypes.

In summary, upon unearthing key regulatory dimensions of epigenetic plasticity in an unbiased manner, we here offer a conceptual and methodological re-orientation of how therapeutically approach pathological cellular reprogramming. As we enter a new era of therapeutic approaches to target ageing *per se* [67], our stochastic biomathematical modelling and computational simulation strategy might be incorporated as a valuable tool for assessing the benefit/risk ratio of new therapeutic approaches aimed to target and correct the ageing-/cancer-related perturbations of the epigenome.

## Supporting information

S1 Text Supplemental material. (PDF)

S1 Appendix Numerical method. (PDF)

S1 Fig Comparison of raw data and simulated data for the ER system. (TIF)

S2 FigDifferentiation probability Q within the DERSs and PERSs corresponding to the three clusters.(TIF)

S3 Fig Dynamics of the different regions shown in Fig 3. (TIF)

S4 Fig Optimal paths for DERS1, DERS2, PERS1 and PERS2. (TIF)

S5 Fig Empirical CDFs for those *c*_*ij*_ of the DERSs where significant differences between the clusters have not been found. (TIF)

S6 FigEmpirical CDFs for the (whole) ensemble of DERS parameter sets classified according to Q.(TIF)

S7 Fig Empirical CDFs for those *c*_*ij*_ of DERSs within the blue cluster where significant differences between differentiation-primed and pluripotency-locked behaviours have not been found. (TIF)

S8 FigEmpirical CDFs for the (whole) ensemble of PERS parameter sets classified according to Q.(TIF)

S9 Fig Phase diagrams depending on HME activity for DERS1, DERS2, PERS1 and PERS2. (TIF)

S1 Table General parameters involved in our ER-GRN model formulation. (PDF)

S2 Table Variables, rescaled variables and transition rates associated with the stochastic dynamics of the GRN submodel. (PDF)

S3 Table Variables, rescaled variables and transition rates associated with the stochastic dynamics of the ER submodel. (PDF)

S4 Table Reference parameter values used to generate the ensemble of DERSs and PERSs. (PDF)

S5 Table Parameter values for DERS1, DERS2, PERS1, PERS2. (PDF)

S6 Table Parameter values for the pluripotency-locked DERS being unlocked by the ER strategy. (PDF)

S7 Table Parameter values for the two-gene regulatory network. (PDF)
