# Supplementary material for: A multiscale model of epigenetic heterogeneity-driven cell fate decision-making
Source: PLoS Comput Biol. 2019 Apr 30;15(4):e1006592. doi: 10.1371/journal.pcbi.1006592 (PMC6510448; doi:10.1371/journal.pcbi.1006592)
Supplement: S1 Text — (PDF) [file pcbi.1006592.s001.pdf]

Supplemental Information

A multiscale model of epigenetic heterogeneity-driven cell fate decision-making

Núria Folguera-Blasco[1,2 *], Rubén Pérez-Carrasco[3], Elisabet Cuyàs[4,5], Javier A. Menendez[4,5], and Tomás Alarcón[6,1,2,7]

[1]Centre de Recerca Matemàtica. Edifici C, Campus de Bellaterra, 08193 Bellaterra (Barcelona), Spain
[2]Departament de Matemàtiques, Universitat Atònoma de Barcelona, 08193 Bellaterra (Barcelona), Spain
[3]Department of Mathematics, University College London, Gower Street, London WC1E 6BT, UK
[4]ProCURE (Program Against Cancer Therapeutic Resistance), Metabolism and Cancer Group, Catalan Institute of Oncology, Girona, Spain
[5]Girona Biomedical Research Institute (IDIBGI), Girona, Spain
[6]ICREA, Pg. Lluís Companys 23, 08010 Barcelona, Spain
[7]Barcelona Graduate School of Mathematics (BGSMath), Barcelona, Spain
[*]Correspondence: nuria.folguerablasco@crick.ac.uk, Current Address: The Francis Crick Institute, London, United Kingdom.

SUMMARY

This online supplement contain the technical details involved in the formulation and multiscale analysis of the ER-GRN system introduced in the main text, as well as additional information and results. Specifically:

1. Section *Stochastic model of epigenetic regulation* describes in detail the model assumptions leading to the stochastic ER model as per S3 Table of the main text.

2. Section *Multiscale analysis of the GRN system: WKB approximation and multiscale optimal path theory* is devoted to presenting the multiscale WKB analysis of the GRN part of the model with the aim of:

   - Deriving the quasi-steady state approximation (QSSA) for the probability density functions (PDFs) of the fast stochastic variables. Such QSSA PDFs are essential to the results of Section *Coupling the ER-GRN models: Multiscale analysis and model reduction* of the main text.
   - Deriving the (outer) optimal path equations for the gene regulatory system analysed in the main text (see Section *Multi-scale optimal path theory: estimation of the GRN relaxation time upon epigenetic switch*).
   - Deriving the associated estimate of the relaxation time of the GRN system upon epigenetic-regulatory switch (see Section *Multi-scale optimal path theory: estimation of the GRN relaxation time upon epigenetic switch*).

3. Section *Stochastic model reduction method* presents the details of the stochastic model reduction method used in the main text, which is based on the existence of multiple scales.

4. Section *Consistency with the stochastic model reduction method*verifies the consistency between the WKB analysis and the model reduction method. More precisely, in Sections *GRN model* and *Quasi-steady state distribution of the enzyme/complex sub-system (ER)* we derive the quasi-steady state probability density functions of the fast variables of the ER-GRN stochastic model.

5. Section *Benchmark: Stochastic model of a single self-activating gene* contains a comparison between our asymptotic results (both WKB and stochastic model reduction) for the benchmark case of a self-activating one-gene regulatory system.

6. In Section *Summary of the minimum action path theory and numerical method* we briefly present the details of the numerical method for action minimisation used to implement the Minimum Action Path (MAP).

7. In Section *Analysis of ensemble heterogeneity: significant differences* we present the p-values corresponding to the Kolmogorov-Smirnov tests performed in Section *Analysis of ensemble heterogeneity* of the main text.

STOCHASTIC MODEL OF EPIGENETIC REGULATION

In this section we present a review of the formulation of the stochastic model of epigenetic regulation proposed by [1]. We formulate a generalisation of the stochastic model for epigenetic regulation proposed by Dodd et al. [2] and Menéndez et al. [3]. The model originally formulated in [2] considers nucleosome modification as the basic mechanism for epigenetic regulation. Nucleosomes are assumed to be in one of three states, methylated (M), unmodified (U), or acetylated (A), and the dynamics of the model is given in terms of the transition rates between these three states.

Dodd et al. [2] consider that direct transitions between M and A are very unlikely. Instead, they assume that transitions occur in a linear sequence given by:

$$M \rightleftharpoons U \rightleftharpoons A,$$

i.e. they assume that methylated nucleosomes can only undergo loss of the corresponding methyl group to enter the unmodified state which can then, by means of the intervention of the corresponding histone-modification enzyme, acquire an acetyl group, and vice versa. They further put forward the hypothesis that such nucleosome modifications are of two types, namely, recruited and unrecruited:

- Recruited modification refers to a positive feedback mechanism by which change in the modification status of a nucleosome is facilitated by the presence of other modified nucleosomes (i.e. by the presence of other methylated or acetylated nucleosomes). Mathematically, this is expressed through a non-linear dependence on the number of M-nucleosomes and A-nucleosomes of the corresponding transition rates (see S3 Table of the main text).
- Unrecruited modification refers to nucleosome modifications whose probability is independent of the modification status of the other nucleosomes.

We consider two histone modifying enzymes: histone demethylase (HDM) and histone deacetylase (HDAC). More specifically, the reactions involved in our model are:

1. HDM-mediated demethylation:

$$M + HDM \leftrightarrows C_M \rightarrow U + HDM$$

   where C_M denotes the intermediate HDM enzymatic-substrate complex. HDM-mediated demethylation can be both unrecruited, where the rates associated with each reaction are constant (see S3 Table of the main text, reactions 1 to 3), and recruited, where all the associated rates are taken to be proportional to the number of A-nucleosomes (see S3 Table of the main text, reactions 4 to 6).

2. Methylation:

$$U \rightarrow M$$

   Methylation can also be unrecruited (S3 Table of the main text, reaction 7) or recruited, in which case the associated rate is proportional to the number of methylated nucleosomes (S3 Table of the main text, reaction 8)

3. HDAC-mediated deacetylation:

$$A + HDAC \leftrightarrows C_A \rightarrow U + HDAC$$

   where C_M denotes the intermediate HDAC enzymatic-substrate complex. HDAC-mediated deacetylation can be both unrecruited (see S3 Table of the main text, reactions 9 to 11), or recruited, where all the associated rates are proportional to the number of M-nucleosomes (see S3 Table of the main text, reactions 12 to 14).

4. Acetylation:

$$U \rightarrow A$$

   As with the previous processes, acetylation can also be unrecruited (S3 Table of the main text, reaction 15) or recruited, in which case the transition rate is proportional to the number of acetylated nucleosomes (S3 Table of the main text, reaction 16)

We consider the scenario where both histone hypomethylation and hyperacetylation allow genes to be expressed, insofar the associated transcription factors are present [4]. On the contrary, we associate histone hypermethylation and hypoacetylation to silent states where genes are not expressed even in the presence of the appropriate transcription factors. In the main text, we focus on the conditions for bistability to arise and the robustness of the associated *open* and *silened* states particularly in connection with the abundance or activity of HDMs and HDACs.

The transition rates (S3 Table of the main text) related to each of these reactions are modelled using the law of mass action [5]. This model is an extension of the one formulated by Menéndez et al. [3], where we explicitly account for the enzymatic activity of histone demethylases (HDMs) and histone deacetylases (HDACs). The model described by the transition rates from S3 Table of the main text has been analysed in detail in our previous work [1].

MULTISCALE ANALYSIS OF THE GRN SYSTEM: WKB APPROXIMATION AND MULTISCALE OPTIMAL PATH THEORY

In this section we provide a detailed derivation of the multiscale analysis of the gene regulatory (sub)system. In particular, we derive the quasi-steady multinomial probability distribution describing the QSSA of the occupancy of binding sites within the promoter region of the epigenetically open genes. Our current treatment considers an extension of the multiple scale WKB method that was first introduced in [6, 7]. We will show that under suitable conditions on separation of the different characteristic scales of the system (which will be precisely defined below), the WKB approximation of the outer solution collapses onto a trivial WKB mode. By contrast, within the corresponding inner regime, the solution is given by a non-trivial (the so-called fast) WKB mode, which turns out to take the form of a multinomial distribution.

Before proceeding further, it is worth noting that we focus our analysis on the GRN subsystem alone for clarity. The same analysis can be performed with the ER part of the whole system. In the remaiming of this manuscript, N_G will denote the total number of genes included in the GRN and the vector $\mathbf{X} = (X_1, \dots, X_{N_G}, X_{11}, \dots, X_{N_G N_G}$ contains the number of transcription factor monomers X_i and the number of sites of the promoter region i bound to a dimer of type j.

General setting: WKB and multiscale optimal path theory

The WKB approximation and the (closely related) optimal path theory are variants of large deviation theory which has been used to study mean first passage time problems associated with the escape from metastable states in a wide variety of contexts, including multistable systems [8, 9], extinctions in population-dynamical models [10], hybrid stochastic systems [11], gene regulatory systems [12, 13], and small stochastic systems [14]. We have recently extended this methodology to account for, and take advantage of, separation of time scales. In this section we give a brief summary of our methodology. For a full account of the technicalities we refer the reader to our previous work [6, 7].

An alternative formulation to analyse the dynamics of continuous-time Markov processes on a discrete space of states is to derive a partial differential equation for the generating function, $G(p_1, \dots, p_{N_G}, p_{11}, \dots, p_{N_G N_G}, t)$:

$$G(p_1, \dots, p_{N_G}, p_{11}, \dots, p_{N_G N_G}, t) = \sum_{\mathbf{X}} \left(\prod_{i=1}^{N_G} p_i^{X_i} \right) \left(\prod_{i,j=1}^{N_G} p_{ij}^{X_{ij}} \right) P(X_1, \dots, X_{N_G}, X_{11}, \dots, X_{N_G N_G}, t) \tag{S.1}$$

where $P(X_1, \dots, X_{N_G N_G}, t)$ is the solution of the Master Equation. The probability density generating function (PDGF) satisfies a partial differential equation (PDE) which can be derived from the corresponding Master Equation. This PDE is the basic element of the so-called momentum representation of the Master Equation [15–19]:

$$\frac{\partial G}{\partial t} = H_k \left(p_1, \dots, p_{N_G}, p_{11}, \dots, p_{N_G N_G}, \partial_{p_1}, \dots, \partial_{p_{N_G}}, \partial_{p_{11}}, \dots, \partial_{p_{N_G N_G}} \right) G(p_1, \dots, p_{N_G}, p_{11}, \dots, p_{N_G N_G}, t) \tag{S.2}$$

This PDE, or, equivalently, the operator H_k, is determined by the reaction rates shown in Table 3 of the main text: it is obtained by multiplying both sides of the corresponding Master Equation by $\left(\prod_{i=1}^{N_G} p_i^{X_i} \right) \left(\prod_{i,j=1}^{N_G} p_{ij}^{X_{ij}} \right)$ and summing up over all the possible values of $\mathbf{X}$. Furthermore, the solution of Eq.(S.2) must satisfy the normalisation condition $G(p_1 = 1, \dots, p_{N_G} = 1, p_{11} = 1, \dots, p_{N_G N_G} = 1, t) = 1$ for all t.

Eq.(S.2) allows us to define a Hamiltonian, $H_k(p, Q)$, where the position operators in the momentum representation have been defined as $Q_i \equiv \partial_{p_i}$ with the commutation relation $[Q_i, p_j] = S\delta_{i,j}$, where each pair (p_i, Q_i) is the set of generalised coordinates associated with the random variable X_i. The quantity S is the characteristic scale corresponding to the random variable X_i. Similarly, $Q_{ij} \equiv \partial_{p_{ij}}$ with $[Q_{ij}, p_{kl}] = E\delta_{i,k}\delta_{j,l}$. Each pair (p_{ij}, Q_{ij}) is associated with the random variable X_{ij}, and the quantity E is the characteristic scale associated with X_{ij} [7].

In order to proceed with our multiple time scale analysis, we assume, as per the Briggs-Haldane treatment of the Michaelis-Menten model for enzyme kinetics [20, 21], that the species involved in the system under scrutiny are divided into two groups according to their characteristic scales. More specifically, we have a subset of chemical species whose numbers, X_i, scale as $X_i = Sx_i$, where $x_i = O(1)$, whilst the remaining species are such that their numbers, X_{ij}, scale as $X_{ij} = Ex_{ij}$, where $x_{ij} = O(1)$. Key to our approach is the fact that S and E must be such that $\varepsilon = \frac{E}{S} \ll 1$. We further assume that the generalised coordinates, Q_i, scale in the same fashion as the corresponding variables X_i, i.e. $Q_i = Sq_i$, where $q_i = O(1)$. We refer to the variables belonging to this subset as *slow variables*. Similarly, $Q_{ij} = Eq_{ij}$, where $q_{ij} = O(1)$, which are referred to as *fast variables*. Moreover, we assume that the moment coordinates, p_i and p_{ij}, are all independent of S and E, and therefore remain invariant under rescaling.

Under this scaling for the generalised coordinates, we define the following scale transformation for the Hamiltonian in Eq. (S.2):

$$H_k(p,Q) = k_J S^n E^l H_\omega(p,q) \tag{S.3}$$

where J identifies the reaction with the largest order among all the reactions that compose the dynamics and k_J is the corresponding rate constant. The exponents n and l correspond to the number of slow and fast variables involved in the transition rate W_J, respectively. The last step is to rescale the time variable so that a dimensionless variable, τ, is defined such that:

$$\tau = k_J S^{n-1} E^l t \tag{S.4}$$

It is now a trivial exercise to check that, upon rescaling, Eq. (S.2) reads:

$$\frac{\partial G}{\partial \tau} = SH_\omega\left(p_1,\ldots,p_{N_G},p_{11},\ldots,p_{N_G N_G},\partial_{p_1},\ldots,\partial_{p_{N_G}},\partial_{p_{11}},\ldots,\partial_{p_{N_G N_G}}\right) G(p_1,\ldots,p_{N_G},p_{11},\ldots,p_{N_G N_G},\tau) \tag{S.5}$$

Closed, analytic solutions are rarely available for Eq. S.5. Nevertheless, the PDE for the generating function admits a perturbative solution, which is commonly obtained by means of the WKB method [10, 18]. From a formal point of view, Eq. (S.5) is a Schrödinger-like equation and, therefore, there is a plethora of methods at our disposal in order to analyse it. In particular, when the fluctuations are (assumed to be) small, it is common to resort to WKB methods [22–24], which consists on proposing the following *Ansatz* for the solution of Eq. (S.5):

$$G(p,\tau) = e^{S\mathscr{A}(p,\tau)},$$

where $\mathscr{A}(p,\tau)$, the so-called action, satisfies the Hamilton-Jacobi equation associated with the Hamiltonian $H_\omega(p,q)$:

$$\frac{\partial \mathscr{A}}{\partial \tau} = H_\omega\left(p, \frac{\partial \mathscr{A}}{\partial p}\right). \tag{S.6}$$

The solution to the above Hamilton-Jacobi equation can be given in terms of the solution of the associated Hamilton equations:

$$\frac{dp_i}{d\tau} = -\frac{\partial H_\omega}{\partial q_i}, \tag{S.7}$$

$$\frac{dq_i}{d\tau} = \frac{\partial H_\omega}{\partial p_i}, \tag{S.8}$$

and it is given by:

$$\mathscr{A}(p,\tau) = -H_\omega \tau - \int^p \sum_i q_i(p) dp_i + \mathscr{A}_0(p) = -H_\omega \tau - \int^\tau \left(\sum_{i=1}^{N_G} q_i(s)\dot{p}_i(s) + \varepsilon \sum_{i,j=1}^{N_G} q_{ij}(s)\dot{p}_{ij}(s)\right) ds + \mathscr{A}_0(p) \tag{S.9}$$

where the first (second) summatory is over the set of slow (fast) variables, and $\mathscr{A}_0(p)$ is an integration constant, whose value is chosen so that $\mathscr{A}(p=1,\tau) = 0$ for all τ.

We define $A_0(\tau)$ as the value of the action functional Eq. (S.9) calculated on the path which maximises its value, i.e. the optimal path. To determine the optimal path we need to solve the variational problem $\delta\mathscr{A} = 0$. The Euler-Lagrange equations associated with this variational problem are the Hamilton equations corresponding to the Hamiltonian Eq. (S.3):

$$\frac{dp_i}{d\tau} = -\frac{\partial H_\omega}{\partial q_i}, \tag{S.10}$$

$$\frac{dq_i}{d\tau} = \frac{\partial H_\omega}{\partial p_i}, \tag{S.11}$$

for the slow variables, and

$$\varepsilon \frac{dp_{ij}}{d\tau} = -\frac{\partial H_\omega}{\partial q_{ij}}, \tag{S.12}$$

$$\varepsilon \frac{dq_{ij}}{d\tau} = \frac{\partial H_\omega}{\partial p_{ij}}, \tag{S.13}$$

for the fast variables, with $\varepsilon = E/S$. These equations are (formally) solved with boundary conditions [10]: $q_i(0) = q_{0_i}$, $q_{ij}(0) = q_{0_{ij}}$, $p_i(\tau) = p_i$, and $p_{ij}(0) = p_{0_{ij}}$.

Eqs. (S.10)-(S.13) show that, if the scaling Eq. (S.3) and $\varepsilon = E/S \ll 1$ hold, our optimal path theory exhibits separation of time scales, which can be exploited to simplify our analysis by means of a multiscale asymptotic approximation.

Multi-scale analysis: Outer regime. Multi-scale asymptotic analysis [25] is predicated upon the construction of two approximations: one which is valid in the long term, the so-called *outer solution*, and another one which approximates the behaviour of the system at shorter times, the so-called, *inner solution*. These two regimes must satisfy the appropriate matching conditions which ensure that both solutions produce a uniformly valid approximation. The outer solution is usually obtained in terms of the quasi-steady state approximation, which describes the dynamics of the system once it has settled down onto the associated invariant manifold. Eqs. (S.10)-(S.13) are the starting point for the formulation of the semi-classical quasi-steady state approximation (SCQSSA) [6, 7]. The QSS approximation consists on assuming that $\varepsilon\frac{dp_{ij}}{d\tau} \simeq 0$ and $\varepsilon\frac{dq_{ij}}{d\tau} \simeq 0$ in Eqs. (S.12)-(S.13),

$$-\frac{\partial H_\omega}{\partial q_{ij}} = 0, \tag{S.14}$$

$$\frac{\partial H_\omega}{\partial p_{ij}} = 0, \tag{S.15}$$

resulting in a differential-algebraic system of equations which provides us with the semi-classical quasi-steady state approximation (SCQSSA).

Multi-scale analysis: Inner regime. To obtain the equations of the inner approximation, one usually proceeds first to re-scale the variable τ: $T \equiv \varepsilon^{-1}\tau$. Under this re-scaling the equations for the slow variables Eqs. (S.10)-(S.11) become:

$$\frac{dp_i}{dT} = O(\varepsilon), \tag{S.16}$$

$$\frac{dq_i}{dT} = O(\varepsilon), \tag{S.17}$$

which, at the lowest order, imply that $p_i(T) \simeq p_i(T=0) \equiv p_{0_i}$ and $q_i(T) \simeq q_i(T=0) \equiv q_{0_i}$ within the inner regime. By contrast, the equations for the fast variables now read:

$$\frac{dp_{ij}}{dT} = -\left.\frac{\partial H_\omega}{\partial q_{ij}}\right|_{p_{ll}(T)=p_{0_{ll}}, q_{ll}(T)=q_{0_{ll}}}, \tag{S.18}$$

$$\frac{dq_{ij}}{dT} = \left.\frac{\partial H_\omega}{\partial p_{ij}}\right|_{p_{ll}(T)=p_{0_{ll}}, q_{ll}(T)=q_{0_{ll}}}, \tag{S.19}$$

where the index $l = 1, \ldots, N_G$.

Multiscale optimal path theory: estimation of the GRN relaxation time upon epigenetic switch

The general procedure to compute the Hamiltonian associated with the characteristic function PDE, Eq. (S.2), is described in Section *General setting: WKB and multiscale optimal path theory*. In this section we provide the details of the optimal path theory analysis of the stochastic model of the gene regulatory network with competitive inhibition, as described in Section *General description of the stochastic model of an epigenetically-regulated gene regulatory network* and in S2 Table of the main text, using the procedure outlined in Section *General setting: WKB and multiscale optimal path theory*. According to such procedure, the resulting Hamiltonian, $H_k(p,Q)$, associated with the generating function PDE can be written as:

$$\begin{aligned} H_k(p,Q) = \quad & \sum_{i=1}^{N_G}(p_i-1)\left(\hat{R}_i+k_{i1}p_{ii}Q_{ii}-k_{i2}Q_i\right)+ \\ & +\sum_{i,j=1}^{N_G}\left(p_{ij}-p_j^2\right)\left(b_{ij}\left(e_i-\sum_{k=1}^{N_G}p_{ik}Q_{ik}\right)Q_j^2-u_{ij}Q_{ij}\right) \end{aligned} \tag{S.20}$$

where the pairs (p_i,Q_i) and (p_{ij},Q_{ij}) are associated with the random variables X_i, i.e. the number of protein molecules transcribed by gene i, and X_{ij}, namely, the number of sites in the promoter of gene i bound to dimers of protein j, respectively. The remaining parameters are in S2 Table main text.

According to the multiscale optimal path theory formulation, the Hamiltonian Eq. (S.20) satisfies the following scaling relationship:

$$H_k(p,Q) = b_{11}ES^2H_\omega(p,q).$$

where the re-scaled variables and parameters are defined in Table A. The re-scaled Hamiltonian, $H_\omega(p,q)$, is given by:

$$\begin{aligned} H_\omega(p,q) = \quad & \sum_{i=1}^{N_G}(p_i-1)\left(R_i+\omega_{i1}p_{ii}q_{ii}-\omega_{i2}q_i\right)+ \\ & +\sum_{i,j=1}^{N_G}\left(p_{ij}-p_j^2\right)\left(\beta_{ij}\left(p_{\infty_i}p-\sum_{k=1}^{N_G}p_{ik}q_{ik}\right)q_j^2-\delta_{ij}q_{ij}\right) \end{aligned} \tag{S.21}$$

where $p_{\infty_i}p=\frac{e_i}{E}$. The remaining re-scaled variables and parameters are given in Table A.

Table A: Re-scaled variables and dimensionless parameters for the GRN model (see S2 Table, main text, for further details).

Rescaled variables	Dimensionless parameters
$\tau = b_{11}ESt$	$\varepsilon = E/S$, $R = \hat{R}/(b_{11}ES^2)$
$q_i = Q_i/S$	$\omega_{i1} = k_{i1}/(b_{11}S^2)$, $\omega_{i2} = k_{i2}/(b_{11}ES)$,
$q_{ij} = Q_{ij}/E$	$\beta_{ij} = b_{ij}/b_{11}$, $\delta_{ij} = u_{ij}/(b_{11}S^2)$

Eq. (S.21) allows to write down the set of Hamilton equations which determine the optimal fluctuational path. These equations read:

$$\frac{dq_i}{d\tau} = R_i+\omega_{i1}p_{ii}q_{ii}-\omega_{i2}q_i-2p_j\sum_{i=1}^{N_G}\left(\beta_{ij}\left(p_{\infty_i}p-\sum_{k=1}^{N_G}p_{ik}q_{ik}\right)q_j^2-\delta_{ij}q_{ij}\right) \tag{S.22}$$

$$\varepsilon\frac{dq_{ij}}{d\tau} = (p_i-1)\omega_{i1}q_{ii}\delta_{ij}+\beta_{ij}\left(p_{\infty_i}p-\sum_{k=1}^{N_G}p_{ik}q_{ik}\right)q_j^2-\delta_{ij}q_{ij}-\left(\sum_{k=1}^{N_G}\left(p_{ik}-p_k^2\right)\beta_{ik}q_{0_k}^2\right)q_{ij} \tag{S.23}$$

$$\frac{dp_i}{d\tau} = (p_i-1)\omega_{i2}-\sum_{l=1}^{N_G}\left(p_{li}-p_i^2\right)\left(\beta_{li}\left(p_{\infty_i}p-2\sum_{k=1}^{N_G}a_{lk}p_{lk}q_{lk}\right)q_i\right) \tag{S.24}$$

$$\varepsilon\frac{dp_{ij}}{d\tau} = -(p_i-1)\omega_{i1}p_{ii}\delta_{ij}+\left(\sum_{l=1}^{N_G}\left(p_{il}-p_l^2\right)\beta_{il}\right)p_{ij}+\left(p_{ij}-p_j^2\right)\delta_{ij} \tag{S.25}$$

Eqs. (S.22)-(S.25) show that the scaling of the Hamiltonian and its associated generalised coordinates, shown in Table A, makes evident that this system exhibits multiple time scales structure where the pairs of coordinates (p_i, q_i) are slow variables which control the long-time behaviour of the system, whereas the pairs (p_{ij}, q_{ij}) are fast variables, which very quickly settle onto the invariant manifold determined by the quasi-steady approximation.

Multi-scale analysis: outer solution. The outer (long time) solution of the optimal path theory equations, Eqs. (S.10)-(S.13) is obtained by applying the QSS approximation to the fast variables, i.e. the pairs (p_{ij}, q_{ij}). It is straightforward to verify that, by taking $\varepsilon \dot{p}_{ij} \simeq 0$ and $\varepsilon \dot{q}_{ij} \simeq 0$, for all i and j, and setting $p_i(\tau) = 1$ for all i within the outer regime, and after some algebra, we obtain the associated SCQSSA equations:

$$\frac{dq_i}{d\tau} = R_i + p_{\infty_i} p \omega_{i1} \frac{\beta_{ii} q_i^2}{\delta_{ii} + \delta_{ii} \sum_k \frac{\beta_{ik}}{\delta_{ik}} q_k^2} - \omega_{i2} q_i \tag{S.26}$$

$$q_{ij}(\tau) = p_{\infty_i} p \frac{\beta_{ij} q_j^2}{\delta_{ij} + \delta_{ij} \sum_k \frac{\beta_{ik}}{\delta_{ik}} q_k^2} \tag{S.27}$$

$$p_{ij}(\tau) = 1 \tag{S.28}$$

We have shown elsewhere [1, 6, 7] that the quantities $p_{\infty_i} p$ are given by:

$$p_{\infty_i} p = \frac{e_i}{E}$$

where e_i is the number of binding sites within the promoter of gene i. The mean-field limit of Eqs. (S.26)-(S.27) is dictated by taking $p_{\infty_i} p = 1$ for all i which prescribes that the number of binding sites in all genes is exactly equal to its average over a population of cells. It is therefore clear that the SCQSSA equations with $p_{\infty_i} p \neq 1$ will, in general, behave differently than their mean-field counterparts. In particular, Eqs. (S.26)-(S.27) may allow transitions between states that are forbidden by the mean-field dynamics. This possibility will be discussed at length in the Section *Benchmark: Stochastic model of a single self-activating gene* in connection to the robustness of phenotypic states (see also de la Cruz et al. [7]).
Furthermore, it is straightforward to verify that the action functional Eq. (S.9) calculated on the outer solution, $\mathscr{A}_{QSS}$, at the lowest order vanishes: $\mathscr{A}_{QSS} = 0$. Therefore, the only positive contribution to the action must come from the inner solution.

Multi-scale analysis: inner solution and matching conditions. According to the analysis carried out in Section *Multi-scale optimal path theory: estimation of the GRN relaxation time upon epigenetic switch*, in order to fully characterise the behaviour of the stochastic competitive binding model, we must address the short time regime and formulate the appropriate matching condtions with the SCQSSA.

The matching conditions are prescribed as follows:

1. The outer solution is such that $p_i(\tau) = 1$ for all i. This implies that, within the inner regime, where $p_i(T) \simeq$cnt. $\equiv p_{0_i}$, then, $p_{0_i} = 1$ for all i.

2. The SCQSS approximation Eqs. (S.26)-(S.28) is such that, on the SCQSSA trajectory, $H_\omega = 0$. Therefore, the inner solution and the initial conditions should be such that

$$H_\omega \left(p_{0_i} = 1, p_{ij}(T), q_{0_i}, q_{ij}(T) \right) = 0.$$

3. The outer solution is such that $p_{ij}(\tau) = 1$ for all i and j. Therefore,

$$\lim_{T \to \infty} p_{ij}(T) = 1$$

Taking into account Matching Condition 1 and Eqs. (S.26)-(S.28), the *inner Hamiltonian*, $H_{\omega_{in}}(p_{ij}, q_{ij}) \equiv$
$H_\omega \left(p_{0_i} = 1, p_{ij}(T), q_{0_i}, q_{ij}(T) \right)$:

$$H_{\omega_{in}}(p_{ij}, q_{ij}) = \sum_{i,j=1}^{N_G} (p_{ij} - 1) \left(\beta_{ij} \left(p_{\infty_i} p - \sum_{k=1}^{N_G} p_{ik} q_{ik} \right) q_{0_j}^2 - \delta_{ij} q_{ij} \right) \tag{S.29}$$

Since, as per Matching Condition 2, the inner solution must satisfy $H_{\omega_{in}}(p_{ij}, q_{ij}) = 0$, this yields to two solutions: the so-called fast mode,

$$p_{ij} = 1, \tag{S.30}$$

for all i and j, which implies that the action integral vanishes on the fast mode. The so-called slow mode is given by the solution of the following system of algebraic equations:

$$\beta_{ij}\left(p_{\infty_i}p - \sum_{k=1}^{N_G} p_{ik}q_{ik}\right)q_{0_j}^2 - \delta_{ij}q_{ij} = 0 \tag{S.31}$$

for all i and j.

Eqs. (S.31) are such that:

$$q_{ik} = \frac{\beta_{ik}\delta_{ij}q_{0_k}^2}{\beta_{ij}\delta_{ik}q_{0_j}^2}q_{ij} \tag{S.32}$$

Using Eqs. (S.32), Eqs. (S.31) become:

$$\beta_{ij}q_{0_j}^2 p_{\infty_i}p - \delta_{ij}\left(\sum_{k=1}^{N_G}\frac{\beta_{ik}}{\delta_{ik}}p_{ik}q_{0_k}^2 + 1\right)q_{ij} = 0$$

which implies that:

$$q_{ij}(T) = p_{\infty_i}p\frac{\beta_{ij}q_{0_j}^2}{\delta_{ij} + \delta_{ij}\sum_k \frac{\beta_{ik}}{\delta_{ik}}q_{0_k}^2 p_{ik}(T)} \tag{S.33}$$

Note that the slow mode cannot be determined by energy conservation, $H_\omega(p,q) = 0$, alone. In order to circumvent this obstacle, we proceed as follows. Eqs. (S.33) must be supplemented with two extra equations, which are provided by the (inner) equations of motion, in particular the equations for the fast generalised coordinates $q_{ij}(T)$. Taking into account that $p_i(T) = 1$ and that the slow mode must satisfy Eqs. (S.33), the equations of motion for the fast generalised coordinates $q_{ij}(T)$, read:

$$\frac{dq_{ij}}{dT} = -\left(\sum_{l=1}^{N_G}(p_{il} - 1)\beta_{il}q_{0_l}^2\right)q_{ij}. \tag{S.34}$$

From Eqs. (S.34), it is straightforward to verify that:

$$\frac{\dot{q}_{ij}}{\dot{q}_{ik}} = \frac{dq_{ij}}{dq_{ik}} = \frac{q_{ij}}{q_{ik}}, \tag{S.35}$$

We now turn our attention to the *inner action integral*, $\mathscr{A}_{in}$, which is given by Eq. (S.9) computed on the slow mode:

$$\mathscr{A}_{in} = -\varepsilon\int_0^\infty\left(\sum_{i,j=1}^{N_G} q_{ij}(T)\frac{dp_{ij}}{dT}\right)dT, \tag{S.36}$$

Before proceeding further, we define $\mathscr{A}_{in} = \sum_{i=1}^{N_G}\mathscr{A}_{in_i}$, where:

$$\mathscr{A}_{in_i} \equiv -\varepsilon\int_0^\infty\left(\sum_{j=1}^{N_G} q_{ij}(T)\frac{dp_{ij}}{dT}\right)dT \tag{S.37}$$

Consider now Eq. (S.37). Integrating by parts we obtain:

$$\mathscr{A}_{in_i} = -\varepsilon\left(\sum_{j=1}^{N_G} p_{ij}q_{ij}\Bigg|_{T=0}^{T=\infty} - \int_0^\infty \dot{q}_{ii}\left(p_{ii} + \sum_{j\neq i} p_{ij}\frac{\dot{q}_{ij}}{\dot{q}_{ii}}\right)dT\right),$$

which, since $\frac{\dot{q}_{ij}}{\dot{q}_{ii}} = \frac{q_{ij}}{q_{ii}}$ (see Eqs. (S.35)), becomes:

$$\mathscr{A}_{in_i} = -\varepsilon \left(\sum_{j=1}^{N_G} p_{ij} q_{ij} \Bigg|_{T=0}^{T=\infty} - \int_0^\infty \frac{\dot{q}_{ii}}{q_{ii}} \left(\sum_{j=1}^{N_G} p_{ij} q_{ij} \right) dT \right),$$

Furthermore, Eq. (S.31) implies that:

$$\sum_{j=1}^{N_G} p_{ij} q_{ij} = p_{\infty_i} p - \frac{\delta_{ii}}{\beta_{ii} q_{0_i}^2} q_{ii},$$

therefore:

$$\mathscr{A}_{in_i} = \varepsilon \left(\frac{\delta_{ii}}{\beta_{ii} q_{0_i}^2} q_{ii} \Bigg|_{q_{0_{ii}}}^{q_{ii_{out}}} + \int_{q_{0_{ii}}}^{q_{ii_{out}}} \left(\frac{p_{\infty_i} p}{q_{ii}} - \frac{\delta_{ii}}{\beta_{ii} q_{0_i}^2} \right) dq_{ii}, \right) = \varepsilon p_{\infty_i} p \log \left(\frac{q_{ii_{out}}}{q_{0_{ii}}} \right), \quad \text{(S.38)}$$

where

$$q_{ii_{out}} = p_{\infty_i} p \frac{\beta_{ii} q_{0_i}^2}{\delta_{ii} + \delta_{ii} \sum_k \frac{\beta_{ik}}{\delta_{ik}} q_{0_k}^2},$$

which is dictated by the matching conditions, and $q_{0_{ii}} = q_{ii}(T=0)$. Therefore, $\mathscr{A}_{in}$ is given by

$$\mathscr{A}_{in} = \varepsilon \left(\sum_{i=1}^{N_G} p_{\infty_i} p \log \left(\frac{q_{ii_{out}}}{q_{0_{ii}}} \right) \right) \quad \text{(S.39)}$$

where, for $\mathscr{A}_{in} > 0$, it is sufficient that the initial conditions for q_{ij} satisfy that $q_{0_{ii}} < q_{ii_{out}}$. The quantities $q_{0_{ii}} = q_{ii}(T=0)$ must be such that $H_\omega(p_{0_i}, p_{0_{ij}}, q_{0_i}, q_{0_{ij}}) = 0$. Moreover, since $p_{0_i} q_{0_i} = \frac{X_i(t=0)}{S}$ and $p_{0_{ij}} q_{0_{ij}} = \frac{X_{ij}(t=0)}{E}$, besides the energy condition, $p_{0_i} q_{0_i} \geq 0$ and $0 \leq p_{0_{ij}} q_{0_{ij}} \leq \frac{e_i}{E}$ must hold for all i, j.

By insering Eq. (S.39) into the WKB *Ansatz*, we obtain that $P_+(\mathbf{B}_i|\mathbf{N})$, with $\mathbf{N} = (X_1, \ldots, X_{N_G})$, is a multinomial distribution since the correponding generating function, $G(p_1, \ldots, p_{N_G})$, is given by:

$$G(p_{i1}, \ldots, p_{iN_G}) = \left(\frac{1 + \sum_k \frac{\beta_{ik}}{\delta_{ik}} x_k^2 p_{ik}}{1 + \sum_k \frac{\beta_{ik}}{\delta_{ik}} x_k^2} \right)^{e_i} \quad \text{(S.40)}$$

where we have taken $x_k = p_{0_k} q_{0_k} = q_{0_k}$.

STOCHASTIC MODEL REDUCTION METHOD

We present a summarised version of the asymptotic model reduction. The starting point of our analysis is the so-called Poisson representation of the stochastic process, which is equivalent to the Master Equation, [26]:

$$X_i(t) = X_i(0) + \sum_{k=1}^{R_G} r_{G_{ik}} \mathscr{P} \left(\int_0^t W_{ik}(\mathbf{X}(s); \mathbf{Y}_i(s)) ds \right) \quad \text{(S.41)}$$

$$X_{ij}(t) = X_{ij}(0) + \sum_{k=1}^{R_G} r_{G_{ijk}} \mathscr{P} \left(\int_0^t W_{ik}(\mathbf{X}(s); \mathbf{Y}_i(s)) ds \right) \quad \text{(S.42)}$$

$$Y_{ij}(t) = Y_{ij}(0) + \sum_{k=1}^{R_E} r_{E_{ijk}} \mathscr{P} \left(\int_0^t V_{ik}(\mathbf{Y}_i(s)) ds \right) \quad \text{(S.43)}$$

where X_i denotes the product of gene i, X_{ij} refers to the number of dimers of type j bound to the promoter region of gene i and Y_{ij} corresponds to the number of molecular species of type j within the ER model of gene i. We also use the notation $\mathbf{X} = (X_1, \ldots, X_{N_G}, X_{11}, \ldots, X_{N_G N_G})$ and $\mathbf{Y}_i = (Y_{i1}, \ldots, Y_{i7})$. $\mathscr{P}(\lambda) \sim \text{Poisson}(\lambda)$, i.e. $\mathscr{P}(\lambda)$ is a random number sampled from a Poisson distribution with parameter λ [26], R_G and R_E denote the total number of reactions in the GRN model (see S2 Table, main text) and in the ER model (see S3 Table, main text), respectively, with W_{ik} and V_{ik} denoting the transition rates corresponding to the GRN model and the ER model (see S2 Table and S3 Table, main text, respectively). The stoichiometries

$r_{G_{ik}}$, $r_{G_{ijk}}$ and $r_{E_{ijk}}$ denote the change in number of molecules that reaction k has on X_i, X_{ij} and Y_{ij}, respectively. Eqs. (S.41) and (S.42) are associated with the the stochastic dynamics of the GRN (see S2 Table, main text), which are regulated by the ER part of the model. Eq. (S.43) describes the dynamics of the ER system, which drives the dynamics of the GRN (see S2 Table and S3 table, main text).

Under the appropriate conditions, separation of time scales can be made explicit by re-scaling the random variables and the transition rates. Based on our previous work [1, 6, 7], we propose the following rescaling:

$$\begin{aligned}
&X_i = Sx_i,\, X_{ij} = Ex_{ij},\\
&Y_{ij} = Yy_{ij},\ \text{for } j = 1,2,3,\, Y_{ij} = Zy_{ij},\ \text{for } j = 4,5,6,7\\
&W_{ik}(\mathbf{X};\mathbf{Y}_i) = b_{11}ES^2 w_k(\mathbf{x};\mathbf{y}_i),\, V_{ik}(\mathbf{Y}_i) = c_{14}ZY^2 v_{ik}(\mathbf{y}_i).
\end{aligned} \tag{S.44}$$

In Eq. (S.44), the scale factors S, E, Y, and Z are the characteristic number of protein transcripts, promoter region binding sites, histone modification sites, and epigenetic enzymes (HDMs and HDACs), respectively. For simplicity, we assume that these scales are the same for all the genes involved in the GRN. We assume that $S \gg E \simeq Y \gg Z$. We further define a re-scaled (dimensionless) time: $\tau = b_{11}ESt$. The parameters b_{11}, c_{14} and the transition rates W_{ik} and V_{ik} are as described in S2 and S3 Table, main text.

By using Eq. (S.44) in Eqs. (S.41)-(S.43), we obtain:

$$x_i(\tau) = x_i(0) + \sum_{k=1}^{R_G} r_{G_{ik}} \frac{1}{S} \mathscr{P}\left(S \int_0^\tau w_k(\mathbf{x}(\sigma);\mathbf{y}_i(\sigma))d\sigma\right)\ \text{(slow)} \tag{S.45}$$

$$x_{ij}(\tau) = x_{ij}(0) + \sum_{k=1}^{R_G} r_{G_{ijk}} \frac{1}{E} \mathscr{P}\left(E \frac{1}{\varepsilon_1} \int_0^\tau w_k(\mathbf{x}(\sigma);\mathbf{y}_i(\sigma))d\sigma\right)\ \text{(fast)} \tag{S.46}$$

$$y_{ij}(\tau) = y_{ij}(0) + \sum_{k=1}^{R_E} r_{E_{ijk}} \frac{1}{Y} \mathscr{P}\left(Y \frac{1}{\varepsilon_2} \int_0^\tau v_{ik}(\mathbf{y}_i(\sigma))d\sigma\right),\ j = 1,2,3\ \text{(slow)} \tag{S.47}$$

$$y_{il}(\tau) = y_{il}(0) + \sum_{k=1}^{R_E} r_{E_{ilk}} \frac{1}{Z} \mathscr{P}\left(Z \frac{1}{\varepsilon_2} \frac{1}{\varepsilon_3} \int_0^\tau v_{ik}(\mathbf{y}_i(\sigma))d\sigma\right),\ l = 4,5,6,7\ \text{(fast)} \tag{S.48}$$

where $\varepsilon_1 = \frac{E}{S} \ll 1$, $\varepsilon_2 = \frac{b_{11}S}{c_{14}Z}$, and $\varepsilon_3 = \frac{Z}{Y} \ll 1$, with $\varepsilon_1 < \varepsilon_3$. We have no direct information to estimate the order of magnitude of ε_2. Thus, without loss of generality we will assume that $\varepsilon_2 = \mathscr{O}(1)$.

The scaling hypothesis $S \gg E \simeq Y \gg Z$ allows for a series of successive approximations which enables us to reduce the model Eqs. (S.45)-(S.48) into a much less computationally demanding system. First, provided that both $\varepsilon_1 \ll 1$ and $\varepsilon_3 \ll 1$, we can assume that the (rescaled) rates associated with the fast variables GRN-ER dynamics (Eqs. (S.46) and (S.48)) are much larger than those corresponding to their slow counterparts (Eqs. (S.45) and (S.47)). Under these conditions, the stochastic dynamics of the fast variables reaches their (quasi-)steady states while the slow variables are effectively frozen [27–30].

We proceed with the asymptotic model reduction by first addressing the quasi-steady state approximation (QSSA) probability density functions (PDFs) of the fast variables (see *Inner solution* below). We then move on to study the QSS approximation of the slow variables, in particular, the large-S asymptotics of the protein concentration dynamics (see *Outer solution* below).

Inner solution The inner solution corresponds to the relaxation dynamics of the fast variables onto their quasi-equilibrium state, while the slow variables remain unchanged. The solution of the inner dynamics allows us to determine the QSSA PDFs of the fast variables conditioned to fixed values of the slow variables.

We proceed by considering the following rescaling of the time variable $T = \varepsilon_1^{-1}\tau$. Upon such rescaling, it is straightforward that all the rates of the reactions affecting the slow variables (Eqs. (S.45) and (S.47)) are now $\mathscr{O}(\varepsilon_1)$, which implies that the slow variables, x_i and y_{ij} (for $j = 1,2,3$), can be considered to remain frozen whilst the fast variables reach their quasi-equilibrium distribution according to the dynamics:

$$x_{ij}(T) = x_{ij}(0) + \sum_{k=1}^{R_G} r_{G_{ijk}} \frac{1}{E} \mathscr{P}\left(E \int_0^T w_k(\mathbf{x}(\sigma); \mathbf{y}_i(\sigma)) d\sigma \right) \tag{S.49}$$

$$y_{il}(T) = y_{il}(0) + \sum_{k=1}^{R_E} r_{E_{ilk}} \frac{1}{Z} \mathscr{P}\left(Z \frac{c_{14}\varepsilon_1}{b_{11}\varepsilon_3} \int_0^T v_{ik}(\mathbf{y}_i(\sigma)) d\sigma \right) \tag{S.50}$$

where $l = 4,5,6,7$, $\varepsilon_2 = \mathscr{O}(1)$ and the slow variables, x_i and y_{ij}, $j = 1,2,3$, are considered to stay constant.

Consider the (inner) dynamics of the (rescaled) number of bound sites within the promoter regions, $x_{ij}(T)$, Eq. (S.49). Provided that the ER of gene i remains in the open state, i.e. $y_{i2} \ll 1$ and $y_{i3} \sim \mathscr{O}(1)$, the resulting stochastic dynamics describes how the binding sites switch between bound-to-TF dimer to unbound-to-TF dimer at constant rates (since the number of the different TF molecules does not change at this time scale). Since the number of binding sites is a constant, the (quasi-)steady state distribution of bound TFs to each promoter is a multinomial (see Section *Multi-scale optimal path theory: estimation of the GRN relaxation time upon epigenetic switch* for a detailed derivation of this result). Otherwise, if gene i is epigenetically closed, then $X_{ij}(T) = 0$ for all j with probability one. Therefore, the random vector describing the number of TFs bound at the promoter region of gene i, $\mathbf{B}_i$, whose components are $\mathbf{B}_i = (X_{i1}, \ldots, X_{iN_G})$ is sampled from:

$$P(\mathbf{B}_i|\mathbf{N}) = \eta_i P_+(\mathbf{B}_i|\mathbf{N}) + (1-\eta_i) P_-(\mathbf{B}_i|\mathbf{N}), \tag{S.51}$$

where $\mathbf{N} = (X_1, \ldots, X_{N_G})$ is a vector containing the monomer gene product of all genes, $P_-(\mathbf{B}_i|\mathbf{N}) = \prod_{j\in\langle i\rangle} \delta_{X_{ij},0}$, with $\delta_{X_{ij},0} = 1$ when $X_{ij} = 0$, and $P_+(\mathbf{B}_i|\mathbf{N})$ is a multinomial PDF, whose generating function is given by:

$$G(p_{i1}, \ldots, p_{iN_G}) = \left(\frac{1 + \sum_k \frac{\beta_{ik}}{\delta_{ik}} x_k^2 p_{ik}}{1 + \sum_k \frac{\beta_{ik}}{\delta_{ik}} x_k^2} \right)^{e_i} \tag{S.52}$$

where e_i denotes the number of binding sites at the promoter region of gene i, and other parameters are defined in S2 Table, main text. The quantity η_i is defined as: $\eta_i = H(Y_{i3} - Y_0)$ (i.e. gene i is epigenetically open if the corresponding level of acetylation, Y_{i3}, exceeds the threshold Y_0).

Eq. (S.50) describes the inner dynamics of the fast components (enzymes and enzyme-substrate complexes) of the ER system for each gene i. The resulting stochastic dynamics describes how the enzymes switch between their free state and their complex state at constant rates (as the number of the different substrates is constant under the hypothesis of time scale separation). Since the number of enzymes is conserved, the (quasi-)steady distribution of the number of enzymes of each type in complex form is a binomial (see Section *Quasi-steady state distribution of the enzyme/complex sub-system (ER)* for a detailed derivation of this result). The corresponding generating functions are given by:

$$G_{HDM}(p_i) = \left(\frac{\kappa_{i2} + \kappa_{i3} + (\kappa_{i5} + \kappa_{i6}) y_{i3} + (\kappa_{i1} + y_{i3}) y_{i2} p_i}{(\kappa_{i2} + \kappa_{i3}) + (\kappa_{i1} + y_{i3}) y_{i2} + (\kappa_{i5} + \kappa_{i6}) y_{i3}} \right)^{e_{HDM}} \tag{S.53}$$

$$G_{HDAC}(p_i) = \left(\frac{\kappa_{i10} + \kappa_{i11} + (\kappa_{i13} + \kappa_{i14}) y_{i2} + (\kappa_{i9} + \kappa_{i12} y_{i2}) y_{i3} p_i}{(\kappa_{i10} + \kappa_{i11}) + (\kappa_{i9} + \kappa_{i12} y_{i2}) y_{i3} + (\kappa_{i13} + \kappa_{i14}) y_{i2}} \right)^{e_{HDAC}} \tag{S.54}$$

where $i = 1, \ldots, N_G$ and κ_{ij} are defined in S3 Table, main text. The number of free HDM and HDAC molecules is then obtained from the conservation equations $Y_{i4} = e_{HDM} - Y_{i5}$ and $Y_{i6} = e_{HDAC} - Y_{i7}$.

Outer solution The outer solution, corresponding to the dynamical evolution of the slow variables, is obtained by sampling the fast variables, whose values are needed to compute the reaction rates for the slow variables, from their QSSA PDFs (see Eqs. (S.52)-(S.54)):

$$x_i(\tau) = x_i(0) + \sum_{k=1}^{R_G} r_{G_{ik}} \frac{1}{S} \mathscr{P}\left(S \int_0^\tau w_k(\mathbf{x}(\sigma); \mathbf{y}_i(\sigma)) d\sigma \right) \tag{S.55}$$

$$y_{ij}(\tau) = y_{ij}(0) + \sum_{k=1}^{R_E} r_{E_{ijk}} \frac{1}{E} \mathscr{P}\left(\frac{E}{\varepsilon_2} \int_0^\tau v_{ik}(\mathbf{y}_i(\sigma)) d\sigma \right), \quad j = 1,2,3 \tag{S.56}$$

The QSSA PDFs of the fast variables are conditioned by the current value of the slow variables. We complete our asymptotic analysis by looking at the large S behaviour of the slow GRN variables (see Eq. (S.45)). We resort to a law of large numbers enunciated and proved by Kurtz which states that $S^{-1}\mathscr{P}(Su) \to u$ when $S \gg 1$ [19, 26, 29, 31]. We can apply this result straightforwardly to Eq. (S.55), which eventually leads to the asymptotic reduction of the full ER-GRN system:

$$\frac{\mathrm{d}x_i}{\mathrm{d}\tau} = R_i + \omega_{i1}x_{ii} - \omega_{i2}x_i - 2\sum_{j=1}^{N_G}\left(\beta_{ij}\eta_i\left(\frac{e_j}{E} - \sum_{k=1}^{N_G} x_{jk}\right)x_i^2 - \delta_{ij}x_{ij}\right), i = 1,\dots,N_G \tag{S.57}$$

$$y_{ij} = y_{ij}(0) + \sum_{k=1}^{R_E} r_{E_{ijk}}\frac{1}{E}\mathscr{P}\left(E\frac{1}{\varepsilon_2}\int_0^\tau v_{ik}(y_i(\sigma))d\sigma\right), j = 1,2,3, \tag{S.58}$$

The resulting dynamics consists on a hybrid system where the dynamics of the TF monomers, $x_i(\tau)$, Eq. (S.57), is described in terms of a piece-wise deterministic Markov process [32, 33], i.e. by a system of ODEs perturbed at discrete times by two random processes, one corresponding to stochastic ER (Eq. (S.58)) and the other to TF dimers binding to the promoter regions. The latter are sampled from their QSSA PDFs, Eq. (S.51). The stochastic dynamics of the slow ER variables, Eq. (S.58), is in turn coupled to the random variation of the associated fast variables (ER enzymes, HDM and HDAC, and complexes). The number of complexes, Y_{i5} and Y_{i7}, are sampled from their QSSA PDFs, Eqs. (S.53) and (S.54). The corresponding numerical method used to simulate such system is described in detail in S1 Appendix.

CONSISTENCY WITH THE STOCHASTIC MODEL REDUCTION METHOD

GRN model

In this section, we check the consistency between the multiscale WKB method (Section *Multiscale analysis of the GRN system: WKB approximation and multiscale optimal path theory*) and the model reduction methodology (Section *Stochastic model reduction method*). For concreteness, we focus the discussion in the derivation of the quasi-steady state distribution of the binding/unbinding sub-system (GRN). As discussed previously, the fast variables of the GRN sub-model, i.e. the TF-promoter binding site dimers, are in quasi-equilibrium with the slow variables, i.e. the protein products [27–30]. We therefore consider an (*inner*) approximation where the slow variables are frozen during the (fast) evolution of the binding/unbinding subsystem towards equilibrium. We start by writing a Master equation for a reduced set of reactions given by:

$$\text{Free binding sites} + 2X_j \underset{K_{ij_2}}{\overset{K_{ij_1}}{\rightleftarrows}} X_{ij} \text{ for all } i,$$

where $K_{ij_1} = k_{ij_1}(e_i - \sum_l X_{il})$, where $k_{ij_1} = b_{ij}H(Y_{i_3} - Y_0)X_j(X_j - 1)$ and X_j is taken to be fixed, and $K_{ij_2} = u_{ij}$. Recall that X_j, the number of protein transcript of gene j is a slow variable for all j, and, therefore, within the inner approximation, they are considered to be constant. The associated reduced Master Equation (RME) is given by:

$$\begin{aligned}\frac{\partial P(\mathscr{X}_i,t)}{\partial t} = & \sum_j k_{ij_1}\left(\left(e_i - \sum_{l\neq j} X_{il} - (X_{ij}-1)\right)P(\mathscr{X}_i - r_j,t) - \left(e_i - \sum_l X_{il}\right)P(\mathscr{X}_i,t)\right) \\ & + \sum_j u_{ij}\left((X_{ij}+1)P(\mathscr{X}_i + r_j,t) - X_{ij}P(\mathscr{X}_i,t)\right)\end{aligned} \tag{S.59}$$

where $\mathscr{X}_i = (X_{i1},\dots,X_{iN_G})$. Before proceeding forward, we made an additional assumption regarding time scale separation, namely, for all i, the RME Eq. (S.59) reaches its (quasi-)equilibrium state in a much shorter time than the average switching times of the ER sub-system. In other words, we can consider separately the steady state solution for open and silenced genes and that, upon ER switch, the equilibrium distribution of the binding/unbinding system "instantaneously" switches to the associated equilibrium distribution. The feasability of this assumption is illustrated below, where we analyse the case of one single self-activating gene, which can be fully solved analytically.

We thus consider two steady-state solutions of Eq. (S.59): $P_\pm(\mathscr{X}_i)$ associated with an open gene (i.e. $H(Y_{i_3} - Y_0) = 1$) and a silenced gene (i.e. $H(Y_{i_3} - Y_0) = 0$), respectively. $P_-(\mathscr{X}_i)$ is trivial: since in a silenced gene TF dimers can only detach from

the promoter, which implies that the corresponding steady state solution is:

$$P_-(\mathscr{X}_i) = \prod_{j \in \langle i \rangle} \delta_{X_{ij},0} \tag{S.60}$$

An analytical expression for $P_+(\mathscr{X}_i)$ can be found using WKB asymptotics. By re-scaling variables in RME, we derive the following partial differential equation for the associated generating function (see Section *General setting: WKB and multiscale optimal path theory*):

$$\frac{\partial G(p_{i1}, \dots, p_{iN_G}, \tau)}{\partial \tau} = \frac{1}{\varepsilon} H_{\omega_{in}}(p_{i1}, \dots, p_{iN_G}, \partial_{p_{i1}}, \dots, \partial_{p_{iN_G}}) G(p_{i1}, \dots, p_{iN_G}, \tau) \tag{S.61}$$

where $H_{\omega_{in}}(p_i, q_i)$ is derived in detail in Section *Multi-scale optimal path theory: estimation of the GRN relaxation time upon epigenetic switch* (see under heading *Multi-scale analysis: inner solution and matching conditions*), with $p_i = (p_{i1}, \dots, p_{iN_G})$ and $q_i = (q_{i1}, \dots, q_{iN_G})$, and is given by Eq. (S.29):

$$H_{\omega_{in}}(p_i, q_i) = \sum_{i,j=1}^{N_G} (p_{ij} - 1) \left(\beta_{ij} \left(\frac{e_i}{E} - \sum_{k=1}^{N_G} p_{ik} q_{ik} \right) q_{0_j}^2 - \delta_{ij} q_{ij} \right)$$

Note that since $H_{\omega_{in}}(p_i, q_i)$ is identical to the inner Hamiltonian associated with the multiscale WKB method, the corresponding WKB solution of Eq. (S.61), derived from the stochastic model reduction, is identical to the one provided by the multiscale WKB method.

Quasi-steady state distribution of the enzyme/complex sub-system (ER)

Similarly to the inner approximation obtained for the fast GRN variables, we can find a QSSA for the fast variables corresponding to the ER component of the system, namely, the enzymes and complexes associated with the ER enzymatic reactions. We will focus on the number of complexes formed, since the free enzyme molecules can be obtained using the associated conservation law [1]. We start by writing the Master Equation corresponding to a reduced set of reactions given by:

$$\text{Substrate} + Y_{ij-1} \underset{K_{ij_2}}{\overset{K_{ij_1}}{\rightleftarrows}} Y_{ij} \text{ for all } i \text{ and } j = 5, 7$$

which affect only the fast ER variables (see S3 Table in the main text). The number of substrate molecules is a slow variable for all i and j, and, therefore, within the inner approximation is considered constant. According to S3 Table of the main text, and denoting by $\varepsilon_1 = \frac{E}{S}$ and $\varepsilon_3 = \frac{Z}{Y}$, the corresponding rates are $K_{i5_1} = \frac{\varepsilon_1}{\varepsilon_3}(\kappa_{i1} + \kappa_{i4} Y_{i3})(e_{HDM} - Y_{i5}) Y_{i2}$, $K_{i5_2} = \frac{\varepsilon_1}{\varepsilon_3}(\kappa_{i2} + \kappa_{i5} Y_{i3}) Y_{i5}$, $K_{i7_1} = \frac{\varepsilon_1}{\varepsilon_3}(\kappa_{i9} + \kappa_{i12} Y_{i2})(e_{HDAC} - Y_{i7}) Y_{i3}$, and $K_{i7_2} = \frac{\varepsilon_1}{\varepsilon_3}(\kappa_{i10} + \kappa_{i13} Y_{i2}) Y_{i7}$, where Y_{i2} and Y_{i3} are taken to be fixed. The corresponding Master Equation can be solved using the same method used for the fast GRN variables. The resulting QSSA PDF generating functions are:

$$G_\infty(p_{i5}) = \left(\frac{\kappa_{i2} + \kappa_{i3} + (\kappa_{i5} + \kappa_{i6}) y_{i3} + (\kappa_{i1} + y_{i3}) y_{i2} p_{i5}}{(\kappa_{i2} + \kappa_{i3}) + (\kappa_{i1} + y_{i3}) y_{i2} + (\kappa_{i5} + \kappa_{i6}) y_{i3}} \right)^{e_{HDM}} \tag{S.62}$$

$$G_\infty(p_{i7}) = \left(\frac{\kappa_{i10} + \kappa_{i11} + (\kappa_{i13} + \kappa_{i14}) y_{i2} + (\kappa_{i9} + \kappa_{i12} y_{i2}) y_{i3} p_{i7}}{(\kappa_{i10} + \kappa_{i11}) + (\kappa_{i9} + \kappa_{i12} y_{i2}) y_{i3} + (\kappa_{i13} + \kappa_{i14}) y_{i2}} \right)^{e_{HDAC}} \tag{S.63}$$

where $y_{i2} = Y_{i2}/Z$, $y_{i3} = Y_{i3}/Z$, and the parameters κ_{ij} are defined in S3 Table of the main text.

The number of complexes Y_{i5} and Y_{i7} are sampled from the binomial distributions related to the generating functions Eqs. (S.62) and (S.63). The corresponding number of free enzymes is then obtained using the conservation laws: $Y_{i4} = e_{HDM} - Y_{i5}$ and $Y_{i6} = e_{HDAC} - Y_{i7}$, with e_{HDM} and e_{HDAC} denoting the total number of HDM and HDAC enzymes, respectively.

BENCHMARK: STOCHASTIC MODEL OF A SINGLE SELF-ACTIVATING GENE

We use the simplest case of a single self-activating gene as benchmark to check the accuracy of the multiscale analysis introduced in Section *Multiscale analysis of the GRN system: WKB approximation and multiscale optimal path theory*. This example can be analysed in detail and we use it as an illustrative example which can help to shed some light onto more complex situations. The dynamics of this system is described by the Chemical Master Equation (CME) with transition rates W_j given in

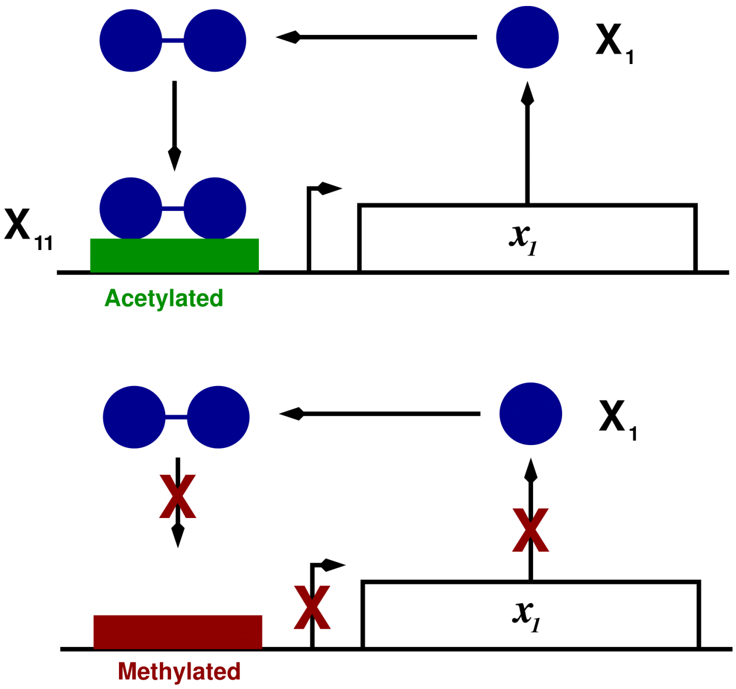

Figure A: Schematic representation of the model of self-activation gene regulatory circuit with epigenetic regulation. The gene product X_1 is its own transcription factor which, upon dimerisation, binds the promoter region of the gene thus triggering gene transcription. The transition rates corresponding to this gene regulatory circuit are given in Table C. For simplicity, we use an effective model in which the formation of the dimer and binding to the promoter region is taken into account in a single reaction, and the resulting number of promoter sites bound by two transcription factors is denoted by X_{11} (see Table B). Furthermore, depending on whether the epigenetic state is predominantly acetylated or methylated, the promoter region of the gene is accessible or inaccessible to the transcription factor, respectively.

Table B: Random variables associated with the stochastic dynamics of an auto-activation gene regulatory circuit [34, 35]. See Fig. A for an schematic representation.

Variable	Description
X_1	Number of transcription factor molecules
X_{11}	Number of bound promoter sites in the gene promoter region

Table C: Transition rates associated with the stochastic dynamics of an auto-activation gene regulatory circuit [34, 35]. e_1 corresponds to the number of binding sites within the gene's promoter region. See Fig. A for an schematic representation.

Transition rate	r	Event
$W_1(x) = \hat{R} + k_1 X_{11}$	$r_1 = (1,0)$	Synthesis of the transcription factor
$W_2(x) = k_2 X_1$	$r_2 = (-1,0)$	Degradation of the transcription factor
$W_3(x) = b_{11} X_1 (X_1 - 1)(e_1 - X_{11})$	$r_3 = (-2,+1)$	Dimer binding to the gene promoter region
$W_4(x) = u_{11} X_{11}$	$r_4 = (+2,-1)$	Unbinding from the gene promoter region

Table C. A slightly modified version of this system has been analysed in de la Cruz et al. [7] using the methods proposed by Alarcón [6]. This methodology is based on optimal path theory (OPT) [11] with separation of time scales. The latter is used to formulate a quasi-steady state approximation (QSSA) of the Euler-Lagrange (Hamilton) equations of the optimal path.

Robustness of the active state: assessing the accuracy of the multiscale OPT predictions. Using the methodology outlined in Section *Multi-scale optimal path theory: estimation of the GRN relaxation time upon epigenetic switch*, de la Cruz et al. [7] have derived the SCQSSA equations for the stochastic model of a self-activating gene:

$$\frac{dq_1}{d\tau} = R + \omega_1 p_{\infty_1} p \frac{q_1^2}{\delta_{11} + q_1^2} - \omega_2 q_1, \tag{S.64}$$

$$q_{11} = p_{\infty_1} p \frac{q_1^2}{\delta_{11} + q_1^2}. \tag{S.65}$$

where p and p_{∞_1} are parameters such that $p_{\infty_1} p = \frac{e_1}{E}$ [1, 6, 7]. The rescaled variables and parameters are defined in Table D. Fig. B shows how the steady state behaviour of the Eqs. (S.64)-(S.65) changes as $p_{\infty_1} p$ varies. We set our system up so that its mean-field limit, corresponding to $p_{\infty_1} p = 1$ [10], exhibits bistability. We observe that, as $p_{\infty_1} p$ decreases (i.e. as e_1 decreases), the system goes through a saddle-node bifurcation whereby the active state (i.e. the steady state where $q_1 = O(1)$) loses stability. Therefore, our stochastic SCQSSA predicts a noise-induced transition from the active state into the inactive state (i.e. the steady state such that $q_1 \ll O(1)$)), provided that $\frac{e_1}{E}$ is smaller than the critical value of $p_{\infty_1} p$, which we refer to as $(p_{\infty_1} p)_{cr}$. This scenario has been fully explored and verified by means of direct stochastic simulations by de la Cruz et al. [7]. Therefore, the robustness of the active state is quantified by the action integral computed on such trajectory [10, 11].

It is straightforward to verify the action integral Eq. (S.9) computed on the outer solution, A_{QSS}, satisfies that $A_{QSS} = 0$. This is because $H_\omega(p,q) = 0$, $p_1(\tau) = 1$, and $\varepsilon \dot{p}_{11} \simeq 0$. Therefore, in order to quantify the robustness of the system, we must resort to the analysis of the inner regime. The result of this analysis, which is carried out in detail in the Section *Multi-scale optimal path theory: estimation of the GRN relaxation time upon epigenetic switch*, see Eq. (S.39), is that there exists a solution of the inner dynamics with a positive contribution to the action, $\mathscr{A}_{in}$:

$$\mathscr{A}_{in} = \frac{e_1}{S} \log \frac{q_{11_{out}}}{q_{0_{11}}} \tag{S.66}$$

where

$$q_{11_{out}} = p_{\infty_1} p \frac{q_{0_1}^2}{\delta_{11} + q_{0_1}^2}.$$

We have further used that $\varepsilon = E/S$, $p_{\infty_1} p = e_1/E$. This quantity and $q_{0_{11}} = q_{11}(T=0)$ must be such that $H_\omega(p_{0_1}, p_{0_{11}}, q_{0_1}, q_{0_{11}}) = 0$. Moreover, since $p_{0_1} q_{0_1} = \frac{X_1(t=0)}{S}$ and $p_{0_{11}} q_{0_{11}} = \frac{X_{11}(t=0)}{E}$, besides the energy condition, $p_{0_1} q_{0_1} \geq 0$ and $0 \leq p_{0_{11}} q_{0_{11}} \leq \frac{e_1}{E}$ must hold. Furthermore, q_{0_1} must belong to the basin of attraction of the active steady state of Eqs. (S.64)-(S.65).

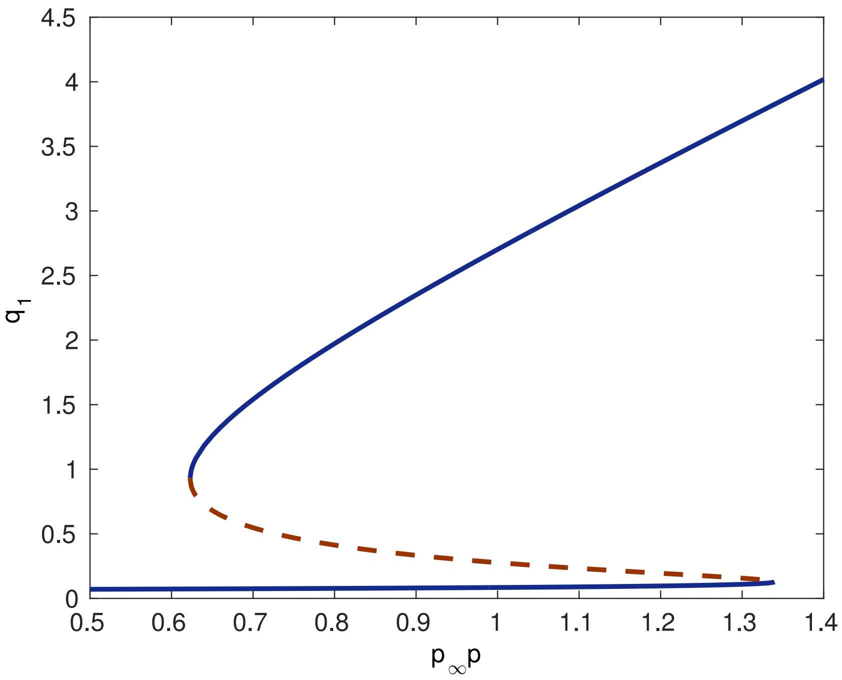

Figure B: Bifurcation analysis for the SCQSS approximation of the stochastic auto-activation gene regulatory circuit Eq. (S.64), with mean-field transcription rate $\omega_1 = 3.0$. Parameter values as given in Table D. See de la Cruz et al. [7] for details.

The estimate for the robustness of the active state in this simple gene regulatory network within our multiscale optimal path theory is therefore given by [13]:

$$A_0 = \begin{cases} \frac{e_1}{S}\log\left(\frac{q_{11_{out}}}{q_{0_{11}}}\right) & \text{if } \frac{e_1}{E} < (p_{\infty_1}p)_{cr} \\ \mathscr{O}(1) & \text{if } \frac{e_1}{E} \geq (p_{\infty_1}p)_{cr} \end{cases} \tag{S.67}$$

Eqs. (S.64) and (S.67) allow for several numerically testable predictions. Below the critical value, i.e. for $\frac{e_1}{E} < (p_{\infty_1}p)_{cr}$, we have that:

$$\tau_E \sim \exp\left(e_1 \log\left(\frac{q_{11_{out}}}{q_{0_{11}}}\right)\right),$$

whereas, if $\frac{e_1}{E} \geq (p_{\infty_1}p)_{cr}$

$$\tau_E \sim \mathscr{O}\left(e^S\right),$$

which means that the below-critical escape time from the active state grows exponentially with the number of binding sites in the promoter of the gene. This prediction of our multiscale optimal path theory analysis has been validated by direct stochastic simulations of the stochastic model of the self-activating gene regulatory circuit, Table C [5, 36], which are shown in Fig. C.

Model reduction: assessing the accuracy of the asymptotic analysis and the numerical method. Using the single self-activating gene regulatory network, we want to assess the accuracy of our numerical hybrid method (see S1 Appendix, main text, for details). Therefore, Eq. (S.57) reads in this case as:

$$\frac{dx_1}{d\tau} = R_1 + \omega_1 x_{11} - \omega_2 x_1 - 2\left(\beta_{11}\eta_i\left(\frac{e_1}{E} - x_{11}\right)x_1^2 - \delta_{11}x_{11}\right), \tag{S.68}$$

where $x_{11} = \frac{X_{11}}{E}$ and X_{11} is sampled from (see Eq. (S.51)):

$$P_1(X_{11}|x_1) = H(Y_{i3} - Y_0)P_{1_+}(X_{11}|x_1) + H(Y_0 - Y_{i3})P_{1_-}(X_{11}|x_1), \tag{S.69}$$

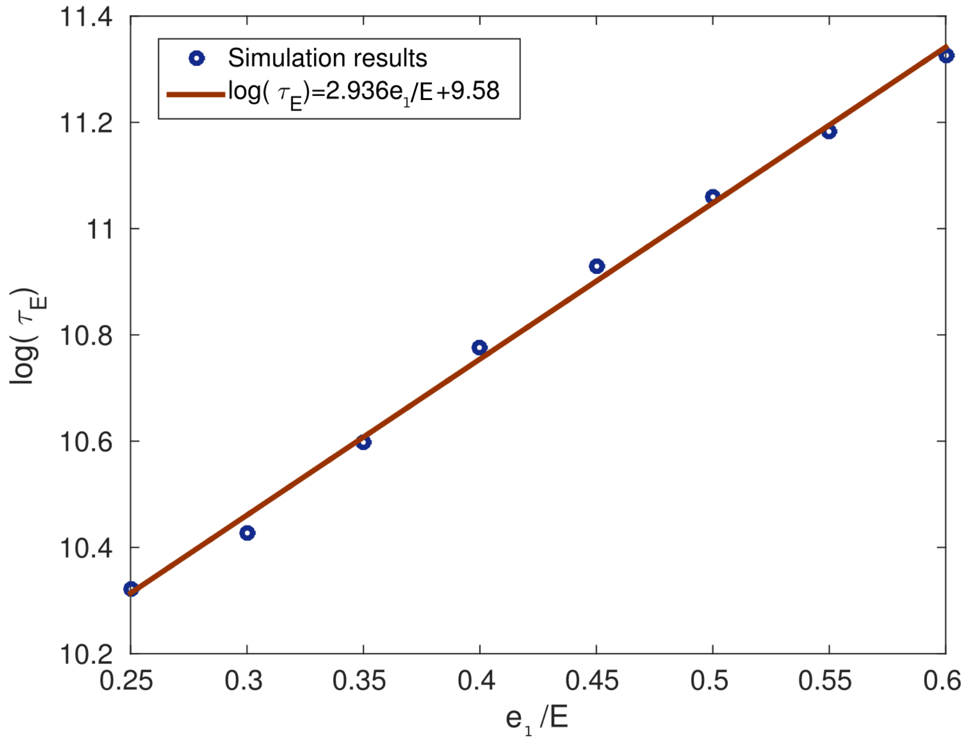

Figure C: This plot shows simulation results regarding the average exit time from the active steady state. We have plotted how the average exit time, τ_E, changes as e_1, i.e. the total number of binding sites in the promoter region of the self-activating gene, is let to vary. We show our results as a log-linear plot. This figure validates our result that the below critical exit time varies exponentially with e_1. Blue circles represent simulation results. The red line represents a least-squares fitting of the simulation data. The coefficients of this fitting are within the 95% confidence interval. Parameter values for the stochastic simulations have been taken from Tables D and A. Furthermore $\omega_1 = 3.0$. Averages are performed over 1000 realisations for each data point.

Table D: Parameter values used in simulations of the stochastic self-activating single gene regulatory circuit. See de la Cruz et al. [7] for details.

Rescaled parameter	Parameter	Units	Reference
$\omega_1 = \frac{a}{k_{deg}\sqrt{K_d}}$	$K_d = 10$	nM	[34]
$\omega_2 = 1$	$k_{deg} = 2$	min^{-1}	[34]
$\delta_{11} = 1$	$r = 0.4$	$\text{nM} \cdot \text{min}^{-1}$	[34]
$R = \frac{r}{k_{deg}\sqrt{K_d}}$	$S = 1000$		–
$b_{11}ES = k_{deg}$	$E = 20$		–

where $P_{1_+}(X_{11}|x_1)$ is a binomial distribution whose generating function is $G_\infty(p_{11}|x_1) = (1+\pi_{11}(x_1)(p_{11}-1))^{e_1}$ where

$$\pi_{11}(x_1) = \frac{x_1^2}{\delta_{11}+x_1^2},$$

and $P_{1_-}(X_{11}|x_1) = \delta_{X_{11},0}$. We have used this simple model to test the accuracy of the asymptotic model reduction and the associated numerical method. The results are shown in Fig. D where we compare the performance of our approximation against stochastic simulation algorithm results. SSA is feasible for this simple model which allows a systematic comparison with our approximation. For more complex systems, SSA is computationally too costly, and an alternative is necessary, which is the motivation behind our asymptotic analysis.

To carry out the comparison between the asymptotic reduction and the SSA, we have simulated a (very long) realisation where we let the system evolve towards the open gene steady-state (Figs. D(a), (e), and (i)). Over this long realisation, we have collected statistics regarding three quantities: x_1, x_{11}, and π_{11}. The asymptotic reduction fares rather well against the SSA when we take $\Delta\tau$ to be up to an order of magnitude larger than the average time step in the SSA simulation. This is a rather substantial improvement in computational performance. Taking larger values of $\Delta\tau$, in our case $\Delta\tau > 0.5\varepsilon$, with $\varepsilon = 10^{-2}$, distorts the statistics and the results of the asymptotic reduction are no longer accurate.

We have tried the $\tau-$leap version of the algorithm (results not shown) but with no improvement in performance relative to the fixed time step method. The $\Delta\tau$ selection method used in our simulations is the one proposed in [37]. The issue with these simulations is that occasionally the $\Delta\tau$ selection procedure produces a value of the time step that is exceedingly large. This compromises the stability of the method and its accuracy. Further work, beyond the scope of the present work, needs to be done regarding adaptive time step versions of the asymptotic reduction method.

SUMMARY OF THE MINIMUM ACTION PATH THEORY AND NUMERICAL METHOD

The Freidlin-Wentzell (FW) theory of large deviations provides a framework to understand the effects of small random perturbations in dynamical systems. According to large deviation principles, statistics of rare events, when they occur, will follow the least unlikely path with high probability. In our model, the switch between the open and closed states (or vice versa) of the ER model is the rare event that we want to study (see Section *Transitions between ER states:minimum action path approach*, main text, for further details).The key element of the FW theory is an action functional whose minimiser gives the stochastic trajectory of maximum likelihood by which this event happens [38]. Therefore, our problem of finding a stochastic trajectory is translated into finding the trajectory that minimizes a deterministic functional. Let us denote by φ a given trajectory, and by φ_* the trajectory that minimises the functional. An explicit form of the functional, denoted by $\mathscr{A}_{FW}(\varphi(\tau))$, can be given if the dynamics is given by the corresponding chemical Langevin equation [37],

$$\mathrm{d}y_{ij}(\tau) = f_j(\mathbf{y}_i)\mathrm{d}t + g_{ij}(\mathbf{y}_i)\mathrm{d}B_{j_t}, \tag{S.70}$$

where B_{j_t} denotes a Wiener process, and the mean-field drift, $f_j(\mathbf{y}_i)$, is $f_j(\mathbf{y}_i) = \sum_{k=1}^{R_E} r_{E_{ijk}} v_{ik}(\mathbf{y}_i)$, and the noise matrix, $g_{ij}(\mathbf{y}_i)$, $g_{jj} = \sqrt{\sum_{k=1}^{R_E} r_{E_{ijk}}^2 v_{ik}(\mathbf{y}_i)}$, $g_{ij} = 0$ if $i \neq j$, with R_E denoting the total number of reactions in the ER model and $r_{E_{ijk}}$ denoting the change in number of molecules that reaction k has on y_{ij} (see S3 Table, main text). The rescaled variables y_{ij}, rescaled vector $\mathbf{y}_i = (y_{ij} = \frac{Y_{ij}}{Y})$, $j = 1,\ldots,7$, and the rescaled rates $v_{ik}(\mathbf{y}_i)$ are defined in S3 Table, main text. In this case, the action functional $\mathscr{A}_{FW}(\varphi(\tau))$ is the Freidlin-Wentzel (FW) functional:

$$\mathscr{A}_{FW}(\varphi) = \int_0^\tau ||\dot{\varphi}(t) - f(\varphi(t))||^2_{g(\varphi(t))} dt, \tag{S.71}$$

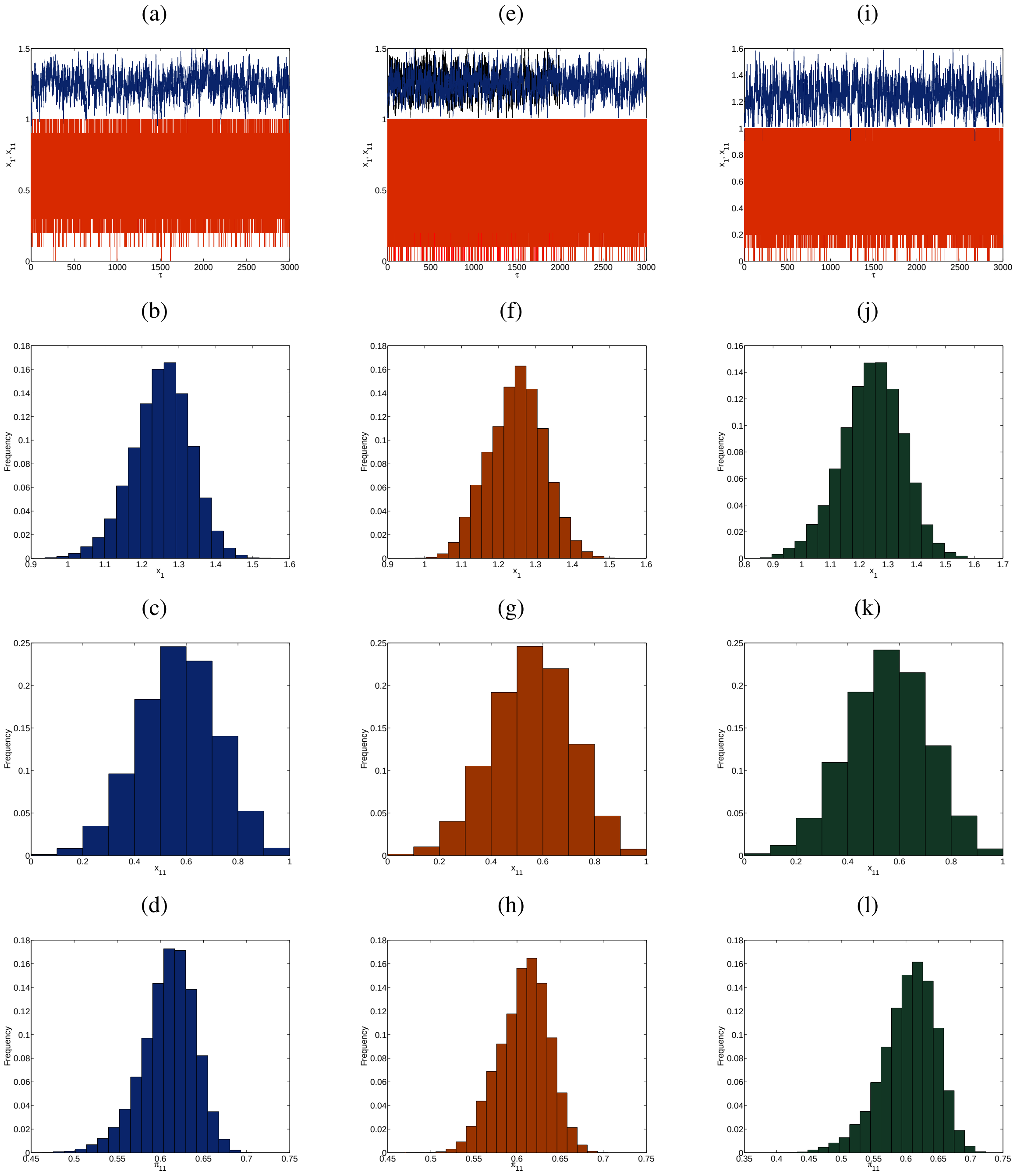

Figure D: Results for simulations of the aysmptotic model reduction for the stochastic model of the self-activating gene with fixed $\Delta\tau$. Series of plots comparing a number of statistics regarding the stochastic simulation of the self-activating GRN using Gillespie's stochastic algorithm (plots (a), (b), (c), and (d)) and the aysmptotic model reduction (plots (e), (f), (g), (h) with fixed time step $\Delta\tau = 0.25\varepsilon = 2.5 \cdot 10^{-3}$, and plots (i), (j), (k), (l) with fixed time step $\Delta\tau = 0.5\varepsilon = 5 \cdot 10^{-3}$). The average time step in the Gillespie simulation is $\Delta\tau = 2.6946 \cdot 10^{-4}$. The mean and standard deviation of x_1 in the Gillespie simulation are $\langle x_1 \rangle = 1.2532$ and $\sigma_{x_1} = 0.0794$, respectively. According to simulation of the asymptotic model reduction these quantities are $\langle x_1 \rangle = 1.2488$ and $\sigma_{x_1} = 0.0752$, $\langle x_1 \rangle = 1.2431$ and $\sigma_{x_1} = 0.1072$ for $\Delta\tau = 0.25\varepsilon$ and $\Delta\tau = 0.5\varepsilon$, respectively. Parameter values given in Table D and $a = 1.95$.

The norm $||\cdot||^2_{g(\varphi(t))} = \left\langle \cdot, D(\varphi(t))^{-1} \cdot \right\rangle$, where $D(\varphi(t)) = g(\varphi(t))\, g(\varphi(t))^T$ is the diffusion tensor. Using Eq. (S.71), the optimal value of the action can be found by numerical minimisation. In our case, the minimization is done by a custom made genetic algorithm that explores different paths in the $n+1$ dimensional space, where one dimension corresponds to the parameter which parameterizes the path and the remaining n dimensions correspond to the number of chemical species involved in the model.

The action minimisation was performed by finding the path $\varphi(s) = (y_1(s), \ldots, y_7(s)) : s = [0,1] \to \mathbb{R}^7$, that minimises the time independent gMAM functional [38] given an initial and final point, $\varphi(0)$ and $\varphi(1)$, respectively:

$$\mathscr{S}(\varphi(s)) = \int_0^1 |f(s)|_a (1 - \cos \eta_a(s)) |\hat{n}(s)|_a^2 \mathrm{d}s \tag{S.72}$$

where f is the deterministic component of the Langevin equation and $\hat{n}$ is a unit vector tangent to φ at a path position s (see Eq. (S.70)). The position dependent diffusion matrix $a(s) = g^{\intercal}(\varphi(s))\, g(\varphi(s))$ is used to compute the inner product that defines the norm $|\cdot|_a = \sqrt{\langle \cdot, a^{-1} \cdot \rangle}$ (see Eq. (S.70)). The angle $\eta_a(s)$ is the angle between f and φ at a path position s, that is defined using the same inner product $\cos \eta_a = \frac{\langle f, a^{-1} \hat{n} \rangle}{|f|_a |\hat{n}|_a}$.

In order to find the minimum action path, the candidate paths where parametrized by $n = 11$ points along the path $\tilde{\varphi}(s) = (y_1^i(s^i), \ldots, y_7^i(s^i))$, evaluated along a set of ordered values $\{s^i \in [0,1]\}$ with $i = 1, ..., n$; $s^1 = 0$ and $s^n = 1$. Thus, each path is determined by a $8n$-dimensional vector. To evaluate the gMAM functional (S.72), each path is interpolated using Akima splines along $N = 200$ points. The minimisation was performed using a custom made evolutionary algorithm on a population of $d_{pop} = 10000$ candidate curves. Each generation of the algorithm keeps the top $d_{fit} = 2000$ curves that score the lowest action, while the rest of the curves are replaced by offspring curves resulting from random pairings from the fittest individuals. For each random parent pair from the d_{fit} best candidates, namely, $(\tilde{\varphi}', \tilde{\varphi}'')$, its offspring path, $\tilde{\varphi}'''$, is calculated by taking uniform random intermediate values between each of the $n-2$ intermediate points of its parents pair and introducing mutations by randomly perturbing the resulting curve (the first and last point, y_j^1 and y_j^n, are not modified since they correspond with the stable stationary points (see Eq. (S.70)). Namely each coordinate $y_j^{'''i}$ of an offspring is calculated as

$$y''^i_j = ((1-\rho_1) y''^i_j + y'''^i_j \rho_1) \mathrm{e}^{m\rho_2}, \tag{S.73}$$

where ρ_1 is a uniform number $U(0,1)$, ρ_2 is a Gaussian random number $N(0,1)$, and $m = 0.01$ is the mutation intensity. The parametrisation vector $\{s^{'''i}\}$ is calculated similarly while imposing that it is monotonically increasing. In order to initialise the population, the first d_{pop} trajectories are generated by sampling random points in the latin hypercube defined by $\varphi(0)$ and $\varphi(1)$ and sampling ordered uniform vectors $\{s^i\}$. In order to keep track of the convergence of the algorithm, a Limited-memory BFGS quasi-Newton method is used to find a refined local minimum on the best candidate path every $g_{grad} = 20$ generations. The simulations were accelerated by introducing resulting optimal trajectories from the L-BFGS as a new offspring curve for the next generation. The algorithm is stopped when the curves approach the same local minimum over $g_{max} = 400$ trajectories or when the slope of the loglog curve of the average action of the d_{fit} best paths, as a function of the number of generations, is small enough ($\alpha_p = 0.01$).

In order to validate the results, the algorithm was run several times taking as final result the best curve candidate. Additionally, for some candidates, different values of the parameters of the algorithm ($d_{pop}, d_{fit}, n, g_{grad}, g_{max} m, N,$ and α_p) were explored to ensure that the algorithm is not stuck in local minima.

As a summary, the minimum action path (MAP) method follows these steps:

1. **Compute stationary states of the system**: Compute the stationary states of the system for the given parameters (saddle point, y_s, and the initial and final stable states, y_{ini} and y_f).

2. **Generate initial population of paths.** Generate d_{pop} random paths that start at y_{ini} and end at y_f.

3. **Start optimization loop** Set counter $i = 1$ (number of generations of the genetic algorithm).

   Compute the fitness Compute the value of the action functional S along each path φ (individual) from the population. In order to do so, the vector force f (mean-field drift vector), the gradient of the force, and the inverse of the diffusion tensor a (a^{-1}), with $a = gg^t$ and g the noise matrix, need to be evaluated at each point of the path.

   Rank the paths according to their fitness Rank them from the lowest to the highest value of the action functional S.

   Keep the best paths This subgroup of d_{fit} paths will be carried to the next generation.

Find optimal gradient path If $i \equiv 0 \pmod{g_{grad}}$, compute a L-BFGS minimisation taking the minimum action path of the population as the initial condition. If the resulting path improves, add it to the d_{fit} subpopulation.

Check convergence If the converging conditions are met, exit the loop and take the best path as the optimum result.

Generate new paths Generate new paths by mixing and mutating the best d_{fit} paths from the generation. This is done in order to get a new population with the same number of individuals as the original one (d_{pop} paths).

Update generation counter i = i+1

ANALYSIS OF ENSEMBLE HETEROGENEITY: SIGNIFICANT DIFFERENCES

In this section, we provide the p-values of those differences found to be significant in the Kolmogorov-Smirnov (K-S) tests carried out in Section *Analysis of ensemble heterogeneity* of the main text (see details there).

Significant differences within the ensemble of DERSs

Red cluster versus blue cluster. Our analysis shows that the KS test detects significant differences for c_{1_1} (p-value 0.0349, unrecruited demethylation), $c_{1_{11}}$ (p-value 0.0062, unrecruited deacetylation), $c_{1_{15}}$ (p-value 0.0010, unrecruited acetylation), and $c_{1_{16}}$ (p-value $3.9433 \cdot 10^{-4}$, recruited acetylation).

Red cluster versus green cluster. In this case, our analysis shows that significative differences are found only for two parameter values: c_{1_3} (p-value $2.5697 \cdot 10^{-4}$, unrecruited demethylation) and $c_{1_{16}}$ (p-value 0.0307, recruited acetylation).

Blue cluster versus green cluster. Significative differences are found for the empirical distributions of c_{1_3} (p-value $2.1411 \cdot 10^{-4}$, unrecruited demethylation), c_{1_8} (p-value 0.0038, recruited methylation), $c_{1_{11}}$ (p-value 0.0156, unrecruited deacetylation), and $c_{1_{15}}$ (p-value 0.0023, unrecruited acetylation).

Significant differences between differentiation-primed and pluripotency-locked ER landscapes

We first proceed to compare within the whole population (without discriminating between clusters) those DERSs such that $\mathscr{Q} \geq \mathscr{T}$ (differentiation-primed ER landscapes) against those with $\mathscr{Q} < \mathscr{T}$ (pluripotency-locked ER landscapes). We take $\mathscr{T} = 0.7$. The parameter values for which the KS test yields significant differences when comparing the differentiation-primed ER system to the pluripotency-locked ER are c_{1_1} (p-value 0.0125, unrecruited demethylation), $c_{1_{14}}$ (p-value 0.0435, recruited deacetylation), $c_{1_{15}}$ (p-value 0.0191, unrecruited acetylation), and $c_{1_{16}}$ (p-value 0.0057, recruited acetylation).

If we now restrict our analysis to those DERSs within the blue cluster, we observe that the parameters whose CDFs differ significantly when splitted into differentiation-primed and pluripotency-locked are c_{1_1} (p-value 0.0135, unrecruited demethylation) and $c_{1_{14}}$ (p-value 0.0095, recruited deacetylation).

Regarding the PERSs, the results are shown in Fig. S.8 of the main text. Our analysis shows that significative differences can be found between the empirical distributions of three parameter values: c_{1_3} (p-value $5.9983 \cdot 10^{-6}$, unrecruited demethylation), c_{1_8} (p-value $7.4181 \cdot 10^{-4}$, recruited methylation), and $c_{1_{15}}$ (p-value 0.0047, unrecruited acetylation).

REFERENCES

1. Folguera-Blasco, N., E. Cuyàs, J. A. Menéndez, and T. Alarcón, 2018. Epigenetic regulation of cell fate reprogramming in aging and disease: A predictive computational model. *PLoS Comp. Biol.* 14:e1006052.

2. Dodd, I. B., M. A. Micheelsen, K. Sneppen, and G. Thon, 2007. Theoretical analysis of epigenetic cell memory by nucleosome modification. *Cell* 129:813–822.

3. Menéndez, J. A., B. Corominas-Faja, E. Cuyàs, M. G. García, S. Fernández-Arroyo, A. F. Fernández, J. Joven, M. F. Fraga, and T. Alarcón, 2016. Oncometabolic nuclear reprogramming of cancer stemness. *Stem Cell Reports* 6:273–283.

4. Lu, C., and C. B. Thomson, 2012. Metabolic regulation of epigenetics. *Cell Metabolism* 16:9–17.

5. Gillespie, D. T., 1976. A general method for numerically simulating the stochastic time evolution of coupled chemical reactions. *J. Comp. Phys.* 22:403–434.

6. Alarcón, T., 2014. Stochastic quasi-steady state approximations for asymptotic solutions of the Chemical Master Equation. *J. Chem. Phys.* 140:184109.

7. de la Cruz, R., P. Guerrero, F. Spill, and T. Alarcón, 2015. The effects of intrinsic noise on the behaviour of bistable systems in quasi-steady state conditions. *J. Chem. Phys.* 143:074105.

8. Dykman, M. I., T. Horita, and J. Ross, 1995. Statistical distribution and stochastic resonance in a periodically driven chemical system. *J. Chem. Phys.* 103:966–972.

9. Maier, R. S., and D. L. Stein, 1996. A scaling theory of bifurcations in the symmetric weak-noise escape problem. *J. Stat. Phys.* 83:291–357.

10. Elgart, V., and A. Kamenev, 2004. Rare events in reaction-diffusion systems. *Phys. Rev. E* 70:041106.

11. Bressloff, P. C., and J. M. Newby, 2014. Path integrals and large deviations in stochastic hybrid systems. *Phys. Rev. E* 89:042701.

12. Zhang, B., and P. G. Wolynes, 2014. Stem cell differentiation as a many-body problem. *Proc. Natl. Acad. Sci.* 111:10185–10190.

13. Wells, D. K., W. L. Kath, and A. E. Motter, 2015. Control of stochastic and induced switching in biophysical networks. *Phys. Rev. X* 5:031036.

14. Santos, F. A. N., H. Gadelha, and E. A. Gaffney, 2015. Fock space, symbolic algebra, and analytical solutions for small stochastic systems. *Phys. Rev. E* 92:062714.

15. Doi, M., 1976. Stochastic theory of diffusion-controled reaction. *J. Phys. A:Math. Gen.* 9:1479.

16. Peliti, L., 1985. Path integral approach to birth-death processes on a lattice. *J. Phys. France* 46:1469–1483.

17. Assaf, M., and B. Meerson, 2006. Spectral formulation and WKB approximation for rare-event statistics in reaction systems. *Phys. Rev. E* 74:041115.

18. Assaf, M., B. Meerson, and P. V. Sasorov, 2010. Large fluctuations in stochastic population dynamics: Momentum space calculations. *J. Stat. Mech.* P07018.

19. Kang, H.-W., and T. G. Kurtz, 2013. Separation of time-scales and model reduction for stochastic reaction networks. *The Annals of Applied Probability* 23:529–583.

20. Briggs, G. E., and J. B. S. Haldane, 1925. A note on the kinetics of enzyme action. *Biochem. J.* 19:338–39.

21. Keener, J., and J. Sneyd, 1998. Mathematical physiology. Springer-Verlag, New York, NY, USA.

22. Kubo, R., K. Matsuo, and K. Kitahara, 1973. Fuctuation and relaxation of macrovariables. *J. Stat. Phys.* 9:51–96.

23. Alarcón, T., and K. M. Page, 2007. Mathematical models of the VEGF receptor and its role in cancer therapy. *J. R. Soc. Interface* 4:283–304.

24. Gonze, D., J. Halloy, and P. Gaspard, 2002. Biochemical clocks and molecular noise: Theoretical study of robustness factors. *J. Chem. Phys.* 116:10997–11010.

25. Holmes, M. H., 1995. Introduction to perturbation methods. Springer-Verlag, New York, NY, USA.

26. Anderson, D. F., and T. G. Kurtz, 2010. Stochastic analysis of biochemical systems. Springer, New York, NY, USA.

27. Cao, Y., D. T. Gillespie, and L. R. Petzold, 2005. Multiscale stochastic simulation algorithm with stochastic partial equilibrium assumption for chemically reacting systems. *J. Comp. Phys.* 206:395–411.

28. Cao, Y., D. T. Gillespie, and L. R. Petzold, 2005. The slow-scale stochastic simulation algorithm. *J. Chem. Phys.* 122:014116.

29. Ball, K., T. G. Kurtz, L. Popovic, and G. Rempala, 2006. Asymptotic analysis of multi-scale approximations to reaction networks. *Ann. App. Prob.* 16:1925–1961.

30. Smith, S., C. Cianci, and R. Grima, 2015. Model reduction for stochastic chemical systems with abundant species. *J. Chem. Phys.* 143:214105.

31. Kurtz, T. G., 1978. Strong approximation theorems for density dependent Markov chains. *Stochastic Processes and their Applications* 6:223 – 240.

32. Davis, M. H. A., 1984. Piecewise-Deterministic Markov Processes: A General Class of Non-Diffusion Stochastic Models. *Journal of the Royal Statistical Society. Series B (Methodological)* 46:353–388.

33. Bressloff, P. C., and S. D. Lawley, 2017. Mean first passage times for piecewise deterministic Markov processes and the effects of critical points. *Journal of Statistical Mechanics: Theory and Experiment* 2017:063202.

34. Frigola, D., L. Casanellas, J. M. Sancho, and M. Ibañes, 2012. Asymmetric stochastic switching driven by intrinsic molecular noise. *PLoS One* 7:e31407.

35. Weber, M., and J. Buceta, 2013. Stochastic stablisation of phenotypic states: the genetrric bistable switch as a case study. *PLoS One* 8:e73487.

36. Gillespie, D. T., 1977. Exact stochastic simulation of coupled chemical reactions. *The journal of physical chemistry* 81:2340–2361.

37. Gillespie, D. T., 2001. Approximate accelerated stochastic simulation of chemically reacting systems. *J. Chem. Phys.* 155:1716–1733.

38. Heymann, M., and E. Vanden-Eijnden, 2007. The geometric minimum action method: A least action principle on the space of curves. *Communications on Pure and Applied Mathematics* 61:1052–1117.
