# Supplementary material for: A multiscale model of epigenetic heterogeneity-driven cell fate decision-making
Source: PLoS Comput Biol. 2019 Apr 30;15(4):e1006592. doi: 10.1371/journal.pcbi.1006592 (PMC6510448; doi:10.1371/journal.pcbi.1006592)
Supplement: S1 Appendix — (PDF) [file pcbi.1006592.s002.pdf]

In Section *Coupling ER-GRN models: Multiscale analysis and model reduction*, we have exploited separation of time scales to formulate a QSSA whereby both the number of bound sites within the promoter of the genes and the number of enzymes and complexes molecules associated with the ER enzyme kinetics are sampled from their QSSA PDFs. Furthermore, since $S \gg 1$, we have taken a large-S limit which allows us to write the dynamics of X_i, i.e. the number of protein transcripts of gene i, in terms of an ODE perturbed by two random forcings: one associated with the random (fast) binding/unbinding dynamics and another one produced by the random ER dynamics. However, as long as we assume that $\epsilon_2 = \frac{b_{11}S}{c_{14}Z} \sim \mathcal{O}(1)$ and $Y = E \ll S$, further simplification is not possible and the evolution of the slow ER variables (i.e. number of positive and negative marks, and unmarked sites) needs to be solved by numerical simulation of their stochastic dynamics. In spite of this, the reduced version of the original stochastic model (Eqs. (**??**)-(**??**)) allows for a far more efficient numerical implementation of a complex ER-GRN stochastic system.

The asymptotic reduction of the full stochastic model provides the basis for a hybrid numerical method with enhanced performance with respect to the stochastic simulation algorithm (as illustrated in Figure D in the **??**). The current hybrid method is based on that formulated in [1]. The numerical method proceeds through iteration of a basic algorithm composed of the following steps:

1. Set initial conditions for the slow variables of the GRN and ER components of the system.

2. Sample the fast variables from their QSSA PDFs conditioned to the current value of the corresponding slow variables. Their sampled values are fed in the evolution equations of the latter.

3. Consider the stochastic dynamics of the slow ER variables, Eq. (**??**). These stochastic equations must be solved by numerical simulation using Gillespie's SSA. We first set the corresponding time step, $\Delta\tau$, using the SSA.

4. Solve the ODEs for the slow variables of the GRN dynamics, Eq. (**??**), in the time interval $[\tau, \tau + \Delta\tau)$.

5. Complete the Gillespie step for the slow ER variables by choosing which elementary reaction alters the ER regulatory state and update the slow ER variables accordingly.

6. Repeat Steps 2 through to 5 until some stopping condition is satisfied.

If we are running the fixed time step version of the algorithm, Step 1 needs to be done only once during the initialisation of the algorithm.

References

1. Guerrero P, Byrne HM, Maini PK, Alarcón T. From invasion to latency: Intracellular noise and cell motility as key controls of the competition between resource-limited cellular populations. J Math Biol. 2015;72:123–156.
