# Supplementary figures and images for: A multiscale model of epigenetic heterogeneity-driven cell fate decision-making
Source: PLoS Comput Biol. 2019 Apr 30;15(4):e1006592. doi: 10.1371/journal.pcbi.1006592 (PMC6510448; doi:10.1371/journal.pcbi.1006592)

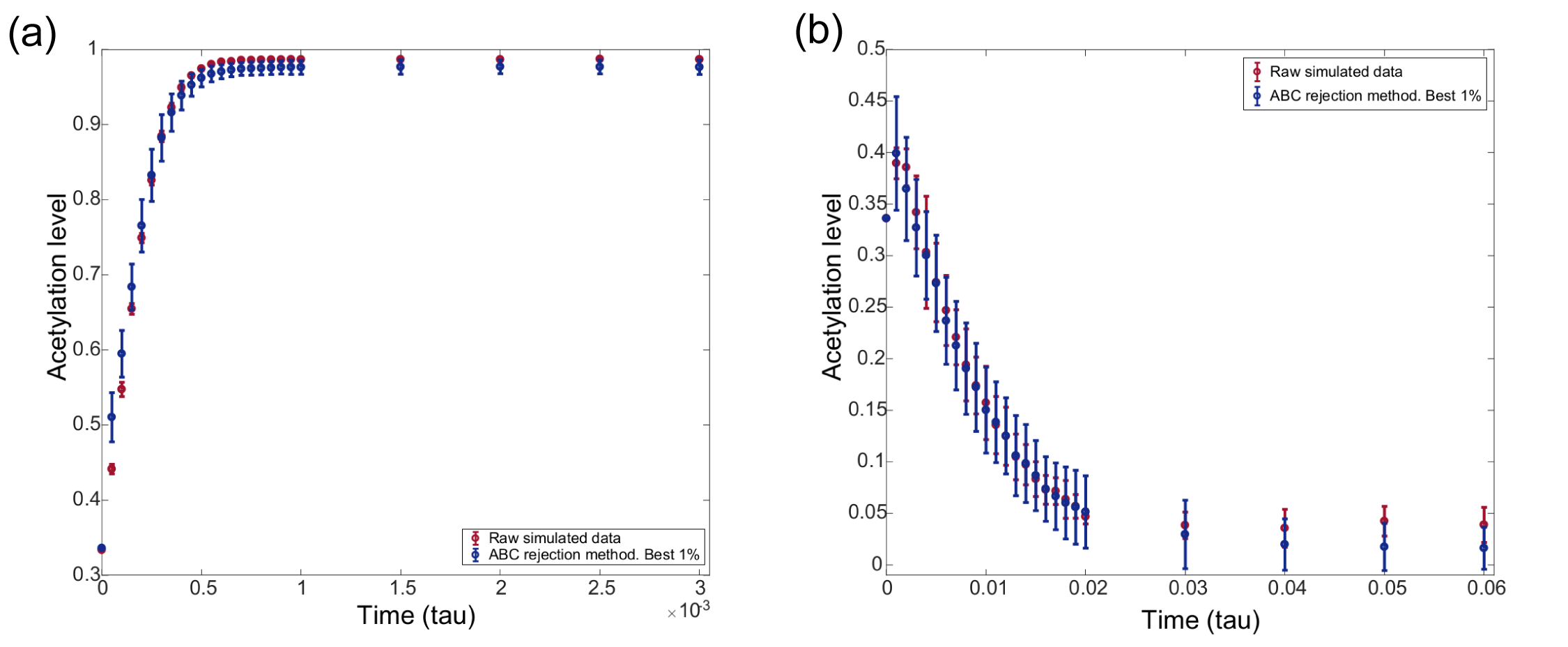

Supplement: S1 Fig — (TIF) [file pcbi.1006592.s003.tif]

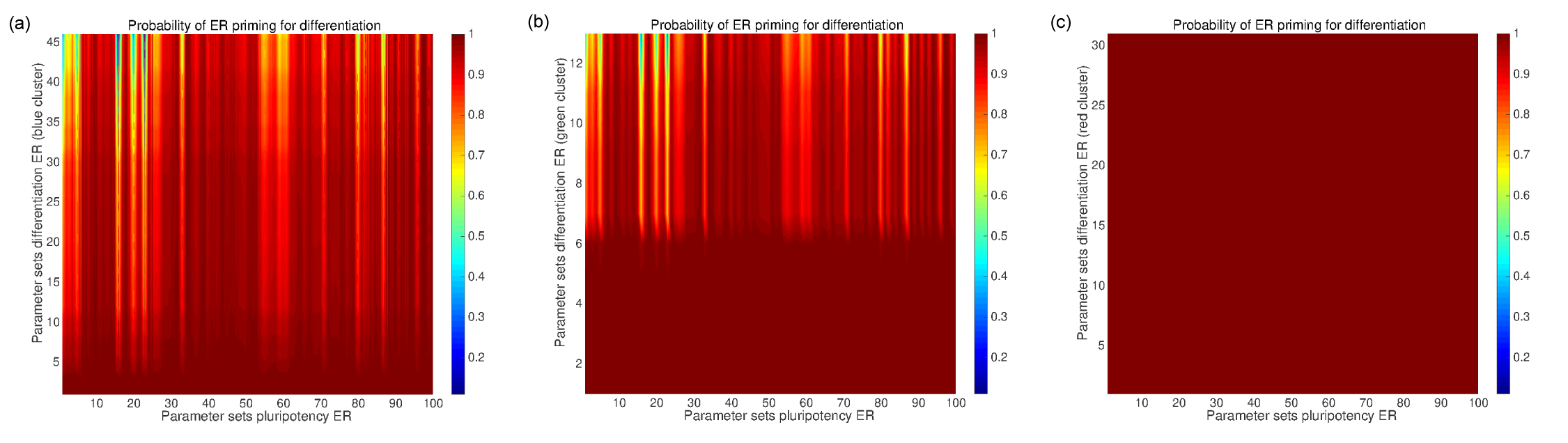

Supplement: S2 Fig — (TIF) [file pcbi.1006592.s004.tif]

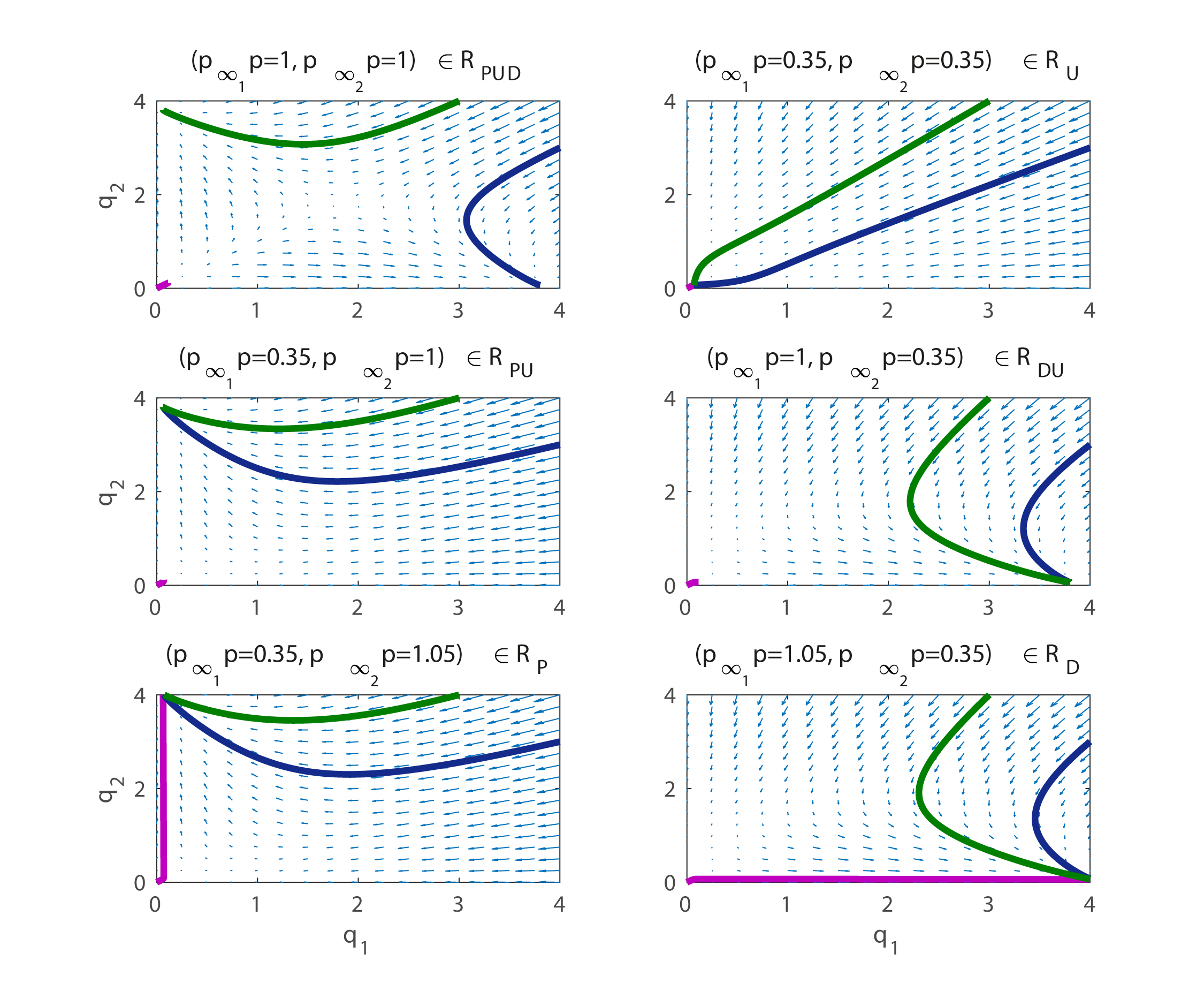

Supplement: S3 Fig — (TIF) [file pcbi.1006592.s005.tif]

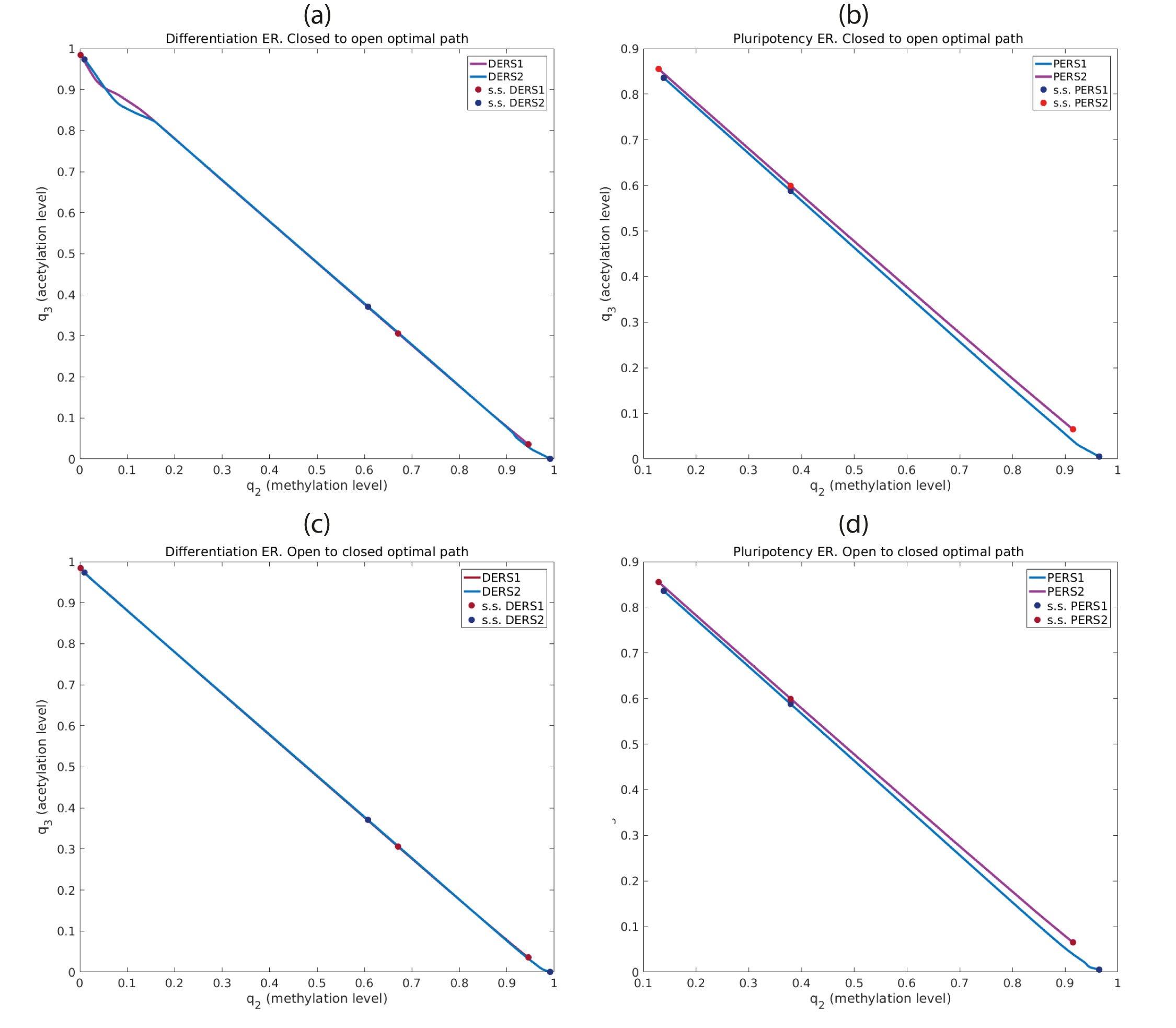

Supplement: S4 Fig — (TIF) [file pcbi.1006592.s006.tif]

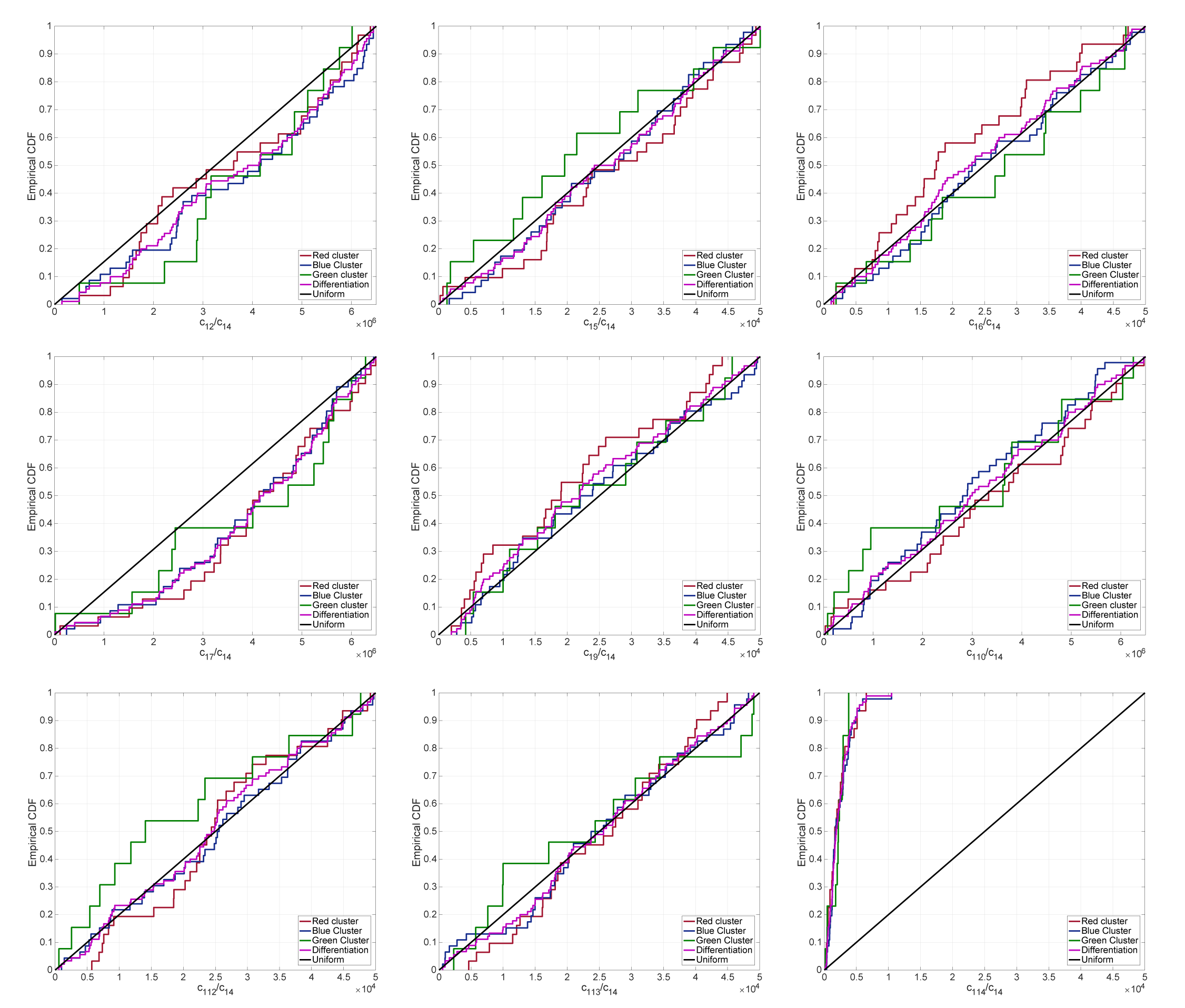

Supplement: S5 Fig — (TIF) [file pcbi.1006592.s007.tif]

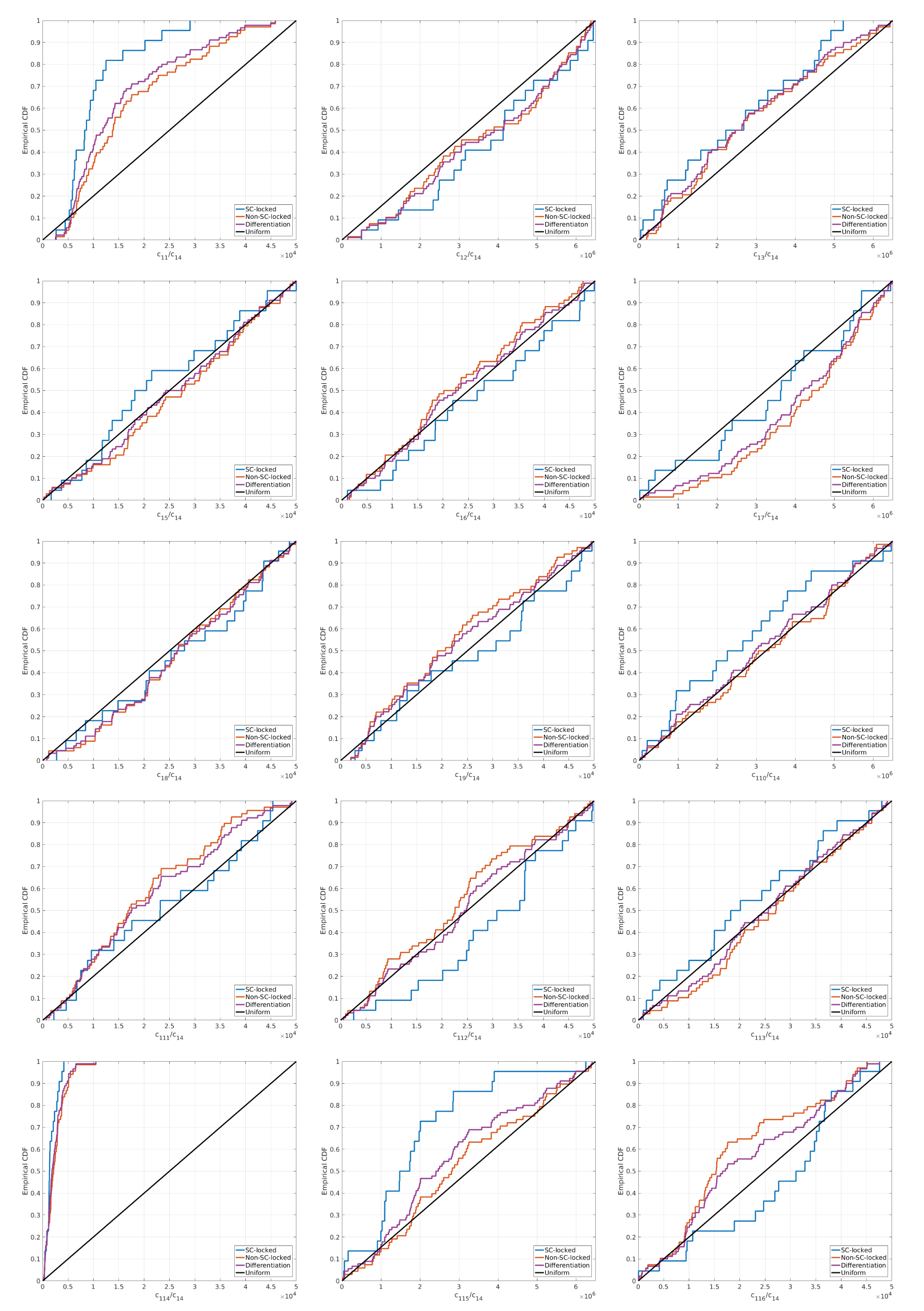

Supplement: S6 Fig — (TIF) [file pcbi.1006592.s008.tif]

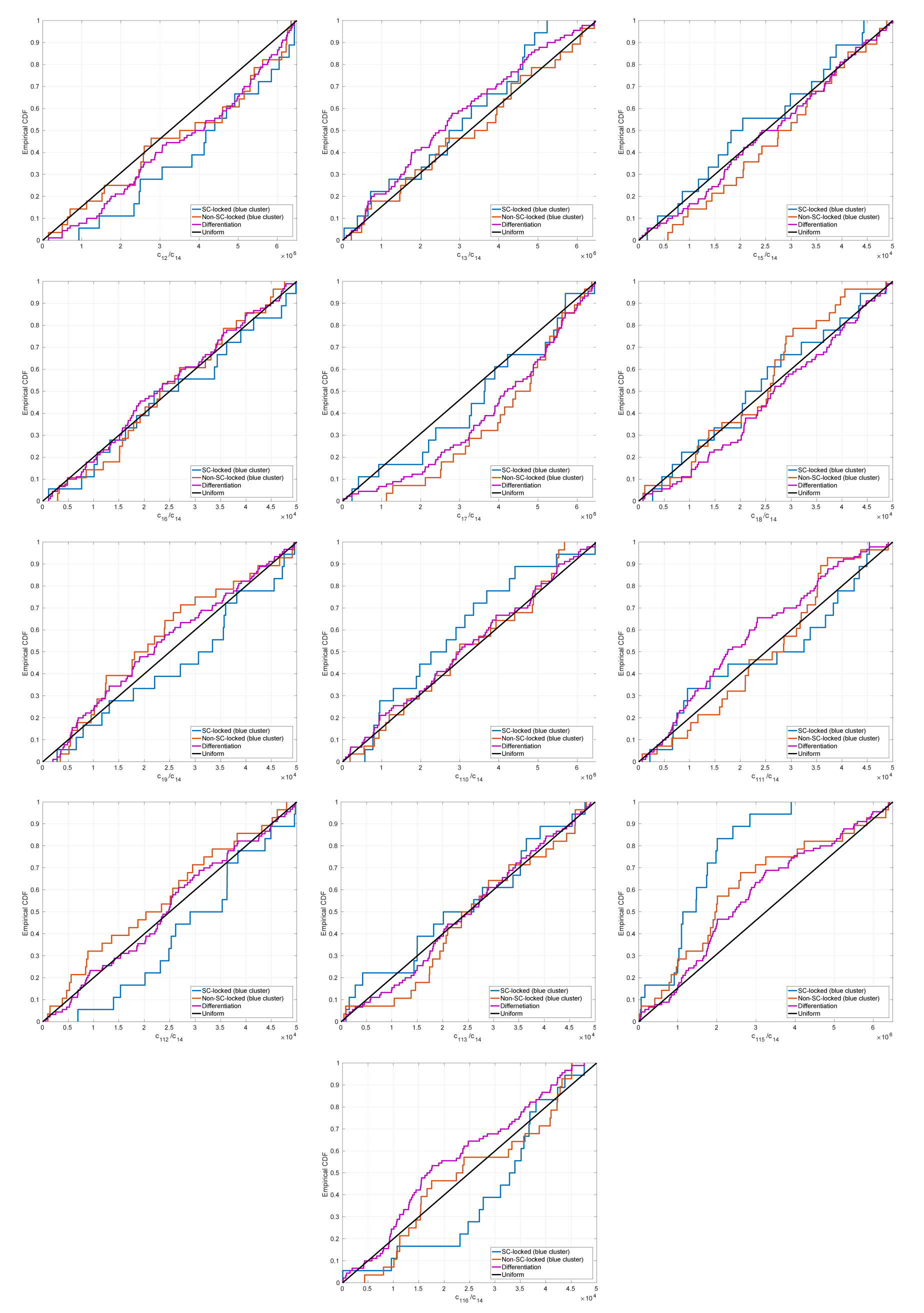

Supplement: S7 Fig — (TIF) [file pcbi.1006592.s009.tif]

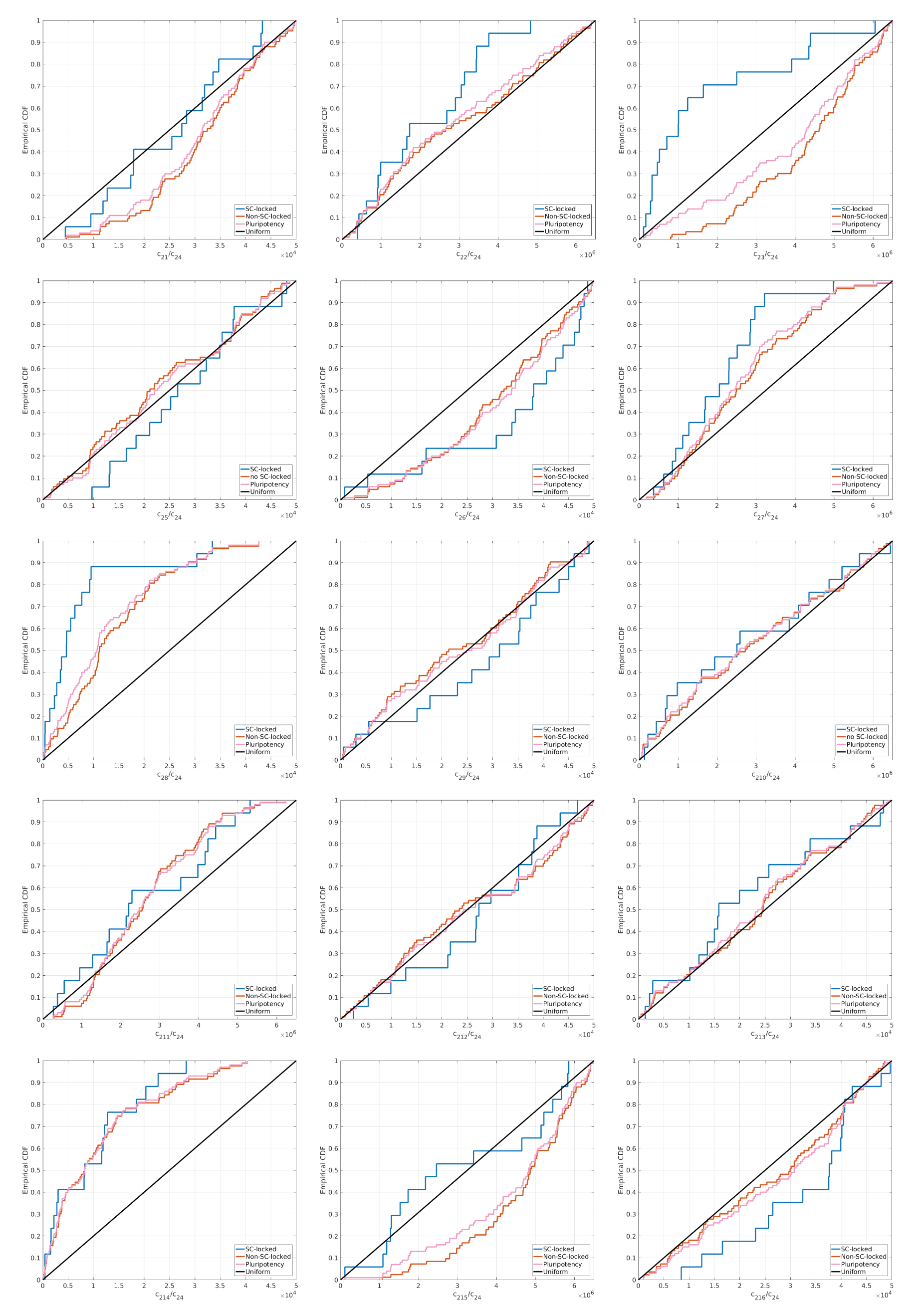

Supplement: S8 Fig — (TIF) [file pcbi.1006592.s010.tif]

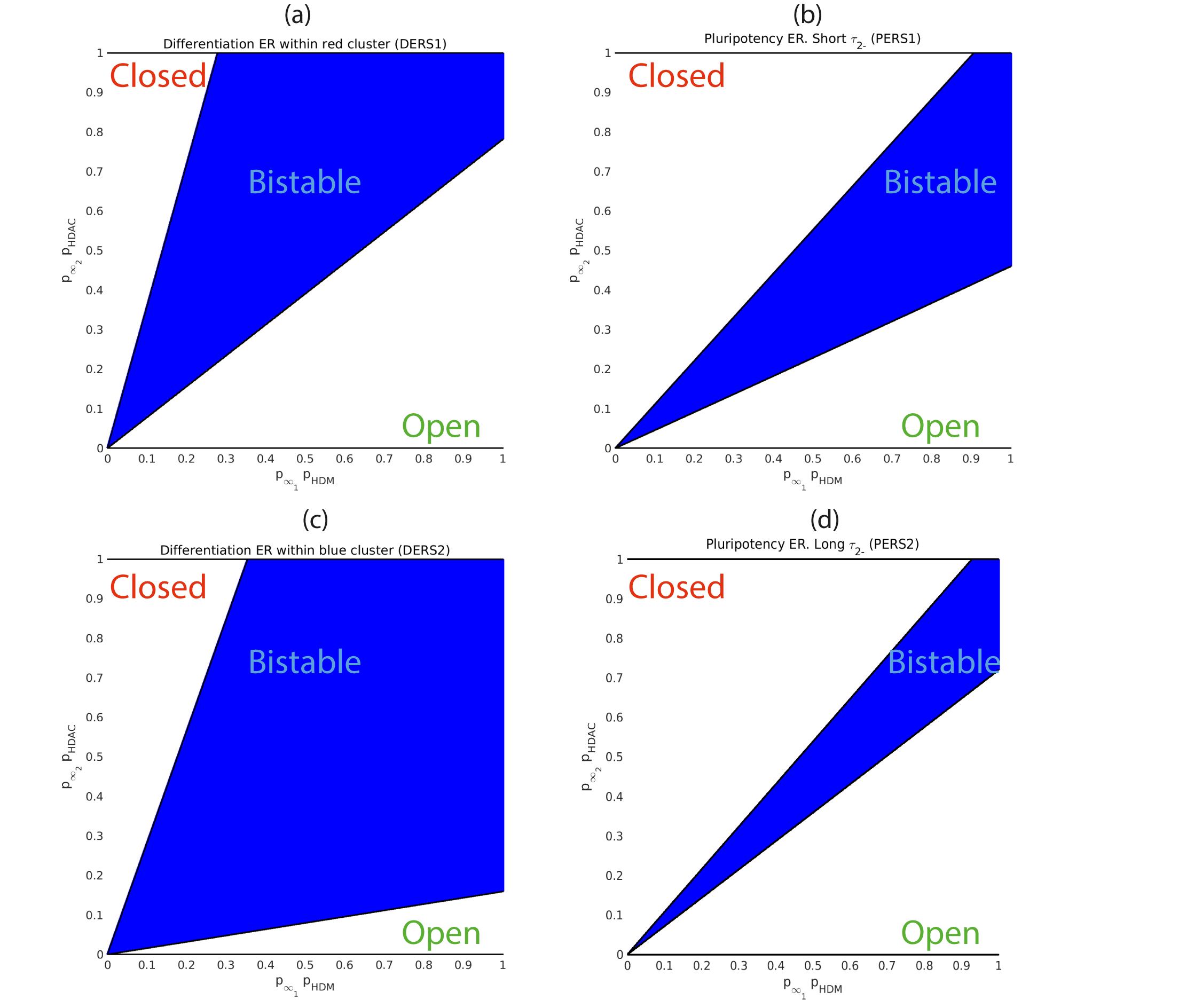

Supplement: S9 Fig — (TIF) [file pcbi.1006592.s011.tif]
