# Supplementary material for: A multiscale model of epigenetic heterogeneity-driven cell fate decision-making
Source: PLoS Comput Biol. 2019 Apr 30;15(4):e1006592. doi: 10.1371/journal.pcbi.1006592 (PMC6510448; doi:10.1371/journal.pcbi.1006592)
Supplement: S1 Table — (PDF) [file pcbi.1006592.s012.pdf]

Parameter	Description
N_G	Number of genes
R_E	Number of reactions in the ER model
S	Characteristic scale of the number of proteins
E	Characteristic scale of the number of binding sites in the promoter regions
Y	Characteristic scale of the number of binding sites for epigenetic marks
Z	Characteristic scale of the number of epigenetic enzymes
e_i	Number of binding sites in the promoter region of gene $i = 1, \ldots, N_G$
e_{HDM}	Number of HDM molecules
e_{HDAC}	Number of HDAC molecules
