# Supplementary material for: A multiscale model of epigenetic heterogeneity-driven cell fate decision-making
Source: PLoS Comput Biol. 2019 Apr 30;15(4):e1006592. doi: 10.1371/journal.pcbi.1006592 (PMC6510448; doi:10.1371/journal.pcbi.1006592)
Supplement: S2 Table — (PDF) [file pcbi.1006592.s013.pdf]

Variables	Description
X_i	Number of transcription factor monomers of type $i = 1, \ldots, N_G$
X_{ij}	Number of sites of the promoter region of gene i bound to a dimer of proteins of type j
**Parameters**	**Description**
$\hat{R}_i$	Basal rate of induction of gene $i = 1, \ldots, N_G$
k_{i1}	Rate of transcription of gene $i = 1, \ldots, N_G$
k_{i2}	Degradation rate of the protein of type $i = 1, \ldots, N_G$
b_{ij}	Binding rate of the homodimers of protein of type j onto the promoter region of gene i
u_{ij}	Unbinding rate of the homodimers of protein of type j from the promoter region of gene i
**Transition rates**	**Event**
$W_{i1}(X) = \hat{R}_i + k_{i1} X_{ii}$	$X_i \to X_i + 1,\ i = 1, \ldots, N_G$
$W_{i2}(X) = k_{i2} X_i$	$X_i \to X_i - 1,\ i = 1, \ldots, N_G$
$W_{i3}(X) = b_{ij} H(Y_{i3} - Y_0) \left(e_i - \sum_{k=1}^{N_G} X_{ik} \right) X_j (X_j - 1)$	$X_j \to X_j - 2,\ X_{ij} \to X_{ij} + 1,\ i, j = 1, \ldots, N_G$
$W_{i4}(X) = u_{ij} X_{ij}$	$X_j \to X_j + 2,\ X_{ij} \to X_{ij} - 1,\ i, j = 1, \ldots, N_G$
**Rescaled variables**	**Dimensionless parameters**
$\tau = b_{11} E S t$	$\epsilon_1 = \frac{E}{S}$ $\quad R_i = \frac{\hat{R}_i}{b_{11} E S^2}$
$x_i \equiv q_i = \frac{X_i}{S}$ (slow GRN variables)	$\omega_{i1} = \frac{k_{i1}}{b_{11} S^2}$ $\quad \omega_{i2} = \frac{k_{i2}}{b_{11} E S}$
$x_{ij} \equiv q_{ij} = \frac{X_{ij}}{E}$ (fast GRN variables)	$\beta_{ij} = \frac{b_{ij}}{b_{11}}$ $\quad \delta_{ij} = \frac{u_{ij}}{b_{11} S^2}$
