# Supplementary material for: A multiscale model of epigenetic heterogeneity-driven cell fate decision-making
Source: PLoS Comput Biol. 2019 Apr 30;15(4):e1006592. doi: 10.1371/journal.pcbi.1006592 (PMC6510448; doi:10.1371/journal.pcbi.1006592)
Supplement: S3 Table — (PDF) [file pcbi.1006592.s014.pdf]

Variables	Description	
Y_{i1}	Number of unmodified nucleosomes (U-nucleosome) associated with the ER system of gene i	
Y_{i2}	Number of methylated nucleosomes (M-nucleosome) associated with the ER system of gene i	
Y_{i3}	Number of acetylated nucleosomes (A-nucleosome) associated with the ER system of gene i	
Y_{i4}	Number of free HDM enzyme molecules associated with the ER system of gene i	
Y_{i5}	Number of methylated nucleosome-HDM enzyme complexes associated with the ER system of gene i	
Y_{i6}	Number of free HDAC enzyme molecules associated with the ER system of gene i	
Y_{i7}	Number of acetylated nucleosome-HDAC enzyme complexes associated with the ER system of gene i	
Parameter	Description	
c_{ij}	Kinetic rate of the jth reaction corresponding to the ER system of gene i	
Transition rate	Reaction change vector	Event
$V_{i1} = c_{i1}Y_{i2}Y_{i4}$	$r_{E_{i_1}} = (0, -1, 0, -1, +1, 0, 0)$	Formation of M-nucleosome-HDM enzyme complex (unrecruited)
$V_{i2} = c_{i2}Y_{i5}$	$r_{E_{i_2}} = (0, +1, 0, +1, -1, 0, 0)$	M-nucleosome-HDM enzyme complex splits (unrecruited)
$V_{i3} = c_{i3}Y_{i5}$	$r_{E_{i_3}} = (+1, 0, 0, +1, -1, 0, 0)$	Demethylation and HDM enzyme release (unrecruited)
$V_{i4} = c_{i4}Y_{i2}Y_{i3}Y_{i4}$	$r_{E_{i_4}} = (0, -1, 0, -1, +1, 0, 0)$	Formation of M-nucleosome-HDM enzyme complex (recruited)
$V_{i5} = c_{i5}Y_{i3}Y_{i5}$	$r_{E_{i_5}} = (0, +1, 0, +1, -1, 0, 0)$	M-nucleosome-HDM enzyme complex splits (recruited)
$V_{i6} = c_{i6}Y_{i3}Y_{i5}$	$r_{E_{i_6}} = (+1, 0, 0, +1, -1, 0, 0)$	Demethylation and HDM enzyme release (recruited)
$V_{i7} = c_{i7}Y_{i1}$	$r_{E_{i_7}} = (-1, +1, 0, 0, 0, 0, 0)$	Methylation (unrecruited)
$V_{i8} = c_{i8}Y_{i1}Y_{i2}$	$r_{E_{i_8}} = (-1, +1, 0, 0, 0, 0, 0)$	Methylation (recruited)
$V_{i9} = c_{i9}Y_{i3}Y_{i6}$	$r_{E_{i_9}} = (0, 0, -1, 0, 0, -1, +1)$	Formation of A-nucleosome-HDAC enzyme complex (unrecruited)
$V_{i10} = c_{i10}Y_{i7}$	$r_{E_{i_{10}}} = (0, 0, +1, 0, 0, +1, -1)$	A-nucleosome-HDAC enzyme complex splits (unrecruited)
$V_{i11} = c_{i11}Y_{i7}$	$r_{E_{i_{11}}} = (+1, 0, 0, 0, 0, +1, -1)$	Deacetylation and HDAC enzyme release (unrecruited)
$V_{i12} = c_{i12}Y_{i3}Y_{i2}Y_{i6}$	$r_{E_{i_{12}}} = (0, 0, -1, 0, 0, -1, +1)$	Formation of A-nucleosome-HDAC enzyme complex (recruited)
$V_{i13} = c_{i13}Y_{i7}Y_{i2}$	$r_{E_{i_{13}}} = (0, 0, +1, 0, 0, +1, -1)$	A-nucleosome-HDAC enzyme complex splits (recruited)
$V_{i14} = c_{i14}Y_{i7}Y_{i2}$	$r_{E_{i_{14}}} = (+1, 0, 0, 0, 0, +1, -1)$	Deacetylation and HDAC enzyme release (recruited)
$V_{i15} = c_{i15}Y_{i1}$	$r_{E_{i_{15}}} = (-1, 0, +1, 0, 0, 0, 0)$	Acetylation (unrecruited)
$V_{i16} = c_{i16}Y_{i1}Y_{i3}$	$r_{E_{i_{16}}} = (-1, 0, +1, 0, 0, 0, 0)$	Acetylation (recruited)
Rescaled variables	Dimensionless parameters	
$y_{ij} = \frac{Y_{ij}}{Y}$ $j = 1, 2, 3$ (slow variables)	$\kappa_{ij} = \frac{c_{ij}}{c_{14}E}$ $j = 1, 5, 6, 9, 13, 14$	$\kappa_{ij} = \frac{c_{ij}}{c_{14}EZ}$ $j = 7, 15$
$y_{ij} = \frac{Y_{ij}}{Z}$ $j = 4, 5, 6, 7$ (fast variables)	$\kappa_{ij} = \frac{c_{ij}}{c_{14}E^2}$ $j = 2, 3, 10, 11$	$\kappa_{ij} = \frac{c_{ij}}{c_{14}Z}$ $j = 8, 16,$ $\kappa_{ij} = \frac{c_{ij}}{c_{14}}$ $j = 4, 12$
