# Supplementary material for: A multiscale model of epigenetic heterogeneity-driven cell fate decision-making
Source: PLoS Comput Biol. 2019 Apr 30;15(4):e1006592. doi: 10.1371/journal.pcbi.1006592 (PMC6510448; doi:10.1371/journal.pcbi.1006592)
Supplement: S4 Table — (PDF) [file pcbi.1006592.s015.pdf]

Rescaled parameter	Value for DERSs (dimensionless)	Value for PERSs (dimensionless)
κ_{i1}	200	200
κ_{i2}	100	100
κ_{i3}	50	10
κ_{i5}	1	1
κ_{i6}	200	10
κ_{i7}	10	100
κ_{i8}	100	100
κ_{i9}	200	200
κ_{i10}	100	100
κ_{i11}	0.1	10
κ_{i12}	1	1
κ_{i13}	1	1
κ_{i14}	200	100
κ_{i15}	100	100
κ_{i16}	100	100
