# Supplementary material for: A multiscale model of epigenetic heterogeneity-driven cell fate decision-making
Source: PLoS Comput Biol. 2019 Apr 30;15(4):e1006592. doi: 10.1371/journal.pcbi.1006592 (PMC6510448; doi:10.1371/journal.pcbi.1006592)
Supplement: S5 Table — (PDF) [file pcbi.1006592.s016.pdf]

Rescaled parameter	DERS1 values	DERS2 values	PERS1 values	PERS2 values
κ_{i1}	40.951008	21.022829	121.624100	107.745895
κ_{i2}	33.545677	37.389736	14.942093	14.304025
κ_{i3}	36.101627	6.075892	90.265221	52.221851
κ_{i5}	71.377869	119.379074	135.026962	172.351425
κ_{i6}	45.063740	199.229645	137.927872	173.544327
κ_{i7}	2806.709717	2635.588623	1652.600464	1692.524658
κ_{i8}	7397.834961	4078.342285	2235.608398	6550.556641
κ_{i9}	170.622452	142.806244	123.194839	47.068409
κ_{i10}	77.820129	50.091576	47.182499	43.554352
κ_{i11}	0.093411	0.435084	32.408936	41.708683
κ_{i12}	6848.829102	6921.437988	34383.695312	949.391724
κ_{i13}	131.147171	11.198260	102.787231	31.121500
κ_{i14}	9.915549	8.374920	157.155838	50.545132
κ_{i15}	3712.249268	1926. 709595	5133.064453	5126.510254
κ_{i16}	2623.266846	7136.016602	6294.414551	5547.370605
