# Supplementary material for: A multiscale model of epigenetic heterogeneity-driven cell fate decision-making
Source: PLoS Comput Biol. 2019 Apr 30;15(4):e1006592. doi: 10.1371/journal.pcbi.1006592 (PMC6510448; doi:10.1371/journal.pcbi.1006592)
Supplement: S6 Table — (PDF) [file pcbi.1006592.s017.pdf]

Rescaled parameter	Value (dimensionless)
κ_{i1}	70.205910
κ_{i2}	73.419846
κ_{i3}	3.609020
κ_{i5}	119.968636
κ_{i6}	107.726303
κ_{i7}	5100.981445
κ_{i8}	4037.399658
κ_{i9}	40.685452
κ_{i10}	78.823647
κ_{i11}	0.785620
κ_{i12}	43095.195312
κ_{i13}	55.197235
κ_{i14}	17.289341
κ_{i15}	5054.711914
κ_{i16}	7180.712402
