# Supplementary material for: A multiscale model of epigenetic heterogeneity-driven cell fate decision-making
Source: PLoS Comput Biol. 2019 Apr 30;15(4):e1006592. doi: 10.1371/journal.pcbi.1006592 (PMC6510448; doi:10.1371/journal.pcbi.1006592)
Supplement: S7 Table — (PDF) [file pcbi.1006592.s018.pdf]

Rescaled parameter	Parameter	Units	Reference
$\omega_{11} = \omega_{21} = \frac{a}{k_{deg}\sqrt{K_d}}$	$K_d = 10$	nM	[1]
$\omega_{21} = \omega_{22} = 1$	$k_{deg} = 2$	min^{-1}	[1]
$\delta_{ij} = 1$ for all i, j	$r = 0.4$	$\text{nM} \cdot \text{min}^{-1}$	[1]
$\beta_{ij} = 1$ for all i, j			–
$R_1 = R_2 = \frac{r}{k_{deg}\sqrt{K_d}}$	$S = 1000$		–
$b_{11}ES = k_{deg}$	$E = 5$		–
	$e_1 = 5$		–
	$e_2 = 5$		–

References

1. Frigola D, Casanellas L, Sancho JM, Ibañes M. Asymmetric stochastic switching driven by intrinsic molecular noise. PLoS One. 2012;7:e31407.
